# Reacquisition of the lower temporal bar in sexually dimorphic fossil lizards provides a rare case of convergent evolution

**DOI:** 10.1038/srep24087

**Published:** 2016-04-13

**Authors:** Tiago R. Simões, Gregory F. Funston, Behzad Vafaeian, Randall L. Nydam, Michael R. Doschak, Michael W. Caldwell

**Affiliations:** 1 Department of Biological Sciences, University of Alberta, Edmonton, Alberta T6G 2E9, Canada; 2 Department of Civil and Environmental Engineering, University of Alberta, Edmonton, Alberta T6G 2E9, Canada; 3 Department of Anatomy, Arizona College of Osteopathic Medicine, Midwestern University, 19555 N. 59th Dr., Glendale, AZ 85383, USA; 4 Faculty of Pharmacy & Pharmaceutical Sciences, University of Alberta, Edmonton, Alberta T6G 2E1, Canada; 5 Department of Earth and Atmospheric Sciences, University of Alberta, Edmonton, Alberta T6G 2E9, Canada

## Abstract

Temporal fenestration has long been considered a key character to understand relationships amongst reptiles. In particular, the absence of the lower temporal bar (LTB) is considered one of the defining features of squamates (lizards and snakes). In a re-assessment of the borioteiioid lizard *Polyglyphanodon sternbergi* (Cretaceous, North America), we detected a heretofore unrecognized ontogenetic series, sexual dimorphism (a rare instance for Mesozoic reptiles), and a complete LTB, a feature only recently recognized for another borioteiioid, *Tianyusaurus zhengi* (Cretaceous, China). A new phylogenetic analysis (with updates on a quarter of the scorings for *P. sternbergi*) indicates not only that the LTB was reacquired in squamates, but it happened independently at least twice. An analysis of the functional significance of the LTB using proxies indicates that, unlike for *T. zhengi*, this structure had no apparent functional advantage in *P. sternbergi*, and it is better explained as the result of structural constraint release. The observed canalization against a LTB in squamates was broken at some point in the evolution of borioteiioids, whereas never re-occuring in other squamate lineages. This case of convergent evolution involves a mix of both adaptationist and structuralist causes, which is unusual for both living and extinct vertebrates.

The repeated independent evolution of similar characteristics (homoplasies) is an extremely important and under-investigated phenomenon^1^. Homoplasies, such as evolutionary convergences, are often seen as strong evidence for adaptative evolution^2^,^3^ because similar environmental pressures are expected to elicit similar adaptative morphologies, suggesting that phenotypic homoplasy is often a consequence of natural selection^4^. This is reinforced by the theory of historical contingency^5^, which suggests that the repeated evolution of very similar structures is rather rare in the history of life.

Possible convergences on the loss, or gain, of temporal fenestration have been a major issue in the study of reptile evolution in recent years, such as the loss of such fenestration in the evolution of the turtle skull^6^,^7^,^8^,^9^. The lower temporal bar (LTB) contributes to the formation of a lower temporal fenestra in diapsid reptiles, and is thought to have been absent in the early evolution of lepidosaurs^10^,^11^,^12^, a group that includes *Sphenodon* and squamates, and later reacquired in some rhynchocephalians (*Sphenodon* and its extinct relatives^12^,^13^,^14^,^15^). Squamates have long been thought to lack a complete LTB, and this feature has been used numerous times as a defining character of the group^16^,^17^,^18^,^19^. Recently, this notion was challenged by the discovery of *Tyaniusaurus zhengi*, a borioteiioid lizard from the Late Cretaceous of Asia with a complete bony LTB^20^. Understanding the evolution of temporal fenestration is thus fundamental to understand broad level relationships amongst living and extinct groups of reptiles, as well the phenomenon of evolutionary convergence. In that regard, only a limited number of studies have been dedicated to the evolution (including evolutionary convergence) of the lower temporal bar in lepidosaurian reptiles using modern analytical tools (e.g.^10^,^15^,^21^).

Here we report the discovery of a complete LTB forming an enclosed lower temporal fenestra in another borioteiioid lizard, *Polyglyphanodon sternbergi*, the most complete lizard known to date from the Mesozoic of North America. Even with such a rich osteological record, we noted several discrepancies between the original description and the morphology as observed by us (including the presence of a complete LTB). These materials include an ontogenetic series, as well as variation in skull shape that can be linked to sexual dimorphism, which provides a rare insight into the paleobiology of an entirely extinct clade of lizards, the borioteiioids. Furthermore, the repeated occurrence of a complete LTB served as the basis for a broader analysis on the evolution of this structure in lepidosaurs, with emphasis on squamate reptiles.

## Results

### Morphology, ontogeny and sexual dimorphism

**Figure 1:**
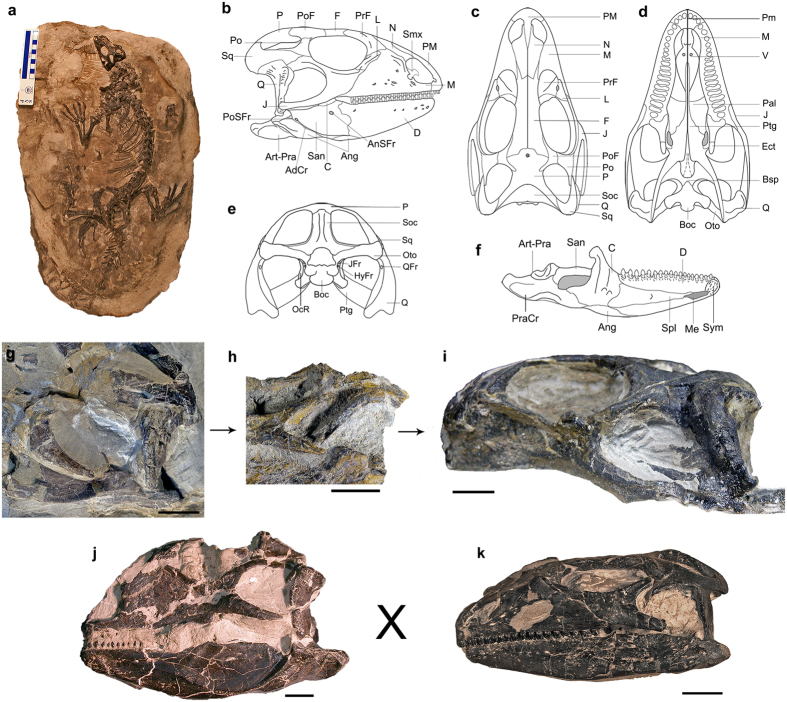
*Polyglyphanodon sternbergi*, from the Late Cretaceous of Utah (USA). (**a**) CM 9188 in dorsal view (image credits to Amy Henrici); (**b–f**) anatomy of the skull, reconstructed from all the specimens used in this study (image credits to Arthur Brum); (**g–i**) ontogeny of the temporal region, illustrating the relative increase in length of the LTB in *P. sternbergi*, from juvenile (**g**), NMNH 427672 and (**h**), NMNH 16586) to adult (**i**), NMNH 15816); (**j**) NMNH 16587, representing the sexual morphotype (**a,k**) CM 9188, sexual morphotype (**b**). Abbreviations: Art-Pra, articular + prearticular; AdCr, adductor crest of surangular; Ang, angular; AnSFr, anterior surangular foramen; Boc, basioccipital; Bsp, basipterygoid; C, coronoid; D, dentary; Ect, ectopterygoid; F, frontal; HyFr, hypoglossal foramina; J, jugal, JFr; jugular foramen; L, lacrimal; M, maxilla; Me, Meckelian canal; N, nasal; P, parietal; OcR, occipital recess; Oto, otoccipital; Pal, palatine; PM, premaxilla; Po, postorbital; PoF, postfrontal; PoSFr, posterior surangular foramen; PraCr, prearticular crest; PrF; prefrontal; Ptg, pterygoid; Q, quadrate; QFr, quadrate foramen; San, surangular; Smx, septomaxilla; Soc, supraoccipital; Spl, splenial; Sq, squamosal; Sym, dentary symphysis; V, vomer. Scale bars equal to 10 mm (**g–k**).

From the examination of almost 30 specimens of *Polyglyphanodon sternbergi*, including almost complete skeletons, we obtained new information on the morphology of that species, especially regarding the skull (Fig. 1a–f). Individuals of different size classes present variation that is concentrated on the shape of the frontoparietal suture and the length of the posteroventral process of the jugal (Fig. 1g–i). Great variation in the shape of the frontoparietal suture has been reported during the post-embryonic ontogeny of extant lizards^22^,^23^ and variation on the length of the jugal process has also been reported during the ontogeny of fossil sphenodontian reptiles^13^,^14^,^24^ (see also Table 1 and Supplementary Discussion). To our knowledge, neither have ever been detected before in fossil lizards. Furthermore, specimens of similar sizes present variations in skull shape. Overall, some individuals possess relatively taller skulls (morphotype A—Fig. j) while others have more depressed skulls (morphotype B—Fig. 1k). Such variation happens across individuals of all size classes (Table 1) and thus cannot be related to ontogeny. Sexual dimorphism in lizards commonly affects body proportions, with male lizards tending to have proportionally bigger heads—either taller, longer, or wider, or a combination of these^25^,^26^,^27^—for male-male combat or holding females during copulation (see also Supplementary Discussion). Therefore, the variation in relative skull height between both morphotypes is suggestive of sexual dimorphism, with morphotype A (proportionally taller skulls) being more likely to represent the male morphotype. All *Polyglyphanodon* individuals come from the same mudstone horizon, in what is considered as the flood basin of a fluvial system (RN, personal observation), being totally or partially articulated. This is indicative that they represent a local population that was caught in a flood, or another similarly catastrophic event, and thus finding individuals of different age and sex classes should be expected.

In all specimens, the jugal bone (when preserved) was usually broken posteriorly. One exception was the paratype (NMNH 15816), which has a complete posteroventral process of the jugal extending posteriorly to the quadrate, forming a complete LTB (Fig. 1i). In a second specimen (NMNH 16588, not figured), despite the posteriormost tip of the jugal being broken, the preserved portion also extended to the level of the quadrate articulation with the mandible. In both *P. sternbergi* and *T. zhengi*, the LTB differs from the condition observed in other reptiles. Whereas in archosaur reptiles (e.g. crocodilians, dinosaurs and pterosaurs) and *Sphenodon*, the LTB is formed by the jugal and also the quadratojugal, the LTB in the two fossil lizard species is formed exclusively by the jugal, as the quadratojugal is absent in all squamates known so far. Furthermore, juvenile skulls of *P. sternbergi* with a complete jugal have the LTB shorter than in adults and not reaching the quadrate, differing from archosaurs and *Sphenodon*, but similar to the condition reported for other rhynchocephalians: *Clevosaurus* and *Planocephalosaurus*^14^,^24^.

**Figure 2:**
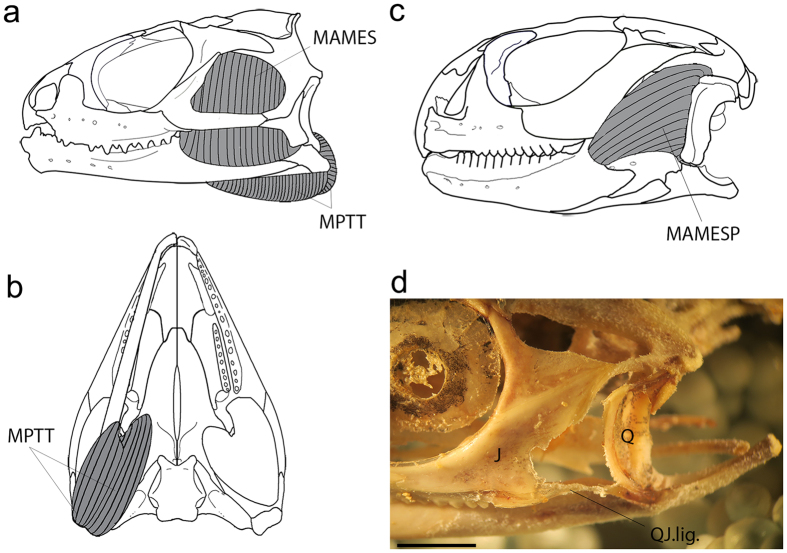
Temporal region in lepidosaurs. (**a**) skull of *Sphenodon punctatus* in lateral view; (**b**) skull of *Sphenodon punctatus* in ventral view; (**c**) skull of *Uromastyx aegyptius* in lateral view; (**d**) temporal region of *Agama agama* in lateral view. Abbreviations: MAMES, *M. adductor mandibularis externus superficialis*; MAMESP, *M. adductor mandibularis externus superficialis posterior*; MPTT, *M. pterygoideus typicus*; J, jugal; Q, quadrate; QJ.lig., quadratojugal ligament.

Despite the similarities noted above, the structure of the LTB in *P. sternbergi* differs from the one in *T. zhengi*, as in the latter the bar is firmly sutured to the quadrate, whereas in *P. sternbergi* there is no discernable articulatory facet on the quadrate. Additionally, on the ventrolateral face of the quadrate tympanic crest there is a rugose surface similar to the one on the cephalic condyle, at the level of the posteroventral process of the jugal (Supplementary Fig. S1). This suggests there was a soft tissue connection between the jugal and the quadrate, likely formed by a reduced quadratojugal ligament, which also connects the jugal to the quadrate in some extant lizard species, such as *Corucia zebrata*^28^. In a large number of lizards and snakes, however, this temporal ligament does not contact the quadrate, but rather the mandible^28^,^29^, and it is termed a jugomandibular ligament; in such cases there are no rugose surfaces on the quadrate ventrolateral margin. We exclude the possibility of this being an attachment site for the *Musculus adductor mandibulae externus superficialis* (MAMES—Fig. 2) because of the similarity in texture between this surface and the one on the cephalic condyle, as well as the absence of such rugosity on the tympanic crest of observed specimens of *Tupinambis teguixin*, a taxon in which the MAMES is extremely well developed.

Another peculiar feature is that the LTB in *P. sternbergi* is straight, rather than bowed laterally (the latter being the condition in *Sphenodon*), indicating that the posterior portion of the MAMES (MAMESP) was relatively modest with respect to its’ maximum width (cross-sectional area), unlike the condition usually observed in squamates^21^,^30^,^31^ (Fig. 2c and Supplementary Fig. S2). However, the ventrally located adductor crest in the lower jaw of *P. sternbergi* indicates that the MAMES extended some distance ventrally on the lateral side of the lower jaw. This ventral expansion is greater than the one seen in *Iguana iguana*, for instance, and would more closely resembles the ventral expansion observed in *Tupinambis*^21^.

### Phylogeny

**Figure 3:**
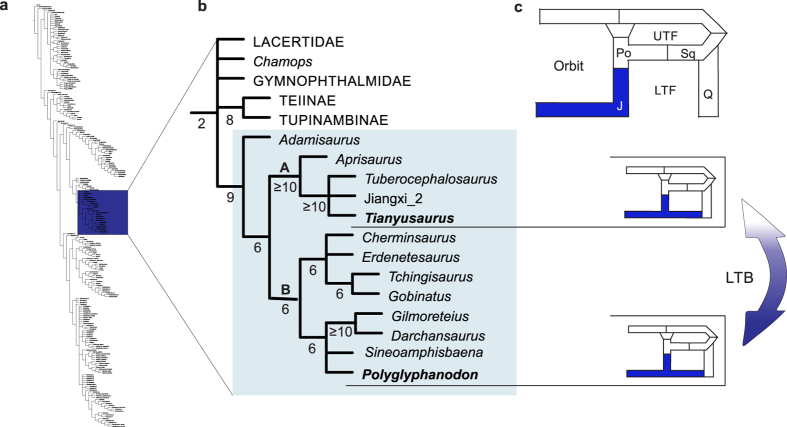
Phylogenetic position of *P. sternbergi* and *T. zhengi* amongst borioteiioid lizards. The phylogenetic position of borioteiioids within squamates was not altered in relation to previous analyses of this dataset^32^,^52^,^53^. (**a**) Abbreviated view of strict consensus tree depicting all major squamate lineages, with dark blue box highlighting the position of Lacertiformes. (**b**) Recovered relationships among Lacertiformes, with borioteiioids (or Polyglyophanodontidae *sensu* Conrad^52^) highlighted by the light blue box. Strict consensus of 54,880 most parsimonious trees (consistency index = 0.1520; retention index = 0.7158) of 3,521 steps. Numbers below branches indicate absolute Bremer indices. (**c**) Schematic drawing of composition of the lower temporal region as observed in most lizards, depicting the independent development (connected by the arrow) of a complete LTB formed by the jugal (blue) in *P. sternbergi* and *T. zhengi*. Abbreviations: J, jugal; LTF, lower temporal fenestra, Po, postorbital; Q, quadrate; Sq, squamosal, UTF, upper temporal fenestra.

After a revision of the scorings for *P. sternbergi* in a data matrix inclusive of all major clades of squamates, which also contained *T. zhengi* and other borioteiioids^32^, more than a quarter (27%) of the osteological characters for *P. sternbergi* were altered or scored for the first time (see Methods). The results obtained indicate *T. zhengi* groups with other Chinese borioteiioids (clade A—Fig. 3), whereas *P. sternbergi* belongs to a separate clade (clade B—Fig. 3), indicating the lower temporal bar evolved convergently in *P. sternbergi* and *T. zhengi*. As a consequence, the lower temporal bar not only redeveloped amongst squamates, as recently demonstrated by the discovery of *Tianyusaurus*, but it actually happened twice within the same group of squamates—the Borioteiioidea.

### The evolution of the LTB as a functional adaptation

Squamates have a posterior portion—or 1b layer^31^—of the MAMES (or MAMESP) that is differentiated from the anterior portion of the same muscle, becoming wider and more expanded ventrally and posteriorly on the lateral side of the lower jaw^21^,^30^,^31^ than the MAMES in other reptiles in which the LTB is present^21^. In some instances, however (e.g. *Iguana iguana*) that portion is wider and expanded posteriorly, but it does not extend far ventrally in comparison to the MAMES of *Sphenodon*^33^. The configuration of the squamate skull provides this muscle with a more efficient adductor function than the *M. pterygoideus typicus* (MPTT—Fig. 2) and *atypicus* (MPTAT—absent in squamates) of other reptiles, and thus, the loss of the LTB has been considered a selective advantage for allowing more room for an expanded MAMES and a more efficient mandible adductor system^21^. Measurements of the cross-sectional area of the MAMES in *Sphenodon*, along with *in vivo* bite forces, have confirmed *Sphendon* has a smaller adductor muscle mass and bite forces than similar sized agamid lizards^15^. These changes provided squamates with a relatively greater bite force in comparison to other reptiles, to the point that a lizard scaled up to the size of a *Tyrannosaurus rex* would be capable of biting ten times harder than *T. rex*^34^.

The loss of the lower temporal bar may lead to functional disadvantages, however. In crocodilians, for instance, this bar promotes stabilization of the quadrate bone, without which, would tend to rotate anteriorly due to the resultant force of action of the temporal muscles. The same has been described for *Sphenodon*^11^,^15^. This would cause interference of the quadrate upon the proper functioning of the temporal muscles just anterior to it (including the MAMES). Lizards and other squamates do not face this problem because of differences in the skeletomuscular configuration of the temporal region, creating a resultant force of the temporal musculature that is directed posterodorsally during jaw closure, and not anterodorsally. For this reason, the quadrate in squamates usually tends to rotate posteriorly^28^. Therefore, a jugomandibular ligament (or quadratojugal ligament in some species, such as *Corucia*^28^ and *Agama*—Fig. 2d) provides stabilization of the quadrate as tension is applied to this ligament. Furthermore, the elastic nature of the ligament allows for a large sized posteroventral portion of the MAMES. In this way, squamates were able to maintain a stable quadrate while also increasing the size of the temporal musculature^21^,^28^,^35^.

This indicates that the reacquisition of the lower temporal bar could be a consequence of either one of the following factors: I) a change in orientation of the temporal bones and muscles creating resultant forces at the quadrato-mandibular joint during biting that would tend to rotate the quadrate anteriorly, similar to archosaurs and thus interfering with proper adductor musculature functioning^28^. In this situation, a ligament would not be enough to stabilize the quadrate, as it can only work properly for such a function when the force of action tends to strain the ligament, applying tension to it^10^,^28^; II) the stabilization of the quadrate by the suspensorium (dorsally) and pterygoids (ventrally), such as is observed in rhynchocephalians and archosaurs, may provide only limited distribution of stress and/or compressive-tensional forces during hard biting for some species. In some instances, the re-development of a complete LTB could reduce stress and/or strain in the skull^10^,^33^.

### Reacquisition of the LTB in lepidosaurian reptiles–rejected hypotheses

Some of the first hypotheses for the re-development of the LTB in lepidosaurs tried to suggest it as a bracing mechanism to maintain a stable quadrate for precise shearing action^11^,^13^,^14^. However, in rhynchocephalians (e.g. *Diphyodontosaurus* and *Planocephalosaurus*) in which the quadrate is fixed, the latter is stable enough and able to maintain precise shearing even in earlier ontogenetic stages that lack a complete LTB. The same applies to squamates with precise shearing actions despite lacking a complete LTB, such as *Iguana iguana*^36^. Even in forms with a mobile quadrate (e.g. *Uromastyx acanthinurus*) precise shearing takes place^36^,^37^.

It has also been suggested that the role of the LTB was to stabilize the quadrate in order to develop translational movements of the jaw observed during proal shearing in *Sphenodon*^15^. This suggestion has been discarded in previous studies based on similar comparisons to fossil rhynchocephalians. For instance, *Gephyrosaurus* and *Priosphenodon* display morphological features indicative of translational movement of the jaw, despite the lack a complete LTB in those species^33^. Therefore, we reject such hypotheses as likely functional explanations for the re-acquisition of the LTB (see more in Supplementary Discussion).

### Rejection of functional explanations in *P. sternbergi*

*Polyglyphanodon sternbergi* has an anteriorly arched frontoparietal suture in dorsal aspect that would be likely to prevent mesokinesis in adults (Fig. 1 and Supplementary Fig. S3). It also has an enlarged articulation surface between the parietal and the supraoccipital, which would prevent metakinesis—Fig. 1, and also illustrated by Gauthier *et al*.^38^. The increased contact between the quadrate, the squamosal and the jugal anterodorsally, as well as the pterygoids ventromedially (Fig. 1 and Supplementary Fig. S4), also prevented streptostyly. Therefore, osteological information indicates *P. sternbergi* likely lacked any form of detectable cranial kinesis. The lack of streptostyly prevented quadrate rotation, and thus it could not have rotated anteriorly and interfere with the proper functioning of the MAMES. Furthermore, if the configuration of the temporal skeletomuscular system in *P. stermbergi* was similar to that of most lizards, its quadrate would usually tend to rotate posteriorly^28^.

Therefore, the only remaining and plausible hypothesis left to test is the reduction of mechanical stress during forceful biting, as previously proposed for *T. zhengi* and other reptiles with a fixed quadrate^10^. To empirically test whether or not the complete LTB in *P. sternbergi* is an adaptation for reduction of mechanical stress, we tested the biomechanical significance of a complete LTB in a lizard skull. A previous study had been performed to test this issue^10^, but in that case the LTB was always inferred to be sutured to the quadrate. In our analysis we included a model with a complete LTB connected to the quadrate by soft tissues (as seen in *P. sternbergi*)–a model that has never been investigated so far for squamate skulls.

### Biomechanics

**Figure 4:**
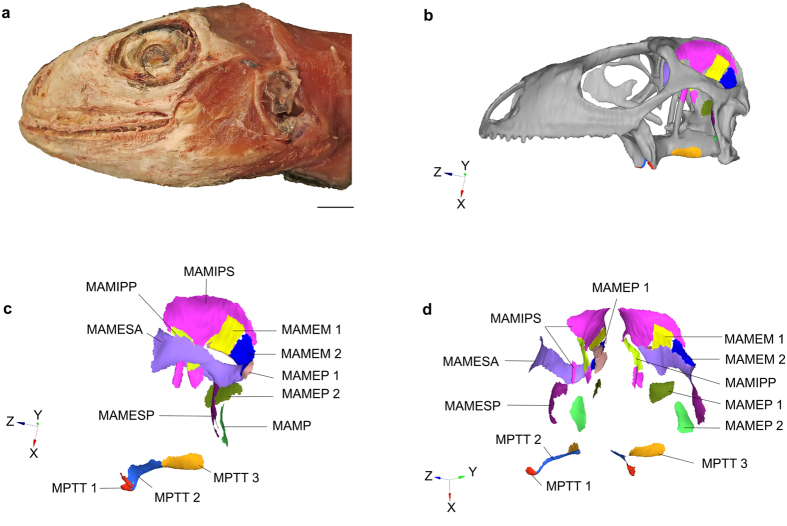
Creation of the models for the FEA. (**a**) specimen of *Iguana iguana* used for CT-scan and for subsequent dissection in order to model muscle origin attachment areas. (**b**) 3D volume mesh model of *I. iguana* used for the FEA and with muscles maps. (**c**) Muscle attachments in lateral aspect. (**d**) Muscle attachments in anterolateral aspect. Finite elements shape and number affects the shape of the borders of the muscle attachment areas, but these are minor and do not affect significantly the number of selected nodes for each muscle. Scale bar = 10 mm. Abbreviations: MAMEM, *M. adductor mandibulae externus medius*; MAMEP, *M. adductor mandibulae externus profundus*; MAMESA, *M. adductor mandibulae externus superficialis anterior*; MAMESP, *M. adductor mandibulae externus superficialis posterior*; MAMIPP, *M. adductor mandibulae internus pseudotemporalis profundus*; MAMIPS, *M. adductor mandibulae internus pseudotemporalis superficialis*; MAMP, *M. adductor mandibulae* posterior; MPTT, *M. pterygoideus typicus*. Scale bar (A and B) = 10 mm.

We performed a FEA on a skull of *Iguana iguana* which was used as a proxy to evaluate the stress on the skull of *Polyglyphanodon sternbergi* (see Methods). We tested hard-biting in the *Iguana* skull using 3D muscle attachment maps based on our own dissection (Fig. 4) for four distinct models: (A) the unmodified skull, as obtained from the micro-CT-scan geometry, with a tension-only spring between the jugal and quadrate representing a quadratojugal ligament; (B) the unmodified skull, but with two force vectors applied to the jugal bone and directed towards the lower jaw, representing a jugomandibular ligament. These two different ligament models were tested as both conditions are observed in lizards^28^ (see above); (C): the *Iguana* skull recreated with the addition of a complete LTB that was sutured to the quadrate, as previously performed for a similar analysis^10^; and (D), the *Iguana* skull with a complete LTB, but connected to the quadrate by a tension only spring (representing a ligament connection), which replicates the condition inferred for *P. sternbergi*. These models were tested for a range of muscle bite forces (see Supplementary Table S1 and S2), providing a total of 12 different analyses (see Supplementary Discussions on further considerations and limitations of the analysis).

In all models, variations in the magnitude of bite forces did not change the pattern of distribution of stress or strain in the skull. The resultant joint reaction forces acting on the quadrato-mandibular joint were found in the FEA to be always directed posterodorsally (Supplementary Fig. S5 and Supplementary Table S3), and the ligaments to be under tension, which is in agreement with previous studies^10^,^28^,^35^.

**Figure 5:**
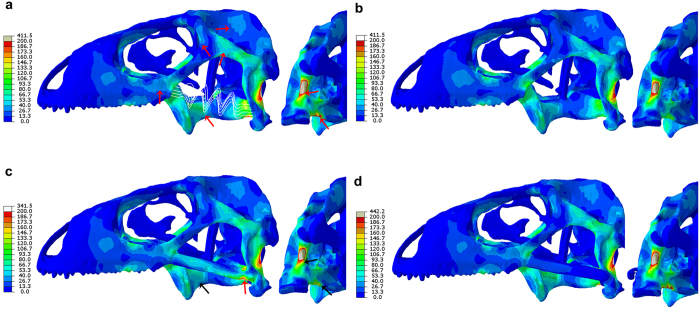
Results of the FEA of the skull of *Iguana iguana*, testing how distinct models for a LTB would affect mechanical stress during hard biting. Results are displayed as contours representing combined-axes von Mises stress. (**a**) model A, lizard with quadratojugal ligament; (**b**) model B, lizard with jugomandibular ligament; (**c**) model C, lizard with the addition of a complete LTB, sutured to the quadrate; (**d**) model D, lizard with the addition of a complete LTB, connected to the quadrate by a short quadratojugal ligament. Red arrows indicate the points in model “A” with increase of stress compared to model “B” (the ones with the least amount of increased stress regions); in model “C”, red and black arrows represent areas of increased and reduces stress, respectively, compared to model “A”. Model “D” had a very similar distribution of stress to model “A”.

Among the four models, model B was the best suited for forceful biting. Different regions of the skull showed reduced stress compared with the other models (Fig. 5), apart from a minor increased stress in the nasal process of the maxilla. Model A had comparable areas of the skull with increased stress, such as: the ventral margin of the orbit, parts of the upper temporal bar, the ventral side of the basisphenoid, and especially on the posterior crest of the quadrate, as well as the quadrate process of the pterygoid. The pressure maps also indicate greater compression in the upper temporal and postorbital bars, and greater compression and tension in the pterygoids for model A when compared to B. Von Mises stress values were particularly higher on the quadrate, and especially on the pterygoid (43.2% higher—Supplementary Table S4). Our results thus suggest that the jugomandibular ligament represents a derived condition in squamates, emerging in forms with robust akinetic skulls, built for strenuous biting, as previously hypothesized^28^.

In the two hypothetical models, which possess different kinds of LTBs (C and D), both models show results that are more similar to model A. Model C has greater stress compared to B, and similar patterns of compression and tension, in similar regions as model A. Despite the addition of the fused LTB creating a point of increased stress upon it posteriorly, the stress in the quadrate posterior crest and pterygoid close to the junction with the quadrate (regions under highest stress in model A), was reduced—21% less than model A in the pterygoid (although still 28% higher than model B). Therefore, model C (a lizard with a fused lower temporal bar, analogous to *T. zheng*) has reduction of stress compared to a model with a quadratojugal ligament, but still with more areas of increased and higher stress values than model B. Although the patterns of stress distribution by the addition of a LTB were generally the same as the ones using a *Uromastyx* model^10^, in the latter a fused LTB performed better for forceful biting than a model with jugomandibular ligaments. It remains to be assessed if these differences relate to the shape of the model under use, the difference in the application and distribution of load values, or another unknown variables. However, if other borioteiioids (including *T. zhengi*) also had the temporal ligaments connecting to the quadrate (our model A, and as inferred for *P. stermbergi* in model D), than the development of a fused LTB in *T. zhengi* could indeed represent a functional advantage, as previously suggested^10^.

Model D (replicating the LTB as seen in *P. sternbergi*), has increased stress and strain values relative to model B, in the same areas as models A and C. Differently from C, however, model D does not have any areas of significant reduction of stress in comparison to model A. In fact, there is a slight increase in stress in the skull roof. In the main areas under stress, Von Mises values on the quadrate are 351 Mpa (8.547% higher than model B, and ca. 3% higher than model A). In the pterygoid, there was a slight decrease of stress, but values are still similar to model A (273 Mpa, or 40.659% higher than model B, and ca. 3% less than model A). Generally speaking, model D is very similar in terms of patterns of stress and strain to model A, and with no significant overall difference in stress values in the areas of highest stress. Therefore, model D is not better suited to forceful biting than the two patterns without a LTB.

## Discussion

Once it is acknowledged that the redevelopment of the LTB does not have a clear selective advantage (as discussed above) for *Polyglyphanodon sternbergi*, then a less functionalist explanation should be considered^39^,^40^. It seems further unlikely that the complete LTB would have developed in this species due to a selective functional advantage that would be restricted only to very old individuals, being absent in juveniles and possibly sub-adults. This would account for one of the examples discussed by Gould and Lewontin^39^ regarding non-adaptive morphological structures: excessive variability (e.g. length and connectivity of the LTB throughout ontogeny in *P. sternbergi*) compared with much reduced variability (e.g. a complete LTB since the hatchling stage in *Sphenodon*) when the same general structure assumes a form judged functional on engineering grounds (reduction of stress during forceful biting or proal shearing, in *Sphenodon*). Therefore, at least in *Polyglyphanodon*, the complete LTB fails explicit tests of adaptationist causes for its reacquisition and unlikely explanations must arise in order to maintain the functionalist point of view.

Some factors need to be considered in order to evaluate the possible causes for the re-development of the LTB in *P. sternbergi*. First, despite the much reduced oral food processing in some leaf cropping lizards (including *Iguana* and, most likely in *P. sternbergi*—see Supplementary Discussion), published data from multiple species indicate that most herbivores (and also omnivores), including species with a variety of food processing strategies, consistently have relatively higher bite force when compared to insectivores^28^,^41^,^42^. Therefore, the presence of a complete LTB in *P. sternbergi*, which implies a reduced cross-section area of the MAMESP compared to most squamates^21^,^28^ (also observed in the extant *Sphenodon*^15^), seems inconsistent with a dietary habit that usually requires higher bite forces.

One possible explanation for this apparent paradox could reside in the large size of species like *Iguana* and *Polyglyphanodon*. After a certain critical size, some lizards may have a proportionally smaller adductor musculature due to an absolute increase in body and muscle size, which would already provide enough bite force sufficient to break food items without further investment in energetically expensive muscle tissues^43^. This idea has been tested for *Uromastyx*^43^, and such a correlation was not detected. However, the authors tested for only two species of *Uromastyx*, both of which attain adult sizes much smaller than adult *Iguana* or *Polyglyphanodon* (close to 50% of the skull length of the specimens we studied). It is thus possible that, after a certain size, there is a proportionally smaller increase of the adductor musculature at least in some clades. More sampling of *in vivo* bite forces from adults of larger-sized lizards may further elucidate this problem.

Secondly, another interesting factor in *P. sternbergi* is the extremely developed medial process of the ectopterygoid that, along with the transverse flange of the pterygoid, extends ventrally for quite some distance (as also seen in other borioteiioids, such as *Gilmoreteius, Adamisaurus* and *Erdenetesaurus*, but uncommon to most lizards^32^—and TRS and RN pers. obs.). These structures may have acted as a medial bracing element for the coronoid dorsal process of the mandibles, guiding the jaws for a precise shearing/cropping. The ventral expansion of the ectopterygoids/pterygoid might also have provided a larger area of attachment for the pterygoidal adductor muscle masses (represented by the MPTT in squamates), which would have contributed towards greater adductor power compared to lizards with a reduced MPTT.

Therefore, reduction of relative adductor size, due to a big absolute size of *P. sternbergi*, along with a likely increase in the size of the MPTT, may have enabled sufficient reduction in size of the MAMESP in *P. sternbergi* that allowed the re-development of the LTB without interfering with the insertion of the MAMESP on the lateral side of the lower jaw (as indicated by the position of the adductor crest). Even if other factors currently unknown to us may also have played a role in producing a relatively smaller MAMESP in *P. sternbergi*, reduction in the relative width of that muscle mass was certainly critical for the re-development of the LTB in *P. sternbergi*, and possibly also in *T. zhengi*. Reduction in size (width, insertion area, or both) of the MAMES was thus a likely factor in removing a physical constraint against the complete LTB and allowing its re-development.

In squamates, the positive directional selection for an expanded and laterally inserting posterior portion of the MAMES, which provides greater adductor power^21^ (see above), seems to have created a structural constraint against the development of a complete LTB. In all known squamates, the LTB is virtually non-existent, being always absent or limited to a short and blunt posteroventral process of the jugal, never reaching the degree of development observed within borioteiioids *T. zhengi* and *P. sternbergi*. The observed phenotypic stasis in this feature, despite numerous variations in the surrounding environmental conditions and dietary habits amongst the numerous squamate families, indicates a case of morphological canalization^44^.

All the factors presented above indicate why the reacquisition of a complete LTB within squamates is such a rare event, further illustrating the unexpectedness of observing independent reacquisitions of this trait within borioteiioids lizards. Evolutionary homoplasies (convergences, parallelisms and reversals) are usually expected to represent similar structural morphologies, functions, or behaviours, as a consequence of different species living under similar conditions^45^, thus being strong evidence of adaptative evolution^2^,^3^. Examples include similar feeding habits^46^ or body shape in aquatic vertebrates^47^. In other cases, convergent evolution may occur due to similar constraints, rather than adaptations, such as the ones driven by structural or phylogenetic constraints—e.g. similar sequences of digit loss in salamanders^4^. However, the convergent evolution of a complete LTB in squamates as represented by *Polyglyphanodon* and *Tyaniusaurus*, is an unexpected example of neither. Their similar morphologies are not convergent solutions to the same pressures, as the LTB in *P. sternbergi* is not adapted for reducing mechanical stress during hard biting, as it might be the case for *T. zhengi*. In fact, the empirical evidence indicates the complete LTB in *P. sternbergi* is not a functional adaptation at all, but might be the result of a release of constraint induced by a reduction in width of part of the adductor musculature. Therefore, while the re-acquisition of the LTB in *T. zhengi* would represent the result of adaptative evolution^10^, in *P. sternbergi* it seems to be the result of constraint release, thus making a case of “mixed” convergence. This opposes most reported cases of homoplastic evolution, which are driven by either similar functionalist or similar structuralist causes in distinct lineages—e.g.^46^,^47^,^48^.

The question remains on how borioteiioids were able to break the constraint observed in squamates against a complete LTB, and what factors may have driven it more than once. It is not possible to assess whether the homoplasy of this particular feature is the result of reoccurring genetic mutation in either species^49^, or the unmasking (“de-canalization”) of hidden cryptic genetic variation^50^, as borioteiioids form an entirely extinct clade of lizards. However, the fact that amongst all living and fossil squamates—extant forms alone representing over 9,000 species^51^—the only two taxa in which a complete LTB is known to have developed belong to the same, relatively small clade (in terms of species richness), may favour the latter hypothesis of “de-canalization”. *Polyglyphanodon* and *Tyaniusaurus* indicate that the genetic and developmental framework necessary for the production of a complete LTB was present in geographically and phylogenetically distinct sub-groups of borioteiioids, and the same potentiality does not occur in other clades of squamates. If reoccurring mutations were enough to redevelop a complete LTB when favourable conditions occurred (i.e., in other hard biting lizards, such as *Uromastyx*), then it would be expected to find that variety in other clades as well. Taxa that are more closely related to each other have more similar genetic and developmental backgrounds due to phylogenetic constraints, and thus should have a more similar range of phenotypic responses to variations in surrounding conditions as compared to more distantly related species. Therefore, we conclude that their convergent evolution may actually reflect a genetic/developmental predisposition, allowing for the re-development of a complete LTB. That predisposition seems to have been reacquired at some point in the early evolution of borioteiioid lizards, but does not seem to have developed (or reacquired) in other squamate clades.

## Methods

### Referred specimens

*Polyglyphanodon sternbergi*: NMNH (National Museum of Natural History – Smithsonian Institute) 15477 (holotype); 15816 (paratype); 15559; 15566; 15567; 15568; 15573; 15817; 15818; 15819; 16367; 16368; 16369; 16374; 16584; 16585; 16586; 16587; 16588; 16724; 427672; 427678; 427682; 427683; 427777 and CM (Carnegie Museum of Natural History) 9188. *Tupinambis teguixin*: FMNH (Field Museum of Natural History) 140193, TMP (Royal Tyrrell Museum) 1990.007.0352; *Iguana iguana* UAMZ (University of Alberta Museum of Zoology) uncatalogued frozen specimen; CM 38489; CM 114409; 92303; CM 125934; *Agama agama* (Midwestern University uncatalogued).

### Measurements

Skull height (SH), straight line in lateral aspect from contact of prefrontal with frontal dorsally, to dentigerous border of maxilla ventrally; skull width (SW), distance between lateral border of both postorbitals as seen in dorsal aspect; skull length 1 (SL1), anterior tip of premaxilla to posterior border of parietal table; skull length 2 (SL2), anterior tip of premaxilla to occipital condyle; parietal width (PW), straight line between parietal suture with postfrontals in dorsal aspect; parietal body length (PBL), midline of fronto-parietal suture to midline of posterior border of parietal in dorsal aspect.

Different measurements (e.g. SL1 and SL2) were taken in order to have a larger amount of comparable data across specimens that have differential degrees of preservation (e.g. the occipital condyle region is not preserved in NMNH 15477, so only SL1 allows some measure of skull length for that specimen). When the skull suffered some deformation, but one of the sides still had the elements in articulation, only that side was measured (e.g. only the left side in 16588 for SW), and the obtained value was doubled in order to estimate the value for the opposite side. Because the lower jaws were usually disarticulated from the skull, and occasionally laterally displaced, head height (including the lower jaws) was not measured, but skull height was used instead (see above).

### Phylogenetic analysis

We investigated the phylogenetic position of *Polyglyphanodon sternbergi* using an existing matrix^52^, plus the addition of other borioteiioids from east Asia. These include *Aprisaurus, Tuberocephalosaurus*, an unnamed taxon from Jiangxi (Jiangxi_2), and published scorings for *Tianyusaurus*^32^. Other character scorings were corrected for some other borioteiioid taxa (*Erdenetesaurus*, *Adamisaurus*, *Cherminsaurus*, *Gobinatus*, *Darchansaurus*, *Gilmoreteius* and *Chamops*) in the present matrix, but not to the same extent as performed for *P. sternbergi* (scorings available in Supplementary Data). Also, one character state was rescored for Jiangxi_2 (character 96) and two were rescored for *Aprisaurus* (characters 30 and 96 were scored A and B, which we considered to be typeset errors and scored “?” for both).

These changes resulted in matrix of 229 taxa and 363 characters that was run using the software T.N.T., with “Rhynchocephalia” designated as the outgroup. The new interpretation for the morphology of *P. sternbergi* along with data that was not originally scored^52^ resulted in 83 characters out of the 363 in total that were modified by us, representing a change in 27% of all the osteological characters of *P. sternbergi* (and 22% overall) in this matrix. In all the analyses performed, searches were initially run using the ‘New Technology Search’ options followed by a “Traditional Search” following the protocol in^53^.

### Model choice

Despite the existence of relatively complete skulls of *P. sternbergi*, their distortion and lack of a fully intact skull makes the usage of a modern analogue a better structural candidate for a biomechanical assessment. We used the skull of *Iguana iguana* as a proxy because it has skull dimensions for adults that are very similar to adults of *P. sternbergi*, both in width/length proportions, as well as absolute length. Furthermore, *Iguana iguana* has a naturally non-streptostylic quadrate, which is also the case for *P. sternbergi*. We utilized the skeletally mature skull of an adult *I. iguana* (UAMZ uncatalogued, obtained from the pet trade), with SL1 = 70.79 mm; SW = 41.69 mm. The specimen was kept frozen since its death, which reduces the chances of having bone articulations being modified, such as being drawn closer together, due to dehydration or physical removal of soft tissue during dry skeleton preparations.

### *Ex vivo* Micro-Computed Tomography

The skeletally mature skull of an adult *Iguana iguana* was secured in the gantry bed of a Skyscan 1076 micro-Computed Tomograph (Bruker-Skyscan, Kontich, BE). The sample was scanned in entirety at 35 μm pixel size, using 6 overlapping unit sub-scan scan lengths, with tube voltage at 100 kV, and a current of 100 μA. Low energy X-rays were removed using a 0.5 mm aluminum filter, and three scan projections were averaged per step, through 180° of rotation at 0.7° step increments with 474 ms exposure time. Using a modified Feldkamp back-projection algorithm, the raw image data were reconstructed at a cross-sectional threshold of 0.0–0.04 using NRecon reconstruction software (version 1.4.4, Skyscan NV, Belgium).

### Mesh creation and properties

Reconstructed micro-CT scan data were imported in bitmap format into Mimics x64 and thresholded to produce a surface mesh. The surface mesh was cleaned and repaired in Mimics x64 and Geomagic Studio 12. The cleaned mesh was modified in Geomagic Studio 12 to produce two hypothetical models: one with a complete lower temporal bar, and one with an incomplete lower temporal bar attached by a ligament to the quadrate. The ligaments in all models were represented by sets of tension-only springs with a total stiffness of 50 N/mm^10^. Number of finite elements (tetrahedral solid): 1,427,173 (models A and B); 1,443,351 (model C); and 1,466,414 (model D). Bone was modeled as an isotropic material, with Young’s modulus = 10Gpa, and Poisson’s ratio = 0.4^54^,^55^,^56^. Biting force was applied at an angle of 90° to the tooth row, as would be expected in *P. sternbergi* given the precise tooth interdigitation observed in that species.

### Muscle forces

Muscle forces for FEA analyses which aim to test stress levels on rigid bodies are dependent on a series of distinct variables, such as each muscles’ physiological cross-section, fiber length and gape angle^57^,^58^, which cannot be precisely estimated from fossil species. Therefore, a range of force input values was used to test for possible variations, especially due to head dimensions. Data from published values for the extant lizard species *Uromastyx hardwickii* were used^10^, and scaled to the skull length of *Iguana iguana* , which is equivalent to *P. sternbergi* (Supplementary Table S1). *Uromastyx hardwickii*, is a herbivore that displays hard-biting, which is the anatomical/ecological model to be tested^10^. Force values are expected to scale to the square of linear measurements of the body (slope = 2.0) if both grow isometrically to each other^59^. However, data collected by Herrel^42^ indicates positive allometry for muscle forces against skull length among different species of lizards: for herbivorous adult males, bite force vs. head length (measured equally to our SL1 values for *I. iguana* and *P. sternbergi*) slope = 2.7489. We therefore used the latter slope to calculate the scaled forces. The same forces were then scaled again for subsequent analyses (2× and 4× the initial scaled values) to observe if variations in muscle force values would affect the result of stress level.

### Application of FEA

Origins of twenty four adductor muscles were mapped on the meshed geometry of all models (using Geomagic Studio 12), and finite element nodes corresponding to each muscle origin were marked using published data on the myology of *Iguana iguana*^31^,^36^ and from our own dissections. The load of each muscle was then evenly distributed over the nodes corresponding to each muscle origin. This provided a more precise application of muscle loads and stress distribution on areas of attachment than previous applications that used only single or a few nodes for the application of muscle loading conditions^10^,^60^, which created an excessive concentration of stress around those nodes.

Bite forces were applied—one per side—on nodes posteriorly on the tooth row (fifth tooth from posteriormost tooth). This is the position where *P. sternbergi* and *Iguana iguana* have specialized teeth for cropping, and where bite forces are greater along the tooth row^60^. Because forces were scaled from a previous study^10^, rather than derived *de novo* in an MDA, equilibrating joint forces from the same studies were inapplicable. Therefore, to ensure equilibrium in the system, the mandibular condyles of the quadrate were fixed, to approximate the near-frictionless joint with the mandible and account for resulting joint forces. Forces, directions, and node selection were controlled between all models. Each of the three skull models—unmodified, complete lower temporal bar, and incomplete temporal bar—was tested identically using the same muscular origins and insertions. Preparation of the FE models, i.e meshing and applying boundary conditions and forces, were performed using Hypermesh v13.0. Linear static finite element analyses were then performed on each model using ABAQUS v6.1.12.

## Additional Information

**How to cite this article**: Simões, T. R. *et al*. Reacquisition of the lower temporal bar in sexually dimorphic fossil lizards provides a rare case of convergent evolution. *Sci. Rep*. **6**, 24087; doi: 10.1038/srep24087 (2016).

## Supplementary Material

Supplementary Information

## Figures and Tables

**Table 1:**
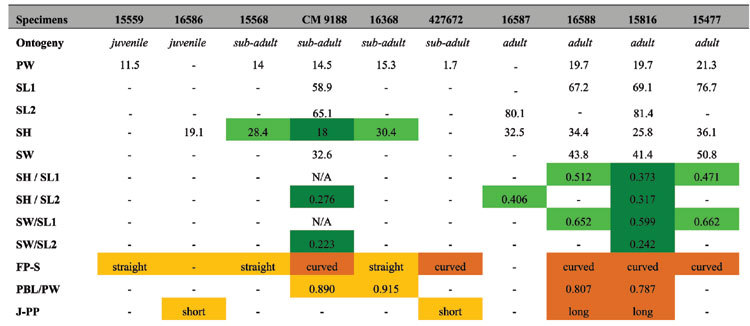
Measurements obtained from different *Polyglyphanodon sternbergi* specimens.

Color legend: Light green: morphotype A, dark green: morphotype B; yellow: juvenile features, orange: sub-adult/adult features; dashes, missing data. Abbreviations: FP-S, fronto-parietal suture shape; J-PP, jugal, posterior process length; PBL, parietal body length; PW, parietal width; SH, skull height; SL1, skull length 1; SL2, skull length 2; SW, skull width.
