# Supplementary material for: Reacquisition of the lower temporal bar in sexually dimorphic fossil lizards provides a rare case of convergent evolution
Source: Sci Rep. 2016 Apr 13;6:24087. doi: 10.1038/srep24087 (PMC4829860; doi:10.1038/srep24087)
Supplement: Supplementary Information [file srep24087-s1.pdf]

Reacquisition of the lower temporal bar in sexually dimorphic fossil lizards provides a rare case of convergent evolution

Tiago R. Simões, Gregory Funston, Behzad Vafaeian, Randall L. Nydam, Michael R. Doschak & Michael W. Caldwell

Supplementary Information

1. Supplementary Figures

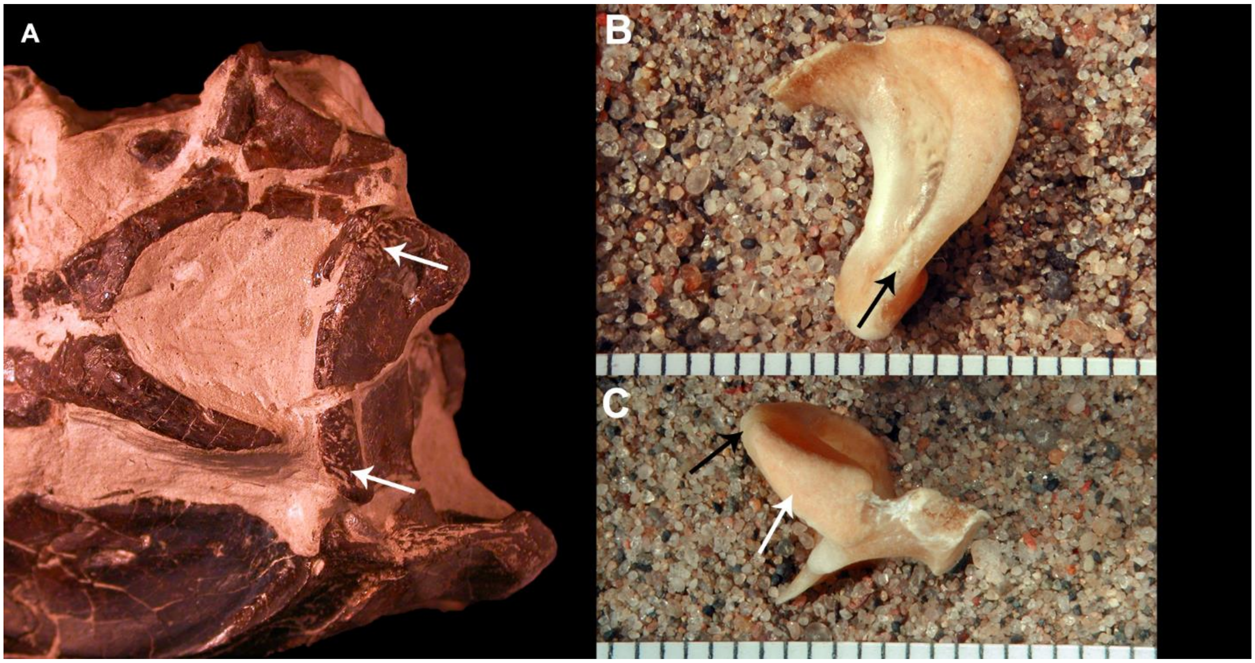

Figure S1. (**A**), quadrate of *P. sternbergi* (NMNH 16587); (**B** & **C**), quadrate of *Tupinambis teguixin* FMNH 140193. White arrows indicate regions of soft tissue contact with the quadrate (rugose texture on the surface of the bone), and black arrows indicate regions in which the quadrate is smooth. Despite the excellent preservation of the texture of the tympanic crest in NMNH 16587, the area of contact with the temporal elements is not well preserved (many parts are broken, and the most critical ones are embedded in matrix). See Supplementary Fig. 4 (below) for the morphology of the temporal region based on the specimen in which that region is best preserved.

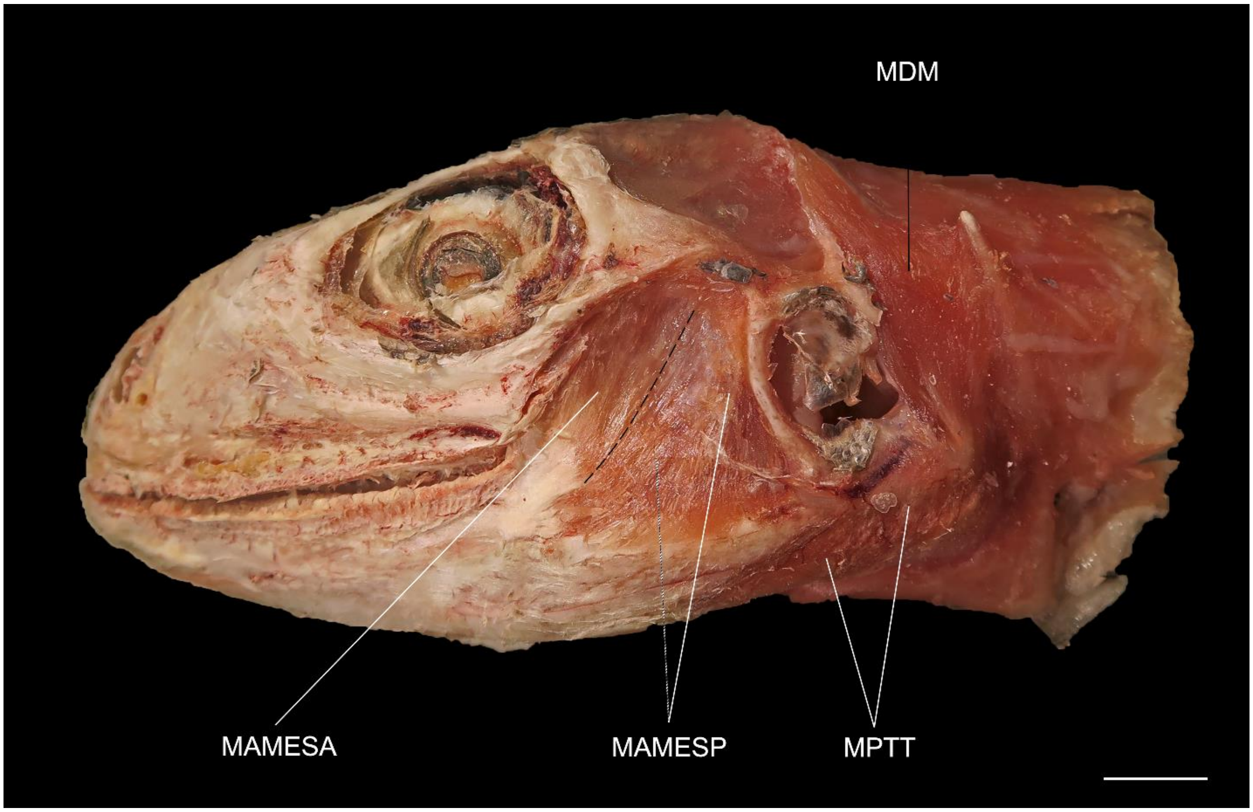

Figure S2. Superficial layer of skull muscles of *Iguana iguana* (UAMZ uncatalogued). The MAMESP extends laterally and over the lower jaw posteriorly, expanding the anteroposterior extent of the MAMES in comparison to *Sphenodon*[1-3]. The anterior portion of the same muscle (MAMESA), also attaches to the lateral surface of the lower jaw, just posterior to the coronoid bone, but does not extend as far ventrally as the MAMESP. Abbreviations: MAMESA, *Musculus adductor mandibulae externus superficialis anterior*; MAMESP, *Musculus adductor mandibulae externus superficialis posterior*; MDM, *Musculus depressor mandibulae*;; MPTT, *Musculus pterygoideus typicus*. Scale bar = 10mm.

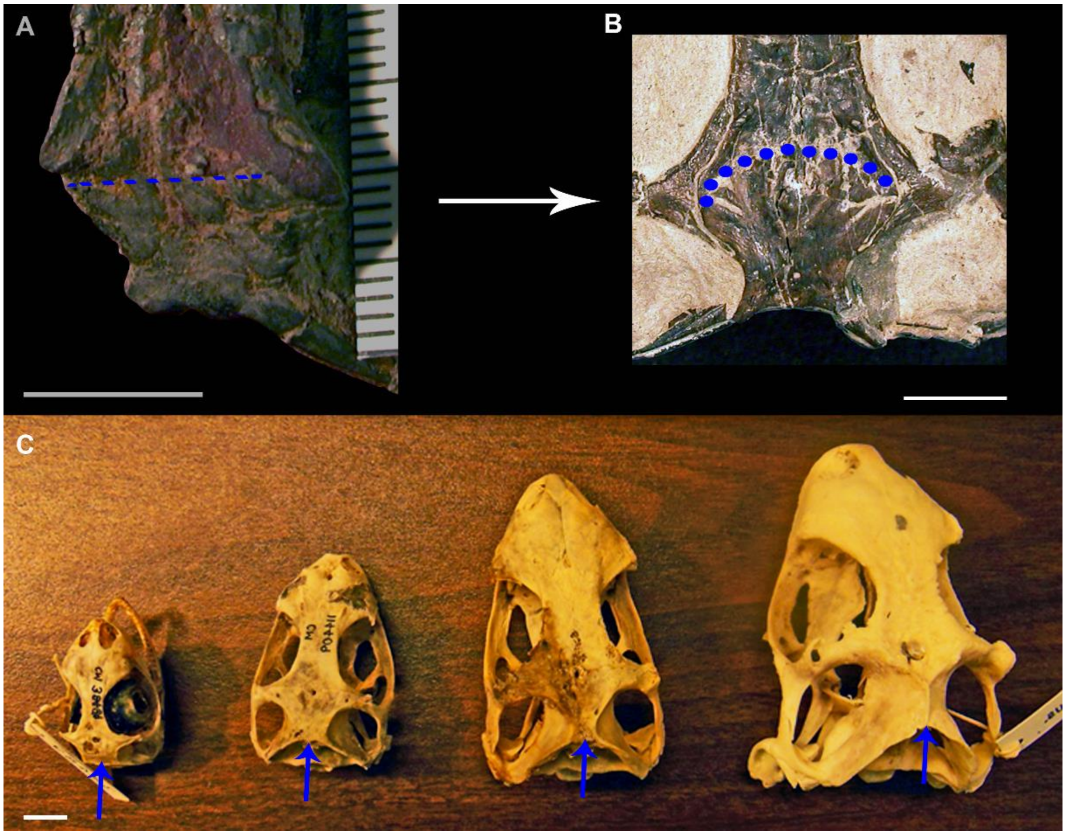

Figure S3. (**A**), the almost straight frontoparietal suture of a juvenile of *P. sternbergi* (NMNH 15568) and (**B**) the anteriorly curved condition in the adults (NMNH 16588). Dramatic changes in the shape of the parietal during ontogeny in the extant *Iguana iguana*. Scale bars equal to 10mm.

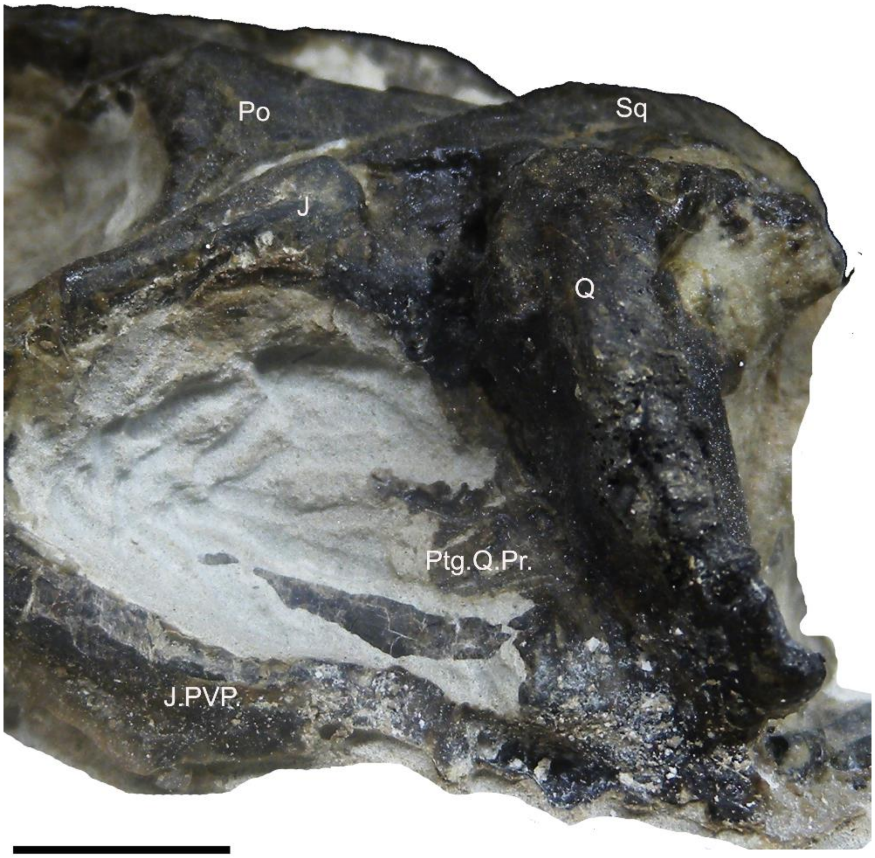

Figure S4. Temporal region of *P. sternbergi* (NMNH 15816). Note the increased area of anterodorsal contact between the quadrate, jugal and squamosal, as well as the quadrate process of the pterygoid ventrally. Abbreviations: J, jugal; J.PVP, posteroventral process of the jugal; Po, postorbital; Ptg.Q.Pr.; quadrate process of the pterygoid; Q, quadrtae; Sq, squamosal. Scale bar = 10mm.

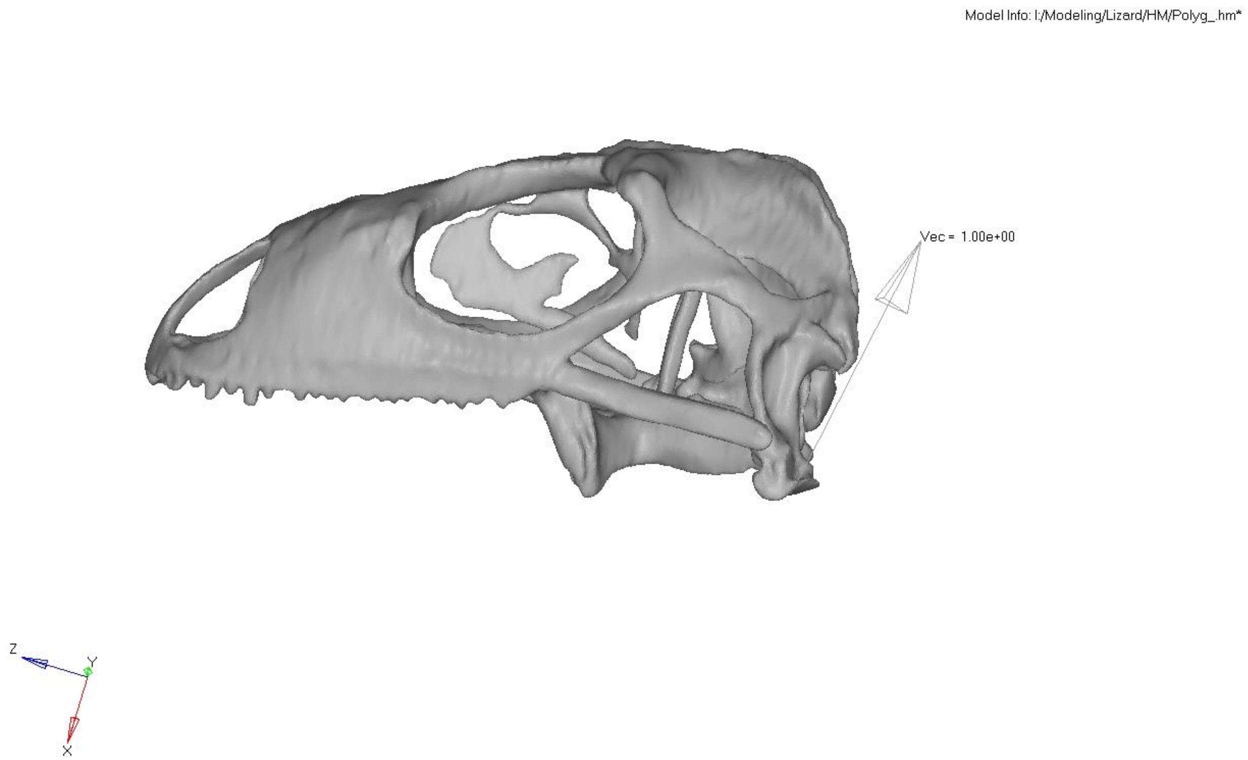

Figure S5. Lateral view of finite element analysis results for our hypothetical model D, indicating the direction of the resultant joint reaction force at the quadrato-mandibular joint during biting. In all models, the forces were directed posterodorsally, resulting in a tendency for the quadrate to rotate posteriorly, keeping ligaments or the LTB under tension. For specific direction and magnitudes of joint reaction force at the quadrato-mandibular joint see Supplementary Table S4 (below).

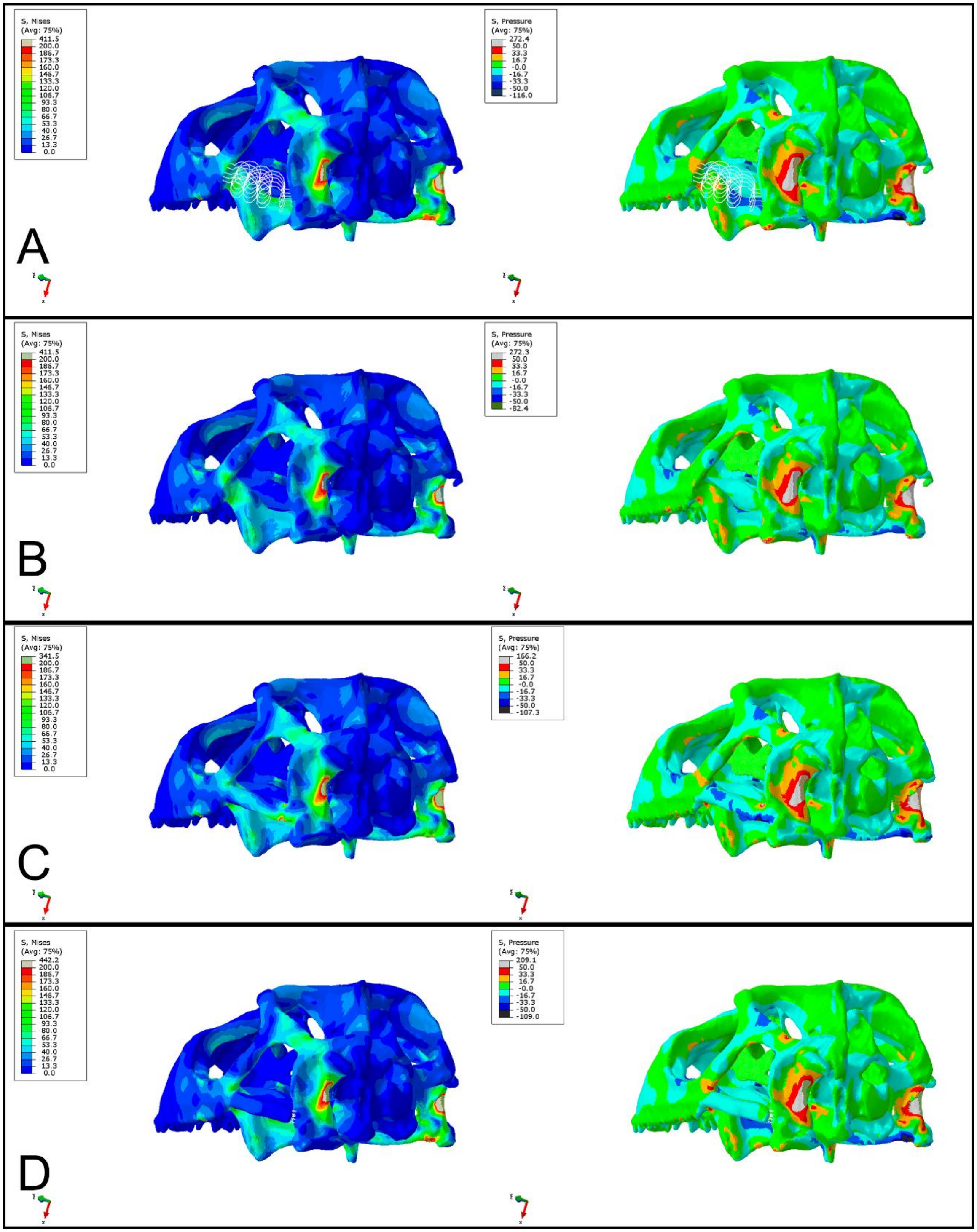

Figure S6. Posterolateral view of Finite Element analysis results for four hypothetical skull models (see main text). Contours indicate von Mises stress (warmer colours are higher stress), left, and pressure (cold colours are tension, warm colours are compression), right.

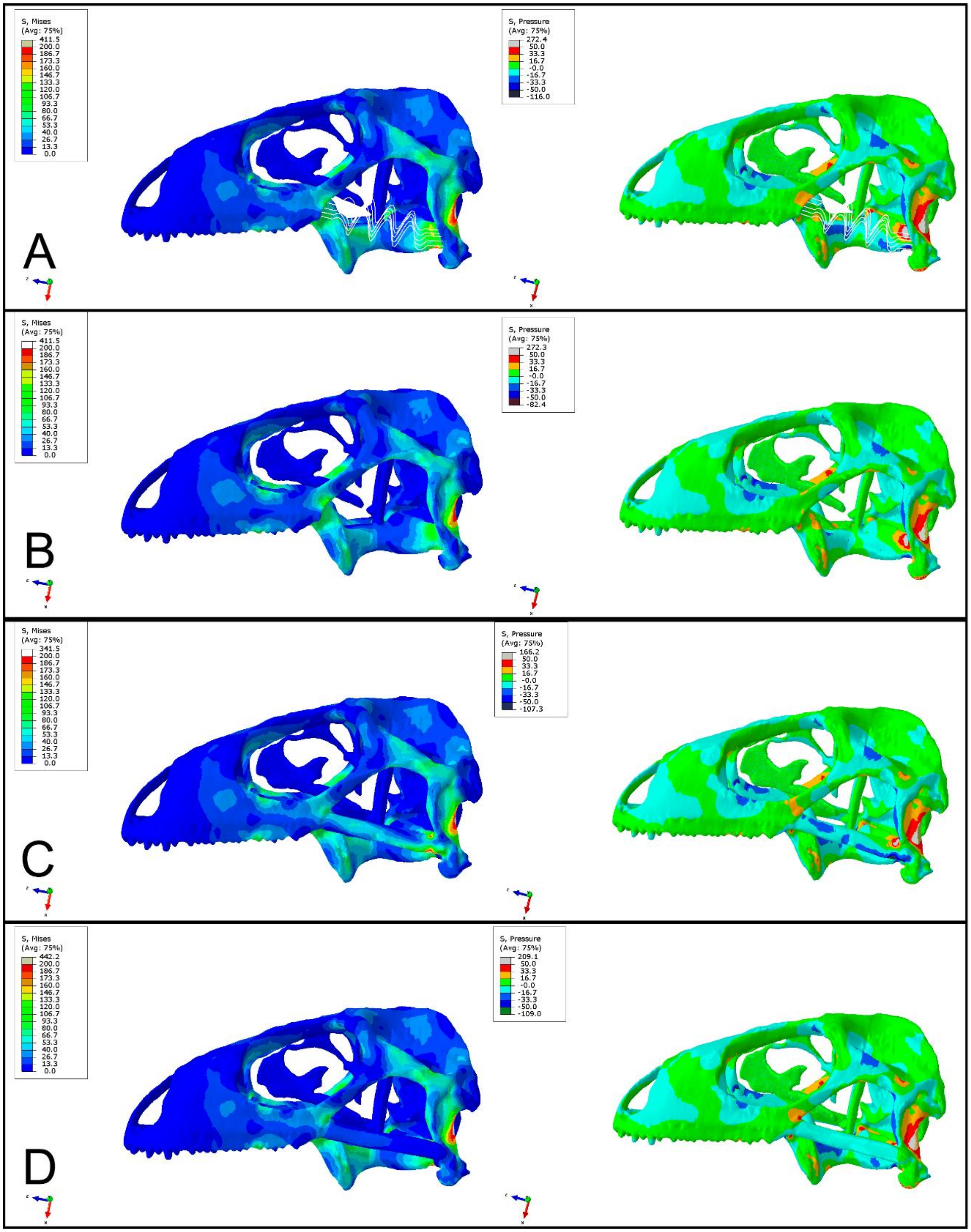

Figure S7. Lateral view of Finite Element analysis results for four hypothetical skull models (see main text). Contours indicate von Mises stress (warmer colours are higher stress), left, and pressure (cold colours are tension, warm colours are compression), right.

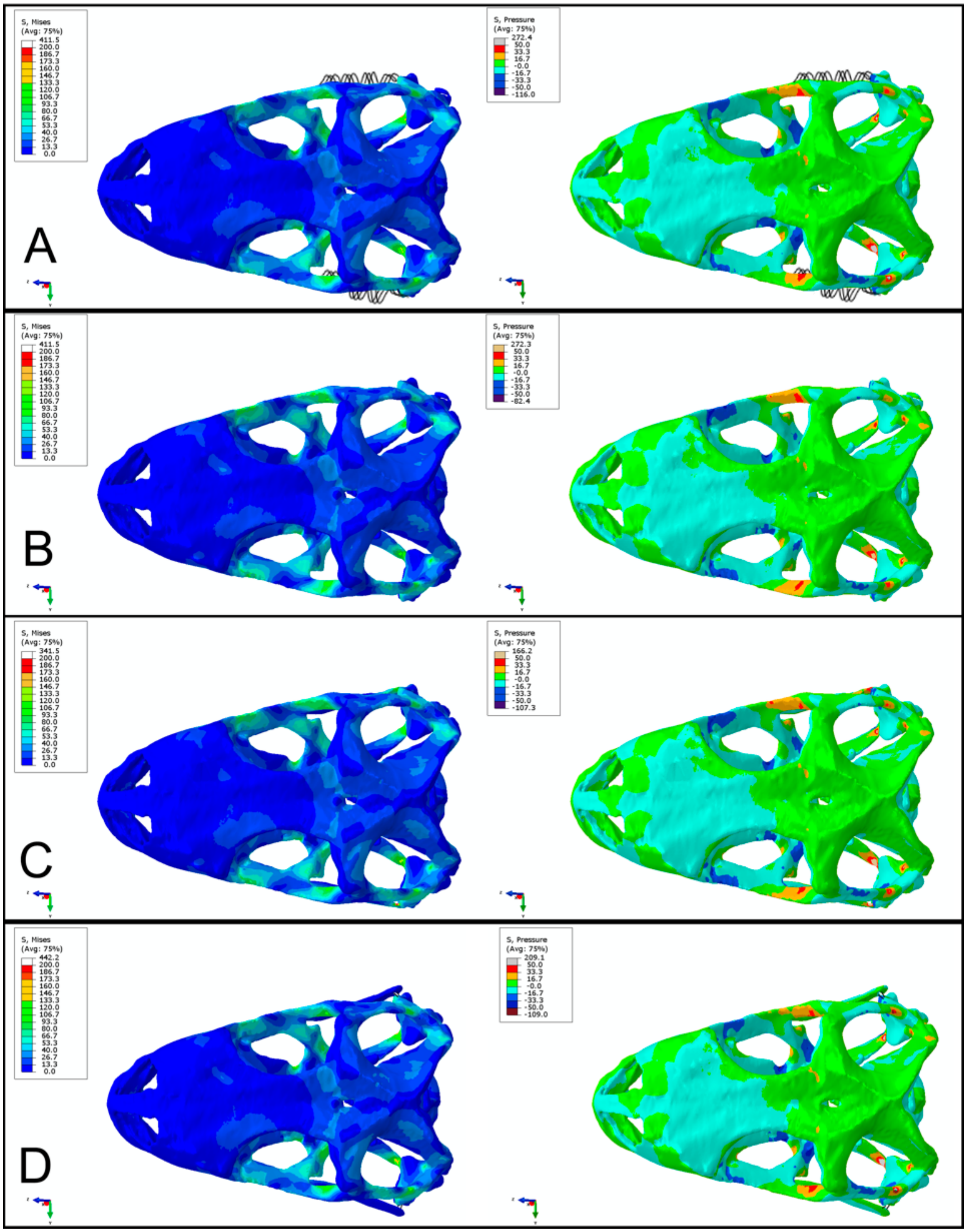

Figure S8. Dorsal view of Finite Element analysis results for four hypothetical skull models (see main text). Contours indicate von Mises stress (warmer colours are higher stress), left, and pressure (cold colours are tension, warm colours are compression), right.

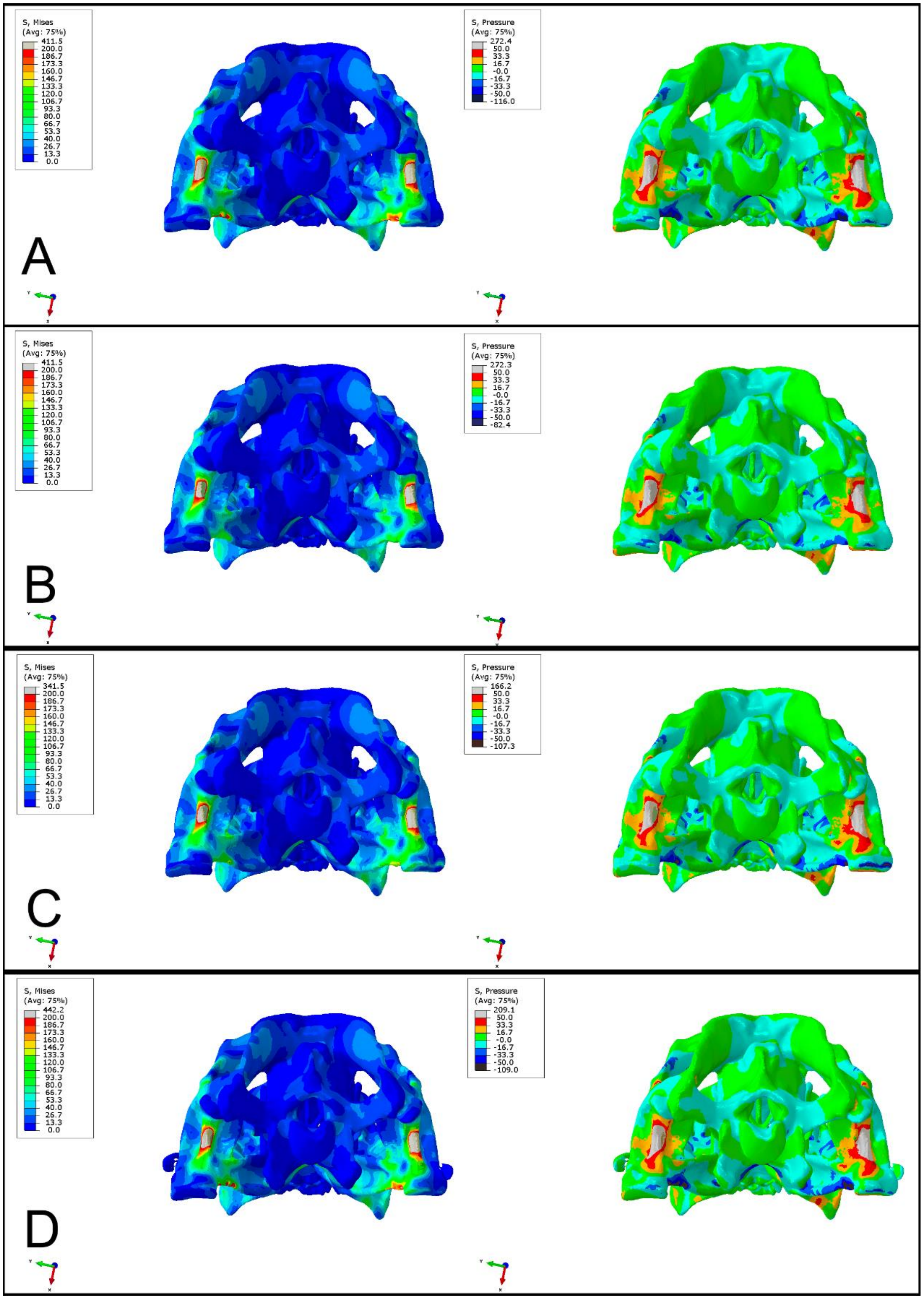

Figure S9. Posterior view of Finite Element analysis results for four hypothetical skull models (see main text). Contours indicate von Mises stress (warmer colours are higher stress), left, and pressure (cold colours are tension, warm colours are compression), right.

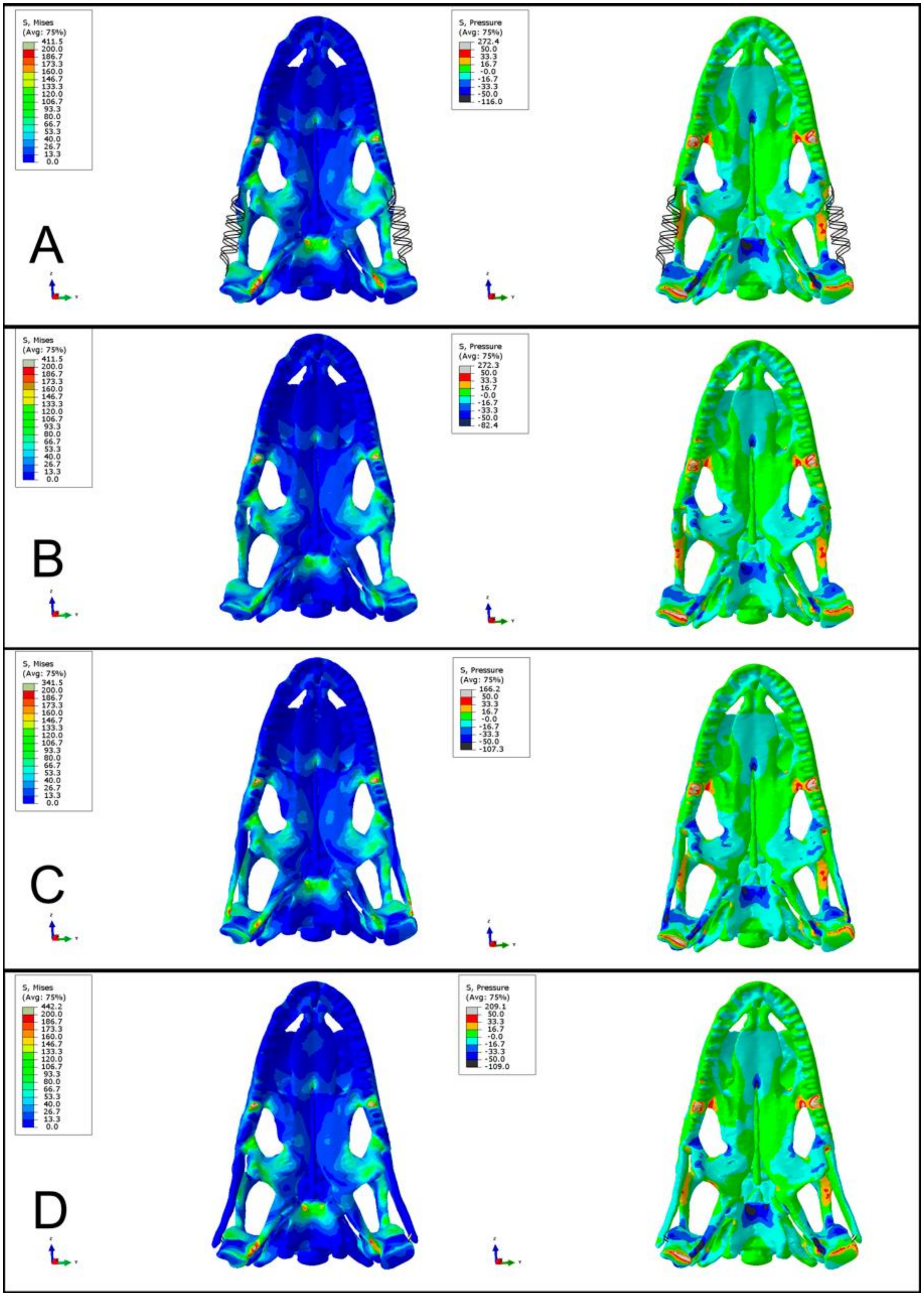

Figure S10. Ventral view of Finite Element analysis results for four hypothetical skull models (see main text). Contours indicate von Mises stress (warmer colours are higher stress), left, and pressure (cold colours are tension, warm colours are compression), right.

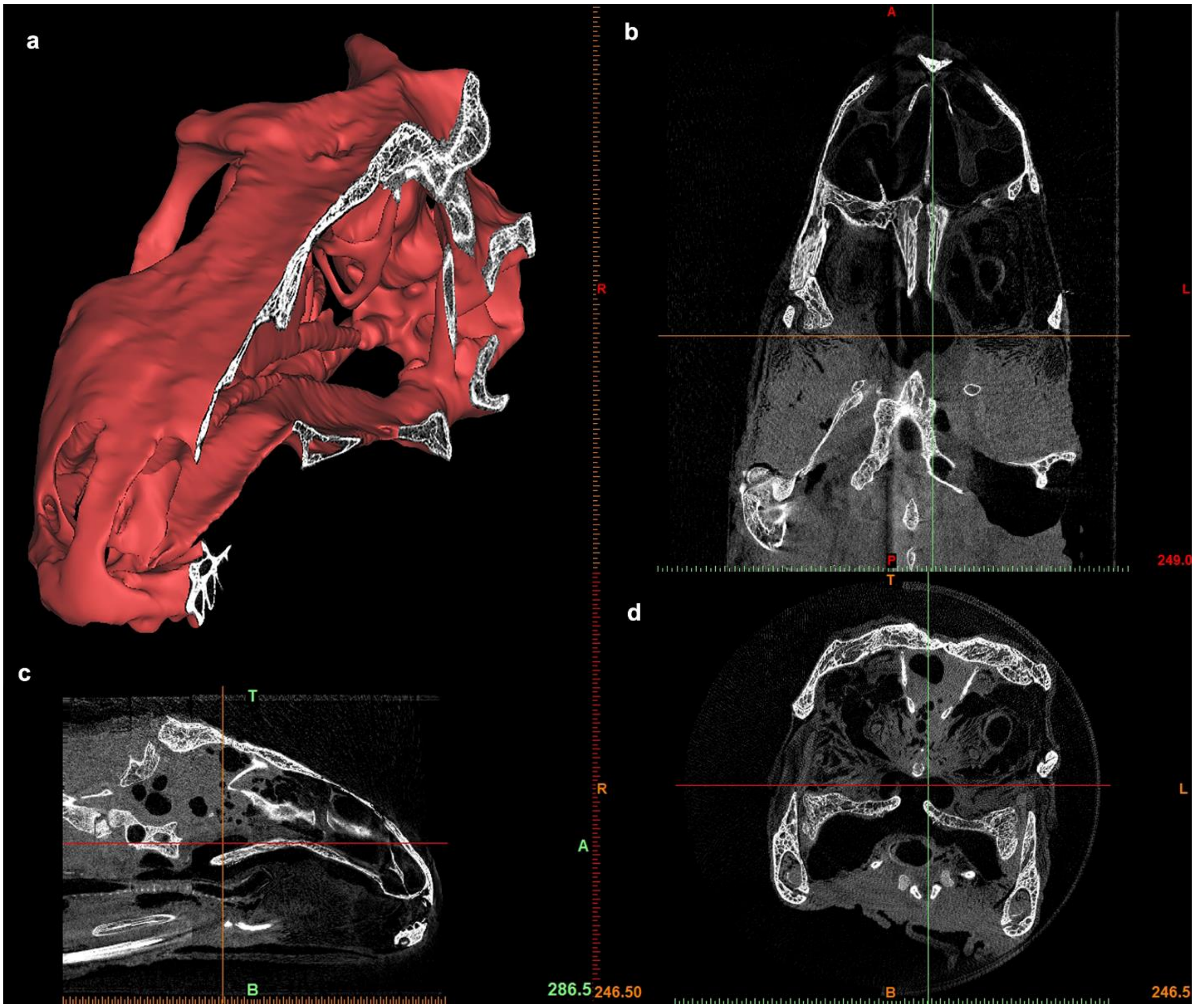

Figure S11. CT scan data of *Iguana iguana*. (A) 3D reconstruction with sagittal cut. (B) slice of axial view. (C) slice of sagittal view. (D) slice of coronal view.

2. Supplementary Table

Table S1. Muscle directions and loads used in the FEA.

	Muscle Direction			Load Case 1			
**Muscle Group**	**X**	**Y**	**Z**	**Scaled force**	**X**	**Y**	**Z**
**R MAMP**	0.867	-0.342	0.363	23.405	20.293	-7.999	8.487
**L MAMP**	0.941	0.163	0.297	23.405	22.021	3.805	6.959
**R MPTT 1**	-0.095	-0.175	-0.980	31.304	-2.959	-5.474	-30.680
**L MPTT 1**	0.334	0.828	-0.450	31.304	10.464	25.921	-14.091
**R MPTT 2**	-0.009	-0.328	-0.945	29.011	-0.265	-9.519	-27.404
**L MPTT 2**	0.021	0.337	-0.941	29.011	0.619	9.775	-27.308
**R MPTT 3**	0.617	-0.761	-0.198	26.973	16.651	-20.539	-5.333
**L MPTT 3**	0.766	0.613	-0.193	26.973	20.669	16.533	-5.195
**R MAMIPS**	0.828	-0.505	0.245	4.987	4.128	-2.517	1.220
**L MAMIPS**	0.935	0.266	0.235	4.987	4.661	1.328	1.174
**R MAMIPP**	0.802	-0.596	-0.034	4.805	3.853	-2.865	-0.165
**L MAMIPP**	0.935	0.331	-0.123	4.805	4.495	1.592	-0.593
**R MAMESA**	0.961	-0.271	0.059	44.882	43.116	-12.184	2.629
**L MAMESA**	0.872	0.490	0.014	44.882	39.120	21.990	0.643
**R MAMESP**	0.890	-0.168	0.425	47.684	42.419	-8.017	20.253
**L MAMESP**	0.850	-0.122	0.512	47.684	40.531	-5.830	24.435
**R MAMEM 1**	0.829	-0.405	0.385	33.889	28.106	-13.708	13.060
**L MAMEM 1**	0.889	0.161	0.428	33.889	30.131	5.462	14.517
**R MAMEM 2**	0.835	-0.492	0.247	33.670	28.100	-16.578	8.322
**L MAMEM 2**	0.934	0.236	0.270	33.670	31.432	7.945	9.088
**R MAMEP 1**	0.789	-0.508	0.346	17.399	13.724	-8.838	6.023
**L MAMEP 1**	0.902	0.298	0.314	17.399	15.689	5.180	5.457
**R MAMEP 2**	0.703	-0.355	0.617	17.399	12.224	-6.177	10.731
**L MAMEP 2**	0.824	0.231	0.517	17.399	14.339	4.025	8.997
**Ligament 1**	0.186	-0.285	-0.940	21.076	3.924	-6.010	-19.816
**Ligament 2**	0.419	-0.320	-0.850	7.608	3.184	-2.433	-6.467
**Bite Force**	-0.981	0.146	0.126	79.573	-78.079	11.590	10.060

Table S2. Muscle directions and loads used in the FEA.

	Load Case 2				Load Case 3			
**Muscle Group**	**Scaled force**	**X**	**Y**	**Z**	**Scaled force**	**X**	**Y**	**Z**
**R MAMP**	46.811	40.586	-15.998	16.973	93.622	81.172	-31.996	33.946
**L MAMP**	46.811	44.041	7.610	13.919	93.622	88.082	15.220	27.837
**R MPTT 1**	62.609	-5.917	-10.949	-61.359	125.217	-11.834	-21.897	-122.718
**L MPTT 1**	62.609	20.928	51.843	-28.182	125.217	41.855	103.686	-56.363
**R MPTT 2**	58.022	-0.531	-19.038	-54.807	116.044	-1.061	-38.076	-109.615
**L MPTT 2**	58.022	1.238	19.549	-54.616	116.044	2.477	39.099	-109.231
**R MPTT 3**	53.945	33.301	-41.078	-10.665	107.891	66.603	-82.155	-21.330
**L MPTT 3**	53.945	41.338	33.065	-10.390	107.891	82.676	66.130	-20.780
**R MAMIPS**	9.974	8.257	-5.035	2.439	19.947	16.514	-10.069	4.879
**L MAMIPS**	9.974	9.323	2.655	2.348	19.947	18.645	5.311	4.696
**R MAMIPP**	9.610	7.707	-5.731	-0.330	19.219	15.414	-11.462	-0.659
**L MAMIPP**	9.610	8.989	3.183	-1.186	19.219	17.978	6.367	-2.372
**R MAMESA**	89.763	86.232	-24.367	5.259	179.527	172.465	-48.735	10.517
**L MAMESA**	89.763	78.241	43.979	1.287	179.527	156.482	87.958	2.574
**R MAMESP**	95.369	84.837	-16.034	40.507	190.738	169.674	-32.068	81.013
**L MAMESP**	95.369	81.062	-11.661	48.869	190.738	162.124	-23.321	97.739
**R MAMEM 1**	67.777	56.212	-27.417	26.121	135.555	112.425	-54.833	52.241
**L MAMEM 1**	67.777	60.262	10.924	29.034	135.555	120.523	21.849	58.068
**R MAMEM 2**	67.341	56.200	-33.155	16.644	134.681	112.401	-66.310	33.288
**L MAMEM 2**	67.341	62.865	15.889	18.175	134.681	125.729	31.778	36.350
**R MAMEP 1**	34.799	27.449	-17.676	12.045	69.597	54.897	-35.352	24.090
**L MAMEP 1**	34.799	31.377	10.359	10.913	69.597	62.755	20.718	21.826
**R MAMEP 2**	34.799	24.449	-12.353	21.462	69.597	48.897	-24.706	42.924
**L MAMEP 2**	34.799	28.677	8.050	17.994	69.597	57.355	16.099	35.987
**Ligament 1**	42.152	7.848	-12.021	-39.632	84.304	15.696	-24.042	-79.264
**Ligament 2**	15.216	6.368	-4.865	-12.934	30.432	12.737	-9.731	-25.869
**Bite Force**	159.506	-156.511	23.232	20.166	319.012	-313.023	46.464	40.332

Table S3. Combined joint reaction forces at quadrato-mandibular joint

Model	Fx	Fy	Fz	Magnitude (N)
**A**	-1150	-26.2	-205.6	1166.7
**B**	-1150	-26.5	-206.2	1168.6
**C**	-1148	-26.2	-205.6	1166.55
**D**	-1206	-41.1	4.09	1206.7

Force magnitudes represent the combined joint reaction forces of both sides of the skull. The component magnitude for each individual joint was approximately half of the total magnitude, with less than 2.5% variation between each side.

Table S4. Von Mises stress and compression values at areas of highest stress in the skull.

Model	Von Mises at Q (Mpa)	Ratio against model B (%)	Von Mises at Ptg (Mpa)	Ratio against model B (%)
**A**	340	+5.6	285	+43.2
**B**	321	0	162	0
**C**	341	+5.9	225	+28
**D**	351	+8.5	273	+40.7

Abbreviation: Q, quadrate; Ptg, pterygoid. These were the areas with highest von Mises stress and compression values other than the tooth upon which bite forces were applied. Values are of the element's centroid (integration point), which may be slightly higher than the extremes of the contour legends; the values of the contours in von Mises and pressure maps are average values of the elements in each region. The software average the values to plot a continuous contour map (color).

3. Supplementary Discussion

Sexual dimorphism

In the observed specimens of *Polyglyphanodon sternbergi* there are two different skull morphotypes: one represented by proportionally taller skulls (Figs. 1b,j), and the other by proportionally more depressed skulls (Figs. 1i,k), when height is compared against skull length. The snout-vent length is commonly used as an independent variable to determine whether these differences among morphotypes are due to changes in relative height or another dimension of the skulls (width or length). However, considering there are few and mostly disarticulated postcranial materials associated with the skulls, it is difficult to determine the snout-vent length in most of the available specimens. Yet, specimens NMNH 16588 and NMNH 15816 have skulls of fairly similar length and width, but specimen NMNH 16588 is taller than NMNH 15816 (see Table 1), indicating these morphotypes differ mostly in relative height. Skull height vs. length ratios for specimens NMNH 16588 and 15477 indicate they are more similar in relative skull height to each other (morphotype A) than to the more depressed skull condition observed in NMNH 15816 and CM 9188 (morphotype B). Despite NMNH 16587 not being directly comparable to NMNH 16588 and NMNH 15477 using the measured data, the SH/SL2 ratio indicates this specimen also has a much taller skull in relation to NMNH 15816 and CM 9188, thus belonging to morphotype A. Somewhat larger skulls are also observed in morphotype A in relation to morphotype B, but not to the same extent as the difference in height. Furthermore, CM 9188 has a slightly longer skull profile in relation to NMNH 15816, but the former is a juvenile and it probably attained a somewhat proportionally shorter skull with age, as indicated by the SW/SL2 and SH/SL2 ratios.

Sexual dimorphism in lizards commonly affects body proportions. For instance, females tend to have longer interlimb lengths, which is usually associated with providing greater fertility (more space for a bigger clutch)[4,5]. Males, on the other hand, usually have bigger heads due to male-male combat for territory, male-female interaction during mating, or different food niche partitioning between males and females [4,6-10].

Changes in relative size of the head in male lizards may also be followed by changes in shape, such as when variations in head length, width, and height dimensions are allometric. For instance, males of *Gallotia galloti* have greater relative increase in the length of their skulls, creating greater gape size and a proportional increase in the length (and power) of the MPTT used for male-male combat [7]. Males may also have relatively wider skulls, as in *Gymnophtalmus multicustatus* [9] and both wider and longer in *Cnemidophorus ocellifer* [11]. Taller skulls occur in males of different species of *Podarcis* [12] and *Dinarolacerta* [5]. Relatively taller skulls are advantageous for male herbivorous lizards that engage on male-male combat or that use the jaws to hold females during copulation. Food niche partitioning between both sexes could also be a possibility, but this is usually restricted to insectivorous lizards [10]. Following this reasoning, it is suggested that morphotype A, with taller and, to a lesser degree, wider skulls, might represent males of *P. sternbergi*, whereas morphotype B represents females. It is plausible that, as in extant lizards from

different families, males suffered natural or sexual selection for a taller and wider skull due to dispute for females, or territories.

Ontogeny

The ontogenetic status of the studied specimens is based on both the relative size among the many available materials (see Table 1), and ontogenetic markers for post-embryonic development of extant squamates. The latter markers include: full ossification of mesopodial elements; fusion of elements of the pelvic girdle; fusion of neurocentral sutures; a great degree of ossification of dermal skull bones; and great development of the parietal supratemporal processes, which are small during early ontogenetic stages[13-16]. Most importantly, the fusion of humeral and femoral proximal epiphyses (as seen in the holotype and the paratype, NMNH 15477 and NMNH 15816, for instance) indicate that NMNH 15477 and NMNH 15816 had reached skeletal maturity, which in many extant squamates occurs only very late during ontogeny, and after sexual maturity in many instances[15].

There are important morphological differences between adult-sized specimens of *P. sternbergi* and smaller (and younger) ones (see Table 1). Younger specimens, have a straighter frontoparietal suture (Supplementary Fig. S3a), whereas this suture is anteriorly curved in larger individuals (Supplementary Fig. S1b) of both morphotypes. The only apparent exception to this pattern seems to occur between specimens CM 9188 and NMNH 16368, as specimen CM 9188 already has a clearly curved suture, despite being slightly smaller than NMNH 16368. However, both belong to different morphotypes, indicating that the exact timing of change in the shape of the suture could be different between sexes. Another ontogenetic change occurs in the parietal, which becomes relatively larger anteriorly in both skull morphotypes in later ontogenetic stages. This change is better expressed in morphotype A than B, following the trend of larger skull sizes in morphotype A. In extant lizards, such as *Iguana iguana* (Supplementary Fig. S3c) drastic ontogenetic changes can be seen in the shape of the parietal, including variation on the shape of the frontoparietal suture, as previously described in the lacertid *Gallotia galloti* [13], and to a smaller extent in the gymnophthalmid *Neusticurus ecpleopus* [17].

Another feature that changes during the ontogeny of *Polyglyphanodon sternbergi* is the relative length of the posteroventral process of the jugal. This process is relatively shorter in juveniles in which it is unbroken (NMNH 16586, NMNH 427672) and does not reach the level of the quadrate (Fig. 1g,h). In the two adults in which the posteroventral process of the jugal is relatively complete (NMNH 16588 and NMNH 15816), it reaches the level of the quadrate, forming a complete lower temporal bar (Fig. 1i).

Ontogenetic variation in the formation of the lower temporal bar (LTB) is not exclusive for *P. sternbergi* among lepidosaurs possessing a LTB. Although hatchling *Sphenodon* already possess a complete LTB, fossil rhynchocephalians that usually possessed an incomplete lower temporal bar in early ontogenetic stages, such as *Planocephalosaurus* [18], *Clevosaurus* [19]—and possibly, *Diphyodontosaurus*[20]—have a complete lower temporal bar in older individuals. *Polyglyphanodon sternbergi* lacks replacement teeth, at least in the adult stage. However, it has a series of posterior

teeth that increase in size posteriorly. This indicates that teeth were added posteriorly and increased in size following the increasing size of the jaws throughout ontogeny, as observed in the posterior teeth of agamid lizards and additional teeth of rhynchocephalians [19,21-25].

Dietary habit in *Polyglyphanodon sternbergi*

There is a diversity of feeding habits among herbivorous lizards. For instance, *Corucia zebrata* processes most of the consumed plant material in its mouth, engaging in a significant number of intraoral bites[26]. Conversely, *Uromastyx* processes tough leaves by reducing them into small pieces, but has a low number of intraoral bites[26]. Finally, *Iguana iguana*, mostly crops leaves, with very little food processing in the mouth, swallowing most of the plant contents whole[27,28]. *Polyglyphanodon sternbergi*, has been proposed to be herbivorous on the basis of its highly specialized cropping dentition[28] and large body size, the latter being correlated with herbivory in many lizards[29,30]. The apices of the teeth of *P. sternbergi* bear multiple denticles that are similar to those of iguanine lizards, which are adapted for feeding on plant material, especially shearing/cropping leaves[28]. The lack of wear facets in the teeth of *P. sternbergi*, even in the absence of tooth replacement, suggests that there was a limited degree of food processing in the mouth before swallowing.

Detailed discussion on rejected hypotheses for the reacquisition of the LTB in lepidosaurian reptiles.

One of the earliest theories for the reacquisition of the lower temporal bar in lepidosaurs suggested that the LTB is an important feature for precise shearing action. According to Whiteside[20] and Fraser[19], during jaw opening the action of the *M. depressor mandibulae* upon a quadrate that is fixed both dorsally (to the squamosal) and medioventrally (to the pterygoids), but not laterally, would create torque upon the quadrate, twisting it posteriorly. This would interfere with the precise shearing that was important for the feeding mechanism of some early rhynchocephalians, such as *Diphydontosaurus*. Wu[3] suggested that the jugomandibular ligament was already present amongst these early rhynchocephalians, and therefore it would have prevented the posterior twisting proposed by Whiteside[20]. According to Wu[3], the bar would be a functional advantage as a lateral bracing mechanism to prevent anterior twisting of the quadrate during jaw closing. The resultant force of the temporal muscles in these taxa is directed anterodorsally and therefore would tend to twist the quadrate in that direction [3].

Nevertheless, this proposed lateral bracing of the quadrate is unnecessary for the proper functioning of a precise shearing system in lepidosaurs. The quadrate in rhynchocephalians has an extensive immobile contact both dorsally (with the suspensorium) and ventrally (with the pterygoid), which hold the quadrate in place against the action of the joint reaction forces acting upon it during biting. Early rhynchocephalians, such as *Gephtrosaurus, Diphyodontosaurus* and *Planocephalosaurus*, which had a fixed quadrate as just described, but also possessed a precise

shearing mechanism despite the lack a complete LTB (at least during most of their life) [18,20,31], indicate that a lateral bracing system was not necessary to maintain the proper functioning of the shearing mechanism. If any twisting of the quadrate took place in these taxa, that would have caused damage to the large articulation surface the quadrate has with the squamosal and the quadrate process of the pterygoid. This same inference can be applied to the opisthodontian rhynchocephalian *Priosphenodon* [32,33], which has a precise shearing mechanism and also lacks a complete LTB. Even in the extant *Iguana iguana*, in which the contact between the quadrate and the other skull elements is far less extensive than in rhynchocephalians, it suffices to prevent any rotation or twisting [27], therefore not affecting the precise shearing action of the teeth in this taxon. In some cases, such as in *Iguana iguana* and many borioteiioids, the ventrally expanded pterygoid flanges/ectopterygoids butting against the coronoid bone in the lower jaws must have aided in avoiding lateral displacement of the jaws, thus further contributing to precise shearing[34]. The latter system was proposed to operate in *Tianyusaurus*, and also applies to *Polyglyphanodon sternbergi*, which has similar ventral expansions of the pterygoid flange and ectopterygoids. Juveniles of *P. sternbegi* already present a perfect tooth interlocking system despite having an incomplete LTB, indicating the LTB was not necessary for such mechanism to operate, and being more likely to depend on the fixation of the quadrate, as well as the ventral expansion of the pterygoids and ectopterygoids.

Previous suggestions that the role of the LTB was to develop the translational movements of the jaw, observed during pro-oral shearing in *Sphenodon*[35], have also been discarded on similar bases. Fossil sphenodontians that display morphological features indicative of translational movement of the jaw (e.g. *Gephyrosaurus* and *Priosphenodon*) lack a complete LTB, rejecting that as an explanation for its re-development in *Sphenodon*[2]. Even if that was a valid explanation for species with pro-oral shearing, the morphology of the glenoid fossa in *P. sternbergi* (compressed antero-posteriorly), along with its interdigitating teeth, indicate *P. sternbergi* did not possess pro-oral shearing.

Despite contributing to the maintenance of precise shearing, the fixation of the quadrate by the suspensorium (dorsally) and pterygoids (ventrally) may not provide enough distribution of stress and/or compressive-tensional forces during hard biting for some species, as previously suggested[2,36]. This is likely to be a valid functional explanation for the re-development of the LTB, and we further discussed that in the main text.

Finally, it has also been previously suggested[34] that a cropping action involving a backward movement of the head would also induce the development of a lower temporal bar. According to this idea, the movement of the head would create a strong anteriorly directed food resistance force, which would tend to move the quadrate anteriorly. However, *Varanus komodoensis* uses backward movements of the head to rip off chunks of meat from its prey, even though a lower temporal bar is not present and the quadrate is fully streptostylic. This indicates there seems to exist no functional need for the presence of this bar due to backward movements of the head as utilized by squamate reptiles.

Consideration for other possible sequences of evolution

Another suggested sequence of evolution (as proposed by one of our reviewers) towards the condition observed in *Polyglyphanodon sternbergi*, would be the acquisition of a complete LTB before the acquisition of a fixed quadrate (a model with a complete LTB, but with streptostylyc, or movable, quadrate) in an ancestor of *P. sternbergi*. However, a complete LTB would naturally impose a natural restriction on the capacity of the quadrate to swing anteroposteriorly or mediolaterally (streptostyly). Therefore, the quadrate in such a condition would not be streptostylic by definition. Even in cases in which the LTB would be connected by soft tissues to the quadrate (and the quadrate was also connected to the suspensorium by a movable articulation), it is expected this connection would still restrict the quadrate movement. This restriction in movement can be seen, for instance, in the connection between the quadrate and the pterygoid in lizards with a streptostylyc quadrates. In such cases, the quadrate has a soft tissue contact with the pterygoid, and this connection is strong enough to avoid an independent displacement between the quadrate and the pterygoids. Thus, in typical streptostyly, both elements are displaced together[37,38]. In an extreme case, known as hyperstreptostyly, the quadrate does move independently from the pterygoid in some acrodontan taxa such as *Chamaeleo*[38]. However, that is caused by an even further degree of reduction of contact and connection between both elements. The quadrate in *Chamaeleo* does not have a pterygoid lappet for articulation with the pterygoid and has no rugose surface which could represent a region of soft tissue contact with the pterygoid (TRS pers. obs.). Therefore, it becomes clear that, for an independent movement of the quadrate in the presence of a complete LTB, the quadrate connection to the LTB would have to be by means of a relatively loose connection between both elements, which is unknown in any living or fossil reptile (in *P. sternbergi*, it is clear that this soft tissue connection was extensive by the very rugose surface on the tympanic crest of the quadrate, as illustrated above and in the main text). This would be further hampered by the fact that the quadrate would be connected to the LTB and the pterygoid simultaneously.

Even if the complete LTB of *P. sternbergi* represents a condition acquired previously in the phylogenetic history of North American borioteiioids, the first species to develop that condition would have to have an unrealistic set of conditions to allow a complete LTB to develop in conjunction with a quadrate that was still capable of swinging relative to the dermatocranium. First, the complete LTB would have to have developed before the strong contact of the quadrate to the pterygoid medially and the suspensorium dorsally, as seen in *P sternbergi*. Secondly, the quadrate would need to have a loose soft tissue connection to both the pterygoid and the complete LTB. Thirdly, this connection would have to be so loose as to compensate for the double contact of the quadrate (medially to the pterygoid and laterally to the LTB), and thus allow the quadrate to swing freely between the pterygoid and the LTB. Such a condition is unknown in the entire evolutionary history of reptiles. That may explain why all reptiles with a complete LTB, also have

a non-movable quadrate (including rhynchocephalians, crocodyles, stem archosauriforms, and stem diapsids such as *Petrolacosaurus*).

Considering the relatively enlarged contact between the quadrate and the pterygoid, as well as between the quadrate and the suspensorium, among Mongolian borioteiioids (e.g. *Gilmoreteius* and *Darchansaurus,* TRS pers.obs.), the concept of phylogenetic bracket would suggest that the condition in the lineage leading to *P. sternbergi* already had a quadrate with little or no streptostyly.

Therefore, we consider our model tested herein (a complete LTB with a fixed quadrate) a reasonable test not only for the actually known condition and evidence at hand (observed in *Polyglyphanodon*), but also as the most likely sequence of evolution leading to *Polyglyphanodon*.

FEA—additional notes and limitations

Bite forces. Bite, joint and muscle force input values are the most difficult aspects to estimate in FEAs designed to study the functional morphology of fossil taxa, such as *P. sternbergi*. Such difficulty arises due to a number of factors including the inability to observe the muscles directly (see Methods). Furthermore, multi-body computer model predictions of maximum bite force in lizards and in *Sphenodon* usually underestimate the real maximum bite force[39]. This is confounded by sexual variation in bite force: males may have bite forces up to four times to that of females, as in *Sauromalus*[40].

In order to address these issues, we used a range of values produced by scaling published values for the herbivorous lizards *Uromastyx hardwickii*[41] (see Methods) to the skull length of the models used in the FEA. Published bite force values for *U. hardwickii* seem to be lower than *in vivo* bite force values for similar sized specimens of another species of *Uromastyx*[42]. Therefore, additional values were used, 2x and 4x the initial scaled values, to observe whether they would affect our results.

Previously published *in vivo* bite force measurements indicate that adult male herbivorous lizards have stronger bite than insectivores, but do not differ significantly from omnivores[29]. Unfortunately, there are no published bite force values for adult male herbivorous lizards similar in size to *P. sternbergi* or *I. iguana* to provide estimates of bite force. Despite this, multiplying the scaled muscle forces for *P. sternbergi* by a factor of 4 seems to represent the best approximation of bite forces for an adult male of this species, as they are higher than the values obtained for males of the herbivorous lizard *Corucia zebrata* (SL = 50mm; bite force posteriorly on tooth row = 206N)[29], and similar to adult females of *Tupinambis merianae* (SL = 88m; bite force posteriorly on tooth row = 314N)[43]. Given the observed trend in other lizards species (see main text), it is reasonable to expect that males of *T. merianae* would have higher bite forces. We therefore, regard these estimates (SL = 70mm; bite force posteriorly on tooth row = 319N)—intermediate for bite force measurements between adult males *C. zebrata* and *T. merianae*—as

reasonable. Values for females of *P. sternbergi*, especially given our interpretation of sexual dimorphism for *P. sternbergi*, would be thus lower than the value estimate above based on adult males, and should be within the range of our lower scaling factors (direct scaling and 2x scaling factor).

However, the four models studied here displayed the same patterns of stress and strain distribution for each of the three different load scaling values, indicating that any discrepancy between our bite force estimates and real values for *P. sternbergi* would not influence our results.

Soft tissues. Limitations of the CT scans meant that soft tissue sutures were not included in the model. However, other analyses show that they are expected to dampen strain values[44,45]. Their exclusion, therefore, results in an overestimation of strain in each bone, which is more illustrative of the changes between the models. Additionally, overall stress is likely to be greater in our model because all muscles are activated simultaneously, as occurs in most FEA studies, thus representing peak strain values during biting. The biting point was placed posteriorly on the tooth row, where the specialized cropping teeth of *P. sternbergi* are located, which provides maximum biting force[46]. Finally, for a given bite force, herbivorous lizards have lower joint reaction forces when compared to omnivores and insectivores[47], and also have lighter skulls than carnivores (both with and without the influence of evolutionary history being considered), despite usually having higher bite forces[30]. All these indicate that bite force and overall stress conditions in our model are maximized, thus being a reasonable test to assess how stress conditions peak during hard biting could affect the skull of *P. sternbergi*, and how the presence of a complete LTB could affect skull mechanics.

For further comments on general limitations concerning FEA in biological organisms, we refer the reader to reviews on the subject[45,48].

Biting mode. We tested our models using bilateral rather than unilateral biting, a model previously used by other authors[36], for a number of reasons. Despite lizards usually using one side of the jaw to process food, adductors on both sides of the skull must be activated. Applying muscle adductor forces to one side only would imply that the animal is biting with considerable force on one side, while muscles on the opposite are inert, or entirely “relaxed”. This would be big a deviation of any reasonable biological assumption and modeling. Although there might exist some degree of imbalance among those forces, caused, among other factors, by asymmetries in the skull and muscle strength between both sides, testing using forces on both sides is certainly much closer to a realistic bite than applying to one side only. Assessing potential asymmetries on both sides due to skull shape was accounted by us by the usage of CT scans from a frozen extant lizard. Asymmetries in muscle load might provide even further accuracy to the model. We are unaware of current implementations of this model, however.

It is further important to consider that, depending on the size of the food particle, and the activity exerted during biting, the reaction force may actually occur on both sides. If the animal is grasping a branch or another larger plant material and pulling it, the material will be large enough to actually affect both sides of the jaws. The same applies for male-male biting for intraspecific competition, (which might actually be a likely possibility for *P. sternbergi* considering the sexual variation we observed). In fact, the information available from *P. sternbergi* suggests it did not process most of its food in its mouth, rather swallowing leaves right after cropping (see above in the subheading "Dietary habit in *Polyglyphanodon sternbergi*"). Therefore, a large proportion of the food reaction forces in the mouth was being produced by grasping branches, leaves, or other activities, such as intraspecific fighting among males (see our Sexual Dimorphism section, above). This indicates that, despite not affecting most of our stress/strain results, a bilateral bite is a meaningful replication of life situations that could be expected for *Polyglyphanodon sternbergi*.

Finally, as mentioned above, we aim to test the most stressful possible conditions to the skull, and check if the addition of a complete LTB would be functional in any way to reduce stress or strain. Applying the effects of a reaction force on both sides of the jaws replicates a more stressful condition to the skull rather than unilateral biting (as it doubles the food reaction force upon the skull), which may happen in a number of realistic situations for a lizard (see above). While we believe that some distortional effects to the skull may increase strain during unilateral biting, it is unknown how much the soft tissues could compensate for that. Having FE models that enable the testing of every soft tissue connection in the skull is a possible further development in methodology that may help in the assessment of this particular issue.

4. Supplementary References

1 Rieppel, O. & Gronowski, R. W. The loss of the lower temporal arcade in diapsid reptiles. *Zool. J. Linn. Soc.* **72**, 203-217 (1981).

2 Jones, M. E. & Lappin, A. K. Bite-force performance of the last rhynchocephalian (Lepidosauria: Sphenodon). *J. R. Soc. N. Z.* **39**, 71-83 (2009).

3 Wu, X.-C. Functional morphology of the temporal region in the Rhynchocephalia. *Can. J. Earth Sci.* **40**, 589-607 (2003).

4 Olsson, M., Shine, R., Wapstra, E., Ujvari, B. & Madsen, T. Sexual dimorphism in lizard body shape: the roles of sexual selection and fecundity selection. *Evolution* **56**, 1538-1542 (2002).

5 Ljubisavljević, K., Polović, L. & Ivanović, A. Sexual differences in size and shape of the mosor rock lizard [*Dinarolacerta mosorensis* (Kolombatović, 1886)] (Squamata: Lacertidae): a case study of the Lovćen mountain population (Montenegro). *Arch. Biol. Sci.* **60**, 279-288 (2008).

6 Schwarzkopf, L. Sexual dimorphism in body shape without sexual dimorphism in body size in water skinks (*Eulamprus quoyii*). *Herpetologica* **61**, 116-123 (2005).

7 Herrel, A., Spithoven, L., Van Damme, R. & De Vree, F. Sexual dimorphism of head size in *Gallotia galloti*: testing the niche divergence hypothesis by functional analyses. *Funct. Ecol.* **13**, 289-297 (1999).

8 Kratochvíl, L. & Frynta, D. Body size, male combat and the evolution of sexual dimorphism in eublepharid geckos (Squamata: Eublepharidae). *Biol. J. Linn. Soc.* **76**, 303-314 (2002).

9 Vitt, L. J. Sexual dimorphism and reproduction in the microteiid lizard, *Gymnophthalmus multiscutatus*. *J. Herpetol.* **16**, 325-329 (1982).

10 Carothers, J. H. Sexual selection and sexual dimorphism in some herbivorous lizards. *American Nat.* **124**, 244-254 (1984).

11 Vitt, L. J. Reproduction and sexual dimorphism in the tropical teiid lizard *Cnemidophorus ocellifer*. *Copeia* **1983**, 359-366 (1983).

12 Kaliontzopoulou, A., Carretero, M. A. & Llorente, G. A. Multivariate and geometric morphometrics in the analysis of sexual dimorphism variation in *Podarcis* lizards. *J. Morphol.* **268**, 152-165 (2007).

13 Barahona, F. & Barbadillo, L. J. Inter- and intraspecific variation in the post-natal skull of some lacertid lizards. *J. Zool.* **245**, 393-405 (1998).

14 Rieppel, O. Studies on skeleton formation in reptiles. Patterns of ossification in the skeleton of *Lacerta agilis exigua* Eichwald (Reptilia, Squamata). *J. Herpetol.* **28**, 145 - 153 (1994).

15 Maisano, J. A. Terminal fusions of skeletal elements as indicators of maturity in squamates. *J. Vert. Paleontol.* **22**, 268-275 (2002).

16 Evans, S. E. The skull of lizards and tuatara in *Biology of the reptilia* (eds. Gans, C., Gaunt, A., & Adler, K.) 1 - 347 (Academic Press, 2008).

17 Bell, C. J., Evans, S. E. & Maisano, J. A. The skull of the gymnophthalmid lizard *Neusticurus ecpleopus* (Reptilia: Squamata). *Zool. J. Linn. Soc.* **139**, 283-304 (2003).

18 Fraser, N. A new rhynchocephalian from the British Upper Trias. *Palaeontology* **25**, 709-725 (1982).

19 Fraser, N. C. The osteology and relationships of *Clevosaurus* (Reptilia: Sphenodontida). *Philos. Trans. R. Soc. Lond., Ser. B: Biol. Sci.* **321**, 125-178 (1988).

20 Whiteside, D. I. The head skeleton of the Rhaetian sphenodontid *Diphydontosaurus avonis* gen. et sp. nov. and the modernizing of a living fossil. *Philos. Trans. R. Soc. Lond., Ser. B: Biol. Sci.* **312**, 379-430 (1986).

21 Cooper, J. S., Poole, D. F. G. & Lawson, R. The dentition of agamid lizards with special reference to tooth replacement. *J. Zool.* **162**, 85-98 (1970).

22 Cooper, J. S. & Poole, D. F. G. The dentition and dental tissues of the agamid lizard, *Uromastyx*. *J. Zool.* **169**, 85-100 (1973).

23 Edmund, A. G. in *Biology of the Reptilia* Vol. 1 (eds Gans, C., Bellairs, A.d.A., & Parsons, T.S.) 117-200 (Academic Press, London and New York, 1969).

24 Edmund, A. G. Tooth replacement phenomena in the lower vertebrates. *Contr. R. Ont. Mus. Life Sci. Div.* **52**, 1-190 (1960).

25 Robinson, P. L. How *Sphenodon* and *Uromastyx* grow their teeth and use them in *Morphology and biology of reptiles* (eds. Bellairs, A.d.A. & Cox, C.B.) 43-64 (Academic Press, 1976).

26 Herrel, A. & Vree, F. D. Kinematics of intraoral transport and swallowing in the herbivorous lizard *Uromastix acanthinurus*. *J. Exp. Biol.* **202**, 1127-1137 (1999).

27 Throckmorton, G. Y. S. Oral food processing in two herbivorous lizards, *Iguana iguana* (Iguanidae) and *Uromastix aegyptius* (Agamidae). *J. Morphol.* **148**, 363-390 (1976).

28 Nydam, R. L. & Cifelli, R. L. New data on the dentition of the scincomorphan lizard *Polyglyphanodon sternbergi*. *Acta Palaeontol. Pol.* **50**, 73-78 (2005).

29 Herrel, A. Herbivory and foraging mode in lizards in *Lizard ecology: The evolutionary consequences of foraging mode* (eds. Reilly, S., McBrayer, L., & Miles, D.) 209-236 (Cambridge University Press, 2007).

30 Metzger, K. A. & Herrel, A. Correlations between lizard cranial shape and diet: a quantitative, phylogenetically informed analysis. *Biol. J. Linn. Soc.* **86**, 433-466 (2005).

31 Evans, S. E. The skull of a new eosuchian reptile from the Lower Jurassic of South Wales. *Zool. J. Linn. Soc.* **70**, 203-264 (1980).

32 Apesteguia, S. & Novas, F. E. Large Cretaceous sphenodontian from Patagonia provides insight into lepidosaur evolution in Gondwana. *Nature* **425**, 609-612 (2003).

33 Apesteguia, S. & Carballido, J. L. A new eilenodontine (Lepidosauria, Sphenodontidae) from the Lower Cretaceous of Central Patagonia. *J. Vert. Paleontol.* **34**, 303-317 (2014).

34 Mo, J.-Y., Xu, X. & Evans, S. E. The evolution of the lepidosaurian lower temporal bar: new perspectives from the Late Cretaceous of South China. *Proc. R. Soc. Lond., Ser. B: Biol. Sci.* **277**, 331-336 (2010).

35 Schaerlaeken, V., Herrel, A., Aerts, P. & Ross, C. F. The functional significance of the lower temporal bar in *Sphenodon punctatus*. *J. Exp. Biol.* **211**, 3908-3914 (2008).

36 Moazen, M., Curtis, N., O'Higgins, P., Evans, S. E. & Fagan, M. J. Biomechanical assessment of evolutionary changes in the lepidosaurian skull. *Proc. Natl. Acad. Sci. USA* **106**, 8273-8277 (2009).

37 Frazzetta, T. H. A functional consideration of cranial kinesis in lizards. *J. Morphol.* **111**, 287-319 (1962).

38 Iordansky, N. N. Evolution of cranial kinesis in lower tetrapods. *Neth. J. Zool.* **40**, 32-54 (1990).

39 Curtis, N. *et al.* Comparison between *in vivo* and theoretical bite performance: Using multi-body modelling to predict muscle and bite forces in a reptile skull. *J. Biomech.* **43**, 2804-2809 (2010).
40 Lappin, A. K., Hamilton, P. S. & Sullivan, B. K. Bite-force performance and head shape in a sexually dimorphic crevice-dwelling lizard, the common chuckwalla [*Sauromalus ater* (= *obesus*)]. *Biol. J. Linn. Soc.* **88**, 215-222 (2006).
41 Moazen, M. *et al.* Assessment of the role of sutures in a lizard skull: A computer modelling study. *Proc. R. Soc. Lond., Ser. B: Biol. Sci.* **276**, 39-46 (2009).
42 Herrel, A., Castilla, A. M., Al-Sulaiti, M. K. & Wessels, J. J. Does large body size relax constraints on bite-force generation in lizards of the genus *Uromastyx*? *J. Zool.* **292**, 170-174 (2014).
43 Gröning, F. *et al.* The importance of accurate muscle modelling for biomechanical analyses: a case study with a lizard skull. *J. R. Soc. Interface* **10** (2013).
44 Kupczik, K. *et al.* Assessing mechanical function of the zygomatic region in macaques: validation and sensitivity testing of finite element models. *J. Anat.* **210**, 41-53 (2007).
45 Rayfield, E. J. Finite element analysis and understanding the biomechanics and evolution of living and fossil organisms. *Annu. Rev. Earth Planet. Sci.* **35**, 541-576 (2007).
46 Moazen, M., Curtis, N., Evans, S. E., O'Higgins, P. & Fagan, M. J. Combined finite element and multibody dynamics analysis of biting in a *Uromastyx hardwickii* lizard skull. *J. Anat.* **213**, 499-508 (2008).
47 Herrel, A., Aerts, P. & De Vree, F. Ecomorphology of the lizard feeding apparatus: A modelling approach. *Neth. J. Zool.* **48**, 1-25 (1998).
48 Richmond, B. G. *et al.* Finite element analysis in functional morphology. *Anat. Rec. A.* **283**, 259-274 (2005).
49 Conrad, J. L. Phylogeny and systematics of Squamata (Reptilia) based on morphology. *Bull. Am. Mus. Nat. Hist.* **310**, 1-182 (2008).

5. Supplementary Data

Data matrix of Conrad[49], with modifications by Mo *et al.*[34], and with character-state scoring corrections for *Polyglyphanodon*, *Erdenetesaurus*, *Adamisaurus*, *Cherminsaurus*, *Gobinatus*, *Darchansaurus*, *Gilmoreteius* and *Chamops*:

nstates 16 ;

xread 'Data saved from TNT'

363 229

RHYNCHOCEPHALIA
100000?000010200000001[12]00000[01]00000000000[12]0000000[01]000101000000?0?00001
00?11000?000000000?000?100200000000?000000000000001?10000?00000000000000000001
000000000?000110001000??0001??0010[01]300?01???00000???0000?0000000[01]0[01]0000[
01]0000000000010?0[01]01?020[01]000100000000?01000000000?000000?0000000001000000
01000001?000000?00????000?000000000??????00?0000?00???0?000100[01]00?0???10?

Huehuecuetzpalli
2100?1?000012?00010010???00100??100000?02?00001100000110200?0?0?00?001?010012?0
00010000000001000000000????1?????????????1??????????????0????????????????????????11?00
0?001?????????00001?20?001??1?1?1011??00???????2000000?00?00000????1000101?020001
0000000000000000010?0010?0000?00000?000110?000??????????0???10?0???0??????????????
??????????????????????????????????????

AMNH_FR21444
?????0?000????????0???3??????001000000103?00001100000?002100100?1110000?00?10?100
00001??10001001100?????1000100?10100210000000000001100022010001100010?010?00?????
?????0?00??01100010002021?0001011010?000000?1011012000??00000??000??????????????
??
????????????????????????????????????

Gobekko
1000?0?000110?000000003??00000?0000000103??0002????001002100110?1110??000??03?10
000?011?1?0??0??????0????10110101?1?0?11000100?000?10?1?200???????10?01?001???3???
????0????????????0?2???????0??????????????????????????0????????0?????????????????????????
??
???????????????????????????

DIPLODACTYLINAE
100?00?000[01]10200000001301000000010000000003?00002????00110210011??1100010001?0
311[01]00???11?100???1??0110000?[01]0110001110021100010 0?00011001220100?00001000
100011?031100000000??00001010022320?000011010?000010?111101100000000000000000

10[12]0?1001?1101010000000100001011110101[12]0100?0100200?100110100031 1?00?1?1[0
1]0000?0?????010?101000100?????0000[12]100000000000???????????????

Tepexisaurus
101???????????000?????????????000???????????00110?0?01??????1????????1???0?2???0???1
?10??????00?100??????0???00011100211000000?000??00??20010?00?1?0?00?0?????11?00?0
0000??001??0?0002?00?0011010010?10??00?100?112000000000?00000???12001?01???00000
????003????0?01110?01?0??0?????000000?11???00?1???0????0?0?0?0??????????????????????
??????????????????????????????????

Eolacerta
100??00111110?0000001130?00000??000?00?010??0000000001000100????1??011001??20000
00?0?1??000??00?10011?????????????????2???????0?0????0?????????10???0000??00???1?????
0?000??000???00000011000000010010?10??0101000000300000000000000???12001221???00?
?0100000000000?01110?00?0100???00000?100110000001?????20??0??0?0???0?????????????
??

GYMNOPHTALMIDAE
1[01]10000?1[01]1100000000000030010000[01]1000000?0[13]?0000000001011021001[01]0?[01
]10011001202300000[012]00[01]0010000[01]02100100001[01]01100011100[12]1110[01]0000
0000[01]00002000002[01]0010000000001001020000 1001??000???100[012][02]020?001[01]011
01[01]1000001100000[01]0300000000[01]00000[01]10120012[02]1???00010100001000 00?00
1111000[13]00[01]0?1[12]0020000000201000?10[01]00?201[01]1000?0?000?0000402000?00?
????011000110110001?20110000010[01]00100

Chamops
?0????00?11???000?????30?1?00??0?????0??1???00?????1?????????????????????????????????
???0?????
?????00?02?????????????????????????????0400?[01]0000?00000????????????????????????????
??????????????????????????????????0??
??????????????????????

TUPINAMBINAE
10[01]000011111[01]200[01]000[01][01]30[01]1000000010000[01]0110000010001011021000
?0?1000110012[01]230000000000100020[01]021000?0001001100010100[12]111011010000 00
00002000001001100000000000010200000001??00[01]000000020210000101[01]010000000110
0000004000[01]0000001000000120112 01?000031010000100000000111100010100001002000
000010000000 10?00?20100000?0?????00004?2000100?????011000110110001?20110000010[01
][01]010[01]

TEIINAE
[12]00000011111020000000130010000000010000001[01]0000[01][01]0001[01]11021000???00
0011001002300000?0?000100??[01]0?100[01]?000?001100010110[12]1110110[01]?00000000

021?00?100[01]1000000000?010200000001??00000000002020?000[01]011011?000001100000
004000[01]000000000100120012?1?10003101000010000100111100[01]10[01]00101002000
0000100000010?00?20100000?0?????00004?2000100?????011000110210001?201100000[01]0
1[01]0100

Pseudosaurillus_sp.
??????01?1????????????300??10?????????????????????????00210010?????011???????????????
??1000??0
00???10002010?0000010010?10??00?1000010000?000000???00???120011?1???????01???????
???1???0??1????00??????????????????????????????
???????????????????????

Pseudosaurillus
??????01????????????????0??00??
???0?00??000
00?10?0201??0001010010?10??0????????0000?000000???00???????????????????????????????
??1???????010????0020???
????????????????????

LACERTIDAE
1000000000110000000000010[01][01]000000000000?0[13]0000000000[01]01[01]021000???11
00110012?1[03]?010??1?100100??[01]0?10011000?000100011100[12]1000[01]000?00000000
021?00?20011000000000?010200001001??00000?00002020?00000100100000001000000030
000000000[01]000000120011[02]1???00[01]10[01]0000100000?001110100[01]0000[01][01][1
2]000000000120000010[01]00?20100[01]00?0?000?000030200010001[01]?00[01]000011021
0001?0[01]110000000000100

Ornatocephalus
1001?00111110?00000011??0??001?0000?00??3???000011000100010?????1??0???01?????11?
??1?100??0???0?00011??0?????????????1???????????????0?????????????????????????????????0
?????000??000002?20?000??10010?1000???1001110000000010?00000????21???21???0?????0
????2??00????1???00????????00?00??00?????00???????20?00???????00??????????????????????
???????????????????????????????

CORDYLIFORMES
1000000111110000000000130010000001000000?0[12]00000000[01]0[01]01[01]021000?0?[01]1
00[01]1001[02]?2[03]?110?010100100010021001[01]0001001110011100[12]11000000000000
0000200000100[01]100000000000112000000000010[01]1010000[01]23[01]0?00000100100100
000?10[01]111[02]0000000[01]0000000?00120011[02]1???0[01]01010000[12]00000?00[01]11
0000[01]000000100000000020[01]000[02]101000201[01]0101111000[01]000010[12]000200?
????00[01]000000100001?0???00???????????

Sakurasaurus
?????001?1?????????????????01??00???0???????0??????0??002100100????011??12?0001100?
0?1??1?0?1??
???????00002?1??00???1?010??0000????????2000000101??0??????????????????????????????
???
??????????????????????

Parmeosaurus
101??0?000010?0000001030?1000???000000?010000110001?100100?10??1100?10011010??
001?0000?100?1001100?1???1101110?1?001011000?0?0000??0??0200?0020?110?00??00???1
1??0000000??000??01000??10?0001010010?10??00?101?1103000000000000000???1200?02???
?0?????????01?????????????????????????00????????000?????0?201001?1010?000????????????
???????????????????????????????????????

Xenosaurus
100000011111010000001130000000001000000101000000011001110210000?0?0100010010?101
010?100100100?1100100010001001100010101211000001000000000000200100100[01]1000001
000001120000100011030001?1000011100000012010000000?10010103000000000100000110120
001121?[01]00021010000020000000011000010000[01]0100000000000201000[01]1000001?100
0010000010000221100101??????0000?00001010110000?100??????????

Exostinus_lancensis
??????0??1??????0??????????000??????0????0??0?0011????10?10?1????????10?1??101?1???1
?1???1??0??11
??????100001?1?000????????????????????030000000?1??0?0??????????????????????????????
???1??????????011?????????????????????????????????
????????????????????

Carusia
1000?0011101010000000001300?000001000000102000000000100111021000?0?110001001??20?
0001000101100?110010001???10001110001001111000000000000000020000010?1100000100?
??11?000?10001103000?10000011000000120100?00000?00010120000000000100001???12?0??
?????0?0?????????????????????????1????????????????????????????1??????????010??????????
??

Restes
?????00111????00???0??????0000??000?00?010?0000001011?1?210?0?0???000100??????????
??????1?0?????10???????????????????????????1???
?????????0??0???????????????????????????0300000001??0?0???120010??????????????????
??????????????????????????????2?1??????????1??????????000??????????????????????????????
??????????????????????

Colpodontosaurus
???
???11???
???00001020?001????????????????????1200??10001???00?????????????????????????????????
???
?????????????????

Eosaniwa
301??01111??0??000000??????1???0100??0??10??000100?1??0?21000?0????0?1????????????
??????????????????1????01?0011110?111?????0?00??0?????????????0???0?00??00????0?000?1
000??1?????10000?2??001?0111?1?000?00?101?0212000000002??000???12102??1???0?1101?
?????1??????????????????????00?0??????????????????0?1????1?10???001??????????????????????
??????????????????????????????

Parviderma
??0???0?11??0????000??30?????????00?00?0???0??????????10210?0?0?0??001??10??0??????
0?0??1?00100?????1?????????0?000?0010000???????????????????????1??????????????????0?
0????????0?0?01020?00?1001111?00100???0????1202??11?01????0????????????????????????
??1???????0???20???????????????????????????
??????????????????????????

Bahndwivici
1011?00111110?0000000?10??0000??010?01?110?000001010110200?0?0?0??011001?010?0
0010000011?00100?100?????1????????????????????1???0??00????????????????????????11?10
0??000???????000001????00?101?010??0??0??000101020000?0001??000???12001021???00??
0100000000000?001???00?0?00???0000???00010100021???0????0?0?10?0??01??????????????
?????????????????????????????????????

Shinisaurus
10110001111101000000011000?00001010001[01]1100000000[01]01011020000?0?010011001
10100000110000110001101100110001001100010101100201001000000000021?1[01]0100110
00001000000112100000001200000000001[01]110000101101000000000?0001010200001000101
000110120010[12]1?1100110100000000000000011000010100001000001000010100021000021
?10000100000001000221100???0?????00?0?0000100?0100????????????????

Dalinghosaurus
101??00111110?0000000010?0?000000100011????00000101?11021000?0?0100?1001?010?0
0001000011000100?1001????10????0?100?10???0????000?0000??21????100?10?000??????10
?100?0000??000??00000??1120000011010?000?00?10??1?020000?000?00000???12001?21???
0??10100??0000001??1010?00?0100???0010000000100000?1???0????0?0?0?0???01?????????
???

'Saniwa'_Feisti
10?????0?011??00??0????????01???000?0???10??00100001?110200?????1????1001?0?0?000?
0000011?????????0??1???00?
????????01?0??????????????????????0?000???120?00?100???000???12?1?121???????0100000
2??00???01???0??0??????00?00??00????000?1?????1?1001?1?1??001?????????????????????????
???????????????????????????

Necrosaurus_cayluxi
?????0?0001?0??0??0?0?30???01??0?????0????????????????10200010??1??0010011110000??0
000???????1??????1??????????????2??1?????0??0??
?1??????10?010???001??????????????????????120200110020??00???1201?1?????1?1??1??0002
11????????????????????????0????????????????????????0?0??0??0001????????????????????????????
????????????????????????

Necrosaurus_eucarinatus
?????00??1?????????????30???01??01??????????????????????0?200?100????001?010010000??00
00???0??11
??????00?01?20??0???????????????????????1202001100????00????????????????????1??????????
???1??0?1?1????001??????????????????????????????
????????????????????

Proplatynotia
1100?0?01111010010000030 01?11?1010100000100000110101 0?00200?0?0?110001?010010?0
?0?0000??1000100?1????0001001101100000010001 0010??0?00??02000??10?110?????00?????
?????00000??????010001?2??00110011110001000?0001011200001?00201000???????????????
???1???????0???10?????????????????
??????????????????????????????????

Paravaranus
??0??1?0001?0?0??0010130???11??010000?0010000011000 1??10210?0?0?010001?011010?00
0?1000011002100?1001????101110110010101100000000000?00??0?000?01??0100000100???1
???????000???????01??01????000?0??????????00?100??112[01]0?0[01]?002???00????????????
??
?????????????????????????????????????

Parviraptor_cf._estesi
?????????0???????????????????????????????????????0???001?0?????000?01?0021???20?
1??0??01?
?????10?01????????????????????????????0??2?????????00????020001?1???0???01???0?20????
??
???????????????????

Parviraptor_estesi
1?0????0?0?????00?0??????0001?000???0??????????????????0?10001?0???0??10?00?00110002
001??0?0010???0?1???????1?111001?121??1?????????
???02020??0001??000????????????????????????????
??
??????????????????????

Parophisaurus
1010?001001?0??0?0000?30010000??000?00?0????001100000100200????????001001??10000
???00101100??00?10011???100?0000?000?0110000000000?0?????????????10???0?00???1??
0???00?012010??0000002111000?0110?0??00000?10110200000000000101000???120011?1????
????1??0020??????????????????????????00?????02??00????????21???1?10???001??0???????????
?????????????????????????????????????

Saniwides
1101?10111201000000013001?11?10101?00?010[01]?001100010100200?100?110001?01001
0?000?0000??100?1102100?????1?1110110?0?1010000100000000??021?0?01???100000100?
??11?100?0?00????????10001120?00110011110001000?10010112020011002010 00?????????
??0?1?????1???????????????????????????
???????????????????????????????????????

Telmasaurus
???0???000??????????????????????100?0??010100?11000111[01]?211?10???10001001001000
001?0000110002[01]001001?????????01100001010000100?000000??02001??1??1100000100?
??11?100???????????????0?????????????????????????????????????1?02??1?002???0????12?121?1????
???010??0????00????????0?????????0?00?????1100000?1???????????????????????????????????
?????????????????????????????????????

Lanthanotus
101?01?00011010000001012001?11?1110001?00101000110001010010001 00?010001001?0130
00001?000110001?1??01110001001111110101110000101000000000020011000011010101000
0011210000000 1?0100000000 1120?0011010111000 1000?10010112020011002010001 1012012
121?0102[12]10100000211000000 11[01]1003020000200000000011010002 10000?1?10000101
0010100024111 01?10001?11101?000120011?1?0??00???????????

Aiolosaurus
111??1?01?120100100001???1?110??100??0?01000?0?????1?1?0???????????0?1?????????????
???01?0???????0011000???????1??00?00?
???01100000001?20?00100000?1??01?00?100??11202001000?0100???????1?????????????????
??
??????????????????????????

Estesia
1110?0?00111010000000[01]3001?1100110001000110000010001010010001 0??010001001??1
30001??10101100?210110011000?001101110111210000101001000000020000010011001101 0
0???10?000?0000??01001000001?20?00??000111?001000?100?0112020011012010 00???1210
1????????1???????????????0???????????????0000???????????0?????????????????????????????
??

Eurheloderma
??1??011?1?????00?00?0?001001???1???????????0???????????????????????????10?130001?00
01???????????????????1???????????111?????1?1??0??1
2??????00001120?001??????????1??????????12020011012??0?0???12?011?1???0????????????
??1???0??1???020?????????????????????????????
???????????????????????

Gobiderma
1000?0111112010000000030?1001?01100000??1000000001010100 20000?0?0100010010?10?0
11?00010010001002100 1?000100110111001101 11?0?0?00100?0??02000?01??1101100100???
10?000?10000????00?00001020?001100?1110?01000?10010112020011002010 00???121011?1
???????01000???0000?????10?00???????????0????0010100021?????1?1?????0?0020?????????
???

Lowesaurus
??1???11?1?????????0??1?????0????1???????????????00?1?0?00100011??01?00???10?13???????
?1??????????????????????????????????????10???12
???????0?01?????????????????????????????120???11012???00????????????????????????????????
???1??????????120???????????????????????????????????
???????????????????

Paraderma
?01??011??1??10?????????????01???0?????1??1000??????
1??1??12?
?????000010?0??01??????????????????????1?0200110020100 0???1210???1???????????????0???
??1??????????120??????????????????????????????????
????????????????????

Primaderma
??1??01?????????1??????????0??1??????0????0???????????????????????????????????0?????1
0??1???
?????00010????01?????????????????????120?0?1100??1000???12?0?0???????1?????????????
???????????????????????????????????????1?????????120?????????????????????????????????
??????????????????

Ophisaurus_ventralis
10100000001101001000013000?0001100000010100000000000001002100100?0110010011?100
00???1?100100??00?10011000?0011000100010100010000000000000020010?100?100000100??
011?00000000??0010001000021100001011010??0??00?101102000000001010?00111?1201112
1?110?210100000002110000110000????0?0?4????????0??1??1??0000?211001110100000000?
21100???0?????????????????????????????????????

Ophisaurus_attenuatus
1000000000110100100001?00?000011000000001000000000000010010000?0?0110010011?100
00???1?100100??00?10011000?0011000100110100000000000000000021?10?1001100000100??
011210000000121?0??01000011100000011010??00000?1011020000000000100000110120111
21?0000210100000002000000110110????0?0?4????????2??1??1??0000?211001110100000000
2211001010??????????000??0???1?0????????????????

Dopasia
1000000000110100100001?00?00001100001010100?0000000001002000100?011001001??100
00???1?100100??00?10011000?001?000102111100000010000000000020010?1001100000100??
01121000000012000??01000011110000011010??00000?1011020?0000000101000110120111
21?0000210100002002000000110110?0100?0?4????????22?1??1??0000?211001110100000000
2211001010??????????000??0???1?0????????????????

Pseudopus
1000000000110100100001?00000001100000010100000110000010010000?0?0100?10011?100
001111010010000001100110001001000010001010001001000000000020010010011000001000
00112100000001202000010000111100000010101?00000?1011020[02]0000001010000111012
011021?1100210110002202010000110110?000010?4????????0?01001??0000?2110011101000
010002211001010?????0??0?000020000100?????00?????????

Bainguis
10???010?011??000?000??????00??0?00????010??0011000???002100100??1?0??001?0????00?
???0????????0??0011?????01100010????1110?????00???0????00???1????01000?00???1??100?
????????????0??0?????000?001010??0??01??0??1?01??00???????00????????????????????????
???0?201001?10?0?000????????????????????????
???????????????????????????

Anniella
100000?00011000000000110010000110 0?010001000001000100100100011 0?010001001?0?0?
00?0??10011001100011111000100110011110121000100100000210 0?2??10020111000001000
002121000000012000 1?01100020110010010010 0??0000?10110212000000001010 00?1012001
121?020?2101100??00200?0001???1??1??2???4????????2201??1??0?00?21111100?0?????000?
211011?10?????0??0?000000000100???????????????

Anguis
101000?000110000100000100000001100000000100000100000010010001 00?0100010?1??100
0000?1010110001001100110001001100010101[12][01]000000000000000000021?100100110000
0100?001121000000012001 1?00000211100100100100100000?101102120000000010100011 0
12001121?01002101100020020 0?000000?1?2000010?4????????2201??1??0?00?21100101?1?0
0000002211001?10?????0??0?000020000100???????????????

Apodosauriscus
?????000????????1??????????00??10???0?????????????????0?2?0?1?0?????01????????0??????
??0??12?
?????010002011000????0?0??????????????0000?000101????1??????????????????????????????
???0?21?001?1?1??000??????????????????????????????
????????????????????

Celestus
1000001000110100100000300000001100000000100000000000001000000 0?0?1110010010?200
00???0?100100??10?10011000?0011000101002110010000000000000020000?1001100010100??
011?00001000120300 1?0?000011100000110 10??0??00?1011020000000010101 0?11101200112
1?1100210100000001001000111000101000010010 0?100020000001?000?21110101010000000
02211001?10?????0??0?0??????????0???????????????

Diploglossus
101000100011010010 00[01]0300000001100000010100000000000001000000 0?0?1110010010?2
00000110?100100??00?1001100010011000101002100010000000000000020000?100110101010
0??0102000010001203001?000000111000001101 00?00100?10110200000000101010011101 20
01121?10002101000000010010001110001010010100000?10002010001100 00?21110101010?0
010002211001?10?????0000?000110000100???????????????

Ophiodes
101000100011010010 00[01]0300000001100000 0?010000000000001000100 0?0?1110010010?2
0000???0?100100??10?10011000?00110001010021000100000000000000200 00?1001100010100
??01120000100011000 01?00000011100000110 10??00100?101102000000001010100111 01200
1121?100021010000200100100011100020100102???1?????0201101??0000?211101010100001
0002211001?10??????????0?0??0???1?00111001110001000

Paragerrhonotus
?00??01100110?0000001130?00000??000?00?010??00000000??1020001?0?1??0010??0??????
?????????0??????????????1???????????01??????0????????0????????????????????????????0????
000????????10?001???000?0?????0???????10??020000000001??000??????????????????????
???[12]???0??1???000??????????????????????
??????????????????????????????

Gerrhonotus
1000001??01101000000[01]130?000001100000010100000000000001102[01]000?0?[01]110010
01??10?00010101011000100 11?01100010011100101000 1000[01]0000000000000020000010011
00000100?0011200 00[01]000120100001000011110000010 0100?00100?10110200000000010100
0011001200 1121?10002101000020020000001100011010000100000000020100001 0000?2110
011101000010002211001010?????0000?000000100100????????????????

Barisia
1000001??01?1000000011?00?000011000000101000000000000011020000?0?0110010010?10?0
00100010110001001100110001001110010100[12]1100100000000000000200000100110000010
0?001120000[01]00012010000100001111000000100100?00100?101102000000000001000011001
2001121?10002101000020020 0?00011000?1010000?000000?000201000?1?000?21100111010
00010002211001?10??????????0?0??????1?0????????????????

Abronia
1010001??0111000000001130??000011000000101000000000000011020000?0?[01]110010010?1
0?000100010110001000100110001001110010100210000000000000000002000001001100 00010
0?001120000[01]00012010000000001110000000110100?00100?10110200000000101000011001
2001121?10002101000020020 0?00011000?1010000?000000?000201000?1?000?21100111010
00010002211001?10??????????0?0??????1?0????????????????

Elgaria
1000001??011000000001130??000011000000101000000000000011020000?0?0110010010?100
?001000101100010011001100010011100101000111000000000000000200000100110000 0100?
0011200000000120100001000011 0?00000110100?00100?101102000000000101000011001 2001
121?100021010000?00200?0?011000?1010000?000000?000201000?1?000?21100111010000 10
002211001?10??????????0?0??????1?0????????????????

Helodermoides
1000?011111?0?000000?130?0000??0000000?010?000110101?1001000100?0100010010?101?
?1000010?100?2001100????000011000100?001???100000000000?0?00000[12]001100010100?
??1??0?000000??01100010000111000010100100000100?10110206000000000101000???12?011
?1?????????0????001?????????????010????00000??????????0?1???0?1?10?1110100221???????
???

Xestops
??????1??1?1????????????????0?????????????????????????0?10000?0???????0?1?????????????
??????????10????????????????0??0????1??????0??0?????????????????????????????????1???110
2???00101????0????????????????????????????????
??21???1?1????02???????????????????????????????????
??????????????????

Proglyptosaurus
1001?011?11???000?0?????00000??10?0?0????0?00?11010?011??00????????0?10?1??101?1??
1101???0???0011001?0?0??00?0?????????000???????0??0?????????????1??????????????????
?????????????0????????????0?????????????????600?00??0?01001??????????????????????????
???1?10?1?10?00221??????????????????????????
?????????????????????????

Paraglyptosaurus
??1???????11?1000???????????????????????????0?????????1010000?0????0010?10?101?????0
01???0??0?00??
???000000000111000?0010010?00??00?1011020500?0001010?000???????????????????????????
?????????????????????0?00???????????????????1???0??1???022????????????????????????????
????????????????????????

Glyptosaurus
?00??01111110??00000?????00000??00?000???0?00?00010??1101000100??1?0010010?101?1
???10100100??00?1001?????0??????100?00????????0??0??0?????????????10?????00???1?????
?0000?????00?1000011110000010010 0?00100?1011020600000010101001???1210?0?????????
???????0??????????????????????0?00?????????0???????0?1?1001?10100221?????????????????
??????????????????????????????????

Melanosaurus
??00?0111111??000??0?????????0?100?00???????000010010110100010 0????0010010?101?0?
???0101100?10001001???????????????0??0??????????00000?00?00????001?00?001?????11?00
0??0???????????100002111000?01?010??00?00???????0600000010101001???12?010?1????????
1???02?01???????????????????????0?????????00????????2???????1????02???0????????????????
?????????????????????????????????

Proxestops
??????[01]1??????????????30000?0??1????0???????0?????????0020001????1?001??1??1?0000
000?1????????????????????????00?100???1??
??1?020???1?0?0?1100????100?0?00??00???????0600000010 1??001???12?01??1???????01???
??1?001??0?0?021???????????????????????
????????????????????????????

Odaxosaurus
?0????00?111??00??????30000000?10???0???????00???0????002000100??1?0010010?1000010
0001??1?0???????????????????????????01??0?
?12??????100002?1?000?01??1??????00???????06000000101010 01???120?11?1?????????????
???21?001?1010?000???????????????????????
???????????????????????????

Peltosaurus
1000?0111111000000000130010000110000001010000000000010110200?10??110001001??100
0011010100100?1102100110?0100110101010011000100100000000000020000020?1100000100?
??11?1000100001030???0000011100000011010?000?00?1001020600000010101001???12?010
?1???0??????????????????111???1?0100??????????????????????00?201001110100021??0????
???

Arpadosaurus
??????1??????????????????????????????0?????????????????10100?1?0??1?001??10?101?0??0??
101??????????????????????00?1?0???1????????????0???????????????????????????????????????0?
?????0???00???????????????????????????0600??00101????1?????????????????????????????????
?????????????????????????????????????0?[12]1?001?10?0?021????????????????????????????
????????????????????????

Ophisauriscus
1000?010001101001000??3000000???000?0???1??0001000??010010000?0??1?0010010?10000
0111010?1000000?1001????1????????0???1????????0???????????????????10?????00???1?????
??0?????????????0????????????0??????00???101??2?200000000101000???12?1??21???0??1010
0?0?0??00???????????????????00?00???????1?00?1??00?21110111010?021??0?????????????????
?????????????????????????????????

Parasaniwa
??????[01]1?111??000????????0?00???????????????????????????1?2?0010??????0100100100010
00000??????????????????????????????????0????????????0??0??????????????????????????????????0
??11??????00001?20?000?????????????????????0200001100101000??????01?????????????????
???1??????????100?????????????????????????????
??????????????????????????

Dorsetisaurus
?????000111?0??0?0000?30?00000?0000?00??1?0000????????0021001?0????01100100000000
?000000100?1???????????????0?00??00??0????????0000000?0?2000001??1100000100????????
??1000?00001??00?00?1100011011011??00?00?1????10012000000???000??????????1????????
1???????????????????????????????????0????????????????????20??????????0??????????????????????
????????????????????????????????

ANILIIOIDEA
001011?0001[12]020[01]000001200000100100001??03?001????????10000003?1001?0030?110?3?0[02]?10?10[01]10?0???1?11??0010100111001[01]2?0[12]1100000000?002211?1??111101?1202?010000211210011[01]0011??00?10001320020112001 00??100[01]0?00010?100300000010[02]0001101200[01]221???0?21011100?2?201?002????2??1??2?????????????2??1?01??0000?2?1?0100?0?????0110411?1?0?0?????????0000?00???1?20??01???????????

Neomacrostomata
10[01]011?0001[12]02?1?0000[01]30000[02]100100000??03?001????????10?00003?1101?00[13]0?11[01]?3?[01]2??0?1001000???1?[01]1??001010121[01][01]0102?0[12]1100000010?002211?[01]??11110101202?01000102112000 1110?100000?00001[13]21[01]20[01]1200100???0000?00010?0003[01]0000[01]10[01]000110120[01]1221???0?210111[01]0?2?201?002????2??1??2?????????????2??1?01??0000?[12][01]1?0100?0?????0110411?1?0?0?????????0000?00???1?2[01]??01???????????

Xenopeltis
101011?0001202?1?000013000021001 00000??03?001????????10?00003?1101?0030?110?3?02??0?1001?01???1?11??001010111100102?011100000000?002211?0??11110101202?01000?021121001110?101100?0000132002011200100??00000?00010?10030000001000001101200022 1???0?21011100?2?201?002????2??1??2?????????????2??1??1??0000?2?1?0100?0?????0110411?1?0?0??????????0?0??????1?20??01???????????

Dinilysia
??1011?000??0??1?000012000?01001000?0??03?001????????100100?0???01?0020?111?3?00??0?100110101?0011??00?01?011010102011110000010 0?0022?1?1??1?00??11202?01??0??21?21001?100???????10001?210201120?100??00000?00010?00030?00001??000???12010221?????210?1????2??010??2?????2??1??2???????????????????1???

Haasiophis
200011?0001?0201?0000?200002100?000?1??03?001????????100000?3???01?0030?111?3?02??0?1001001???0011??00?010121000102?011100000010?0022?1?0????11???12?2??1?????21?20001110??00?00?000011200201121?101??00?00?00010?000300001010200 0???12010221????0?21?11100?2?201?002????2??1??2?????????????2?01000?0????????????0?0??

Pachyrhachis
1?1?1??0001???0?????????00???0??000????03???1????????100000?????0???030??1??3??2??0?100?001???0001??00?0????????102?01????000??0?002??????????1???12?2????????21?200?1110?????00??0001?20?2?112??101????000??0?10?00030?00001??000???12000221???0?211111?0?2?201??02?????2??1??2?????????????2??1000?0????????????0?0??

Eupodophis
10001??0001???01??0??????0?210?1000?0?????????????????0000????1?????????????????????
??01001???0001???????0121100102?1?1100????1??????????????1????????????????21?200011
10?????00??0?01?????0???10100??0000??00010?00030?0000?????????120??221???0?210????
???
???????????????????????????

Pachyophis
???
??0??1????????
???10001?211?0012???????0000????????0003?000?????000???120??2?1???0???1???????????0
??2????2??1????????????????????????????????????0?0?????????????????????????????????????
???????????????????

Wonambi
????1??0?0?????1??????3000021??10?????????????????????000000????0??0????011?3?02????
?0???0?????????????????????00?112???1110?0?110?0022?1?1??1010?101222?01?????????????11
10?1??????10001?21120??2???????10010?00010?0003?0000010?000???120002?1??????10111
0002?101??
?????????????????????????????

Bavarisaurus
100??0?000010?00?0000?30???0?0?0100?00??????0011000001[01]0010?0???0??0020?0??00?
?0???0?1010?00100100011??????????????????????????1?0??????????????????????????????????1????
??00??????????100?0?21?0????10??0??00?0??0?1??11200000000??1000???01??0?01?0?01000
00?0?0000????000??????00???0?0020?????????000?1?????????????0?0??????????????????????
??????????????????????????????????

Ardeosaurus
100??000?10?0?0000000030?00000?0000?00?03???001000000?00000?0???1??011001???0??0
0?010101100?100100011???1???????????????????0?????????????????????????????????????1??0??
????????????????0??????????????????????????000?00???00??0?????00????12?00?01???????010??
?0???????00?1??0?????????00?000?00?0??000???????2??????0?0?0?0??????????????????????????
?????????????????????????????

Eichstaettisaurus
101??0?000010?0000000????00000??000?00?03??000110000?110?10?100?0??0010000?20?01
0?0001001000100010001????10????????1??2????00?????????0????????????????????????10?0?0
???00???????0???0???????????1?????????0??00???0000000000000000????1000021???01??010
???00??00??01110001?000????00000?????0???00??????????????0?0??????????????????????????
?????????????????????????????????

Yabeinosaurus
?000?0011111??00?00??????0?00001000000??????001000000?002000100????0110012000000
010001??1000100??001????1?0??????00?00???????0????????????????????????????????????0?000
?0?00???????0?????????0????1?010????????00012100000000100000000???1200??1????0???01
0??00???00?????10?00????????00?000000?0???0000???0??????????0?0???0???????????????????
?????????????????????????????????

Scandensia
?0???0?0000?0?0???00?????????1???????0???????001???0???10000?10?????0???????0????????
?1??0??????????????????????????????????0?00??
??????100?2??1000?10????????0?0??000??0010?00??2??0000000?010??1?????0001010?0000?
?000??1110????0100???0??0000000????00?1????????????1101??000??0??????????????????????
??????????????????????????

Aphanizocnemus
???0?????01???0?????????????????????????????????0???00????10210?????????0?10?1?0??????????
0???00??10?10?1??????????????????????????????0??0????????????????00????????1???00?0????
??????????????????0011???????????0??????????2??????????????????120?1221???03210100010211
000?00010?00????????00?00??002?11100?0??
?????????????????????????

Dolichosaurus
??0?????????
??
????????????00?1?????1???????????????????0???????????????12001221???032?010?01011200??
?0000???11??0???0??0?????20?1?0??
???????????????????

Coniasaurus
?1???1???0??0??0?????1??0??1101?1??00???????????????1?11?210?0????00011??11??????????
?????0?????????????01??00????????1??11???????1????????????????????0??????????????????????????1??
????00?1001102???011???1?1???10??????????10120000001??000???120012???????????????????
????????00???
???????????????????????

Adriosaurus
1?00?????01???0?????????????110??0???00??????00???00???0?21???????????11001?010?00??0
0?001100??10???0?0??????????????????????????????0??0????????????0??????????????1?????????
??????????????????????1???????????????1?1????????0?1??0??0000???12001221???03??11?01102
1200????????????2????????0?200?0002?11000?0??
??????????????????????????

Pontosaurus
2100?1?000120?0000001130?11110??000?0?10????001100010110210?????0??1?1011?1?0?00
???0?00?100??00?1001????????????????12????????????????00???????????11?00???????1??011
?0110??1??0011000102??1001111101??11000?101?011202001000???000???12001?21???0311
1100110211000?0??10?10?0??????00200??002???000?0???0?1??001???0???00??????????????
??????????????????????????????????????

Aigialosaurus
2100?1?000??1?000??????????110??101?0??01???0111000101 1?200????????1110010010?000
00000??100011021001??????????????????????????0??0??0?????????1???1???????????11?0101
0?10??1????110001?2??10011?1101??01000?1001011202001000 1??000???12001221???0?110
10?110201000?????????????????00?00?1001101000???????????????????????????????????????
???????????????????????????????????

Opetiosaurus
1110?1?0001???000???1??????110??101?0??01???001100010110200????????0110010010??00
01000??1?0??10?1001???11?01??0
110??000??110001?2??10011??100??01000?1001011202001000100000???12?01221???????01
??11020100?????00?10????????00000?100????000?0???????????????????????????????????????
??????????????????????????????????

Tethysaurus
3100?1?000121?000?????????0110??10000??010??0100100101101010 0?0?000011?111000000
00100001100?11021001?010??????????????1?????????0?00?0?0?????1?0????100??1?????11?01
0?01????1????010001?2??10011001????0??00?101?011202001100100000???120012?1???0?1?
01???10201???????10?10?????????1?10?????2101110?????????????????????????0??????????????
???????????????????????????????????

Carsosaurus
???
??11?01???????????
???120012?1???0?1?01??000201000??
0000?1?0010010?00200000012?1000???
????????????????????

Goronyosaurus
310??0?0001111000???????000210??00001??020??00001001011010101 1??1000???01?000?00
011000??000011021001????0??????????011????100000000?0?0????1?01?0110000?100???1??
01??01??11??????10001?2??100??0???1??1???????????10021010001 00000???120000?1???0?1
?01???10201?????????????????????3210?????1???11???
???????????????????????????????????

Halisaurus_arambourgi
310??1?00010??000???????????1?????100???????011?0?0???1?10100?0??00111001?0?0?0001
0000011????????0?1?????????????????11???????00??0?0?????????000??1?01????????1?012?0
1????000??01000102??10011011010011000010010112020 01?10?00000???120010?1???0?1101
10110201????????????????????1010?????2??101???
?????????????????????????????????

Halisaurus_platyspondylus
3????1?000????0???0??1??????10????1?0????????????????110001?0???0?0?11011000000011
00001????1???????????????????????011????????0?000?0?0??????????????0?1?100???11?012?0?
10??0000011?0???????0011????????1000?100101?0??00???????00????120010?1?????1???1?1
10201???????????1?????????10?0?1000??1?01???
??????????????????????????????

Eonatator_sternbergii
3??????000?????????????10???11?????????????????????????111201???0?????11001?0?0?000010
00011?0??????0?1???11?012?0????
??????1100?1?2??10?110?1?1???1000?100101????0????????00????12001021?1?0?1???1??102
01???????10?10????????101011010221101????????????????????????????????0????????????????
??????????????????????????

Plotosaurus
310??1?0001212000000011??00021000001?1?0010000000100010111100 0????1??1220010002?0
0???0?000000??10?1001?010?0111001101011111101100?00000000020010?1?01110000000???
11?011?0110???????110001?2??10?110?101?01??00?100101100200101010 0000???12100021?
010?110110110201000??0100?10?0??????22202??10??????0???????????????????????????????
??

Globidens
310??1?0001211100???????000210?0101?1??0200?0001000101111101?0?0?0001210010000000
0110000010001102100 1?????????0011010111???1?0??0??0?0??????????1???11?000??????11?0
12??????????????1??????????????????????????????????1512001010100000???120002???10??110?
1?????????????????????????????1??
???????????????????????????????

Mosasaurus_lemonnieri
310??1?0001211100???????00021000101?1?0010000001100010111101010??0?002?011000000
0???0?000100??10?1001?????01110011?10111???100??000000????001??1??011000??00???1?
?010?01101?1????110001?2??1001101101?011000?10010110020010101000 00???12000221???
0?11011?110201?????0100?10?010????21203????2211110?0????????????????????????????????
???

Mosasaurus
310??1?0001211100??????0002100010101??0100001000001011110100???00002?001000200
001100000100??10?1001?01??01110011010111100 1?00?00?000???20010?10001100000?????1
1?011?0110111??0011000132??1001101101??11000?100101100200101010000 0???12000221?
1003110110?10201000?00000?10????????2120320102211 11??????????????????????????????
??

Moanasaurus
3100?1?0001211000??????0002100010101??0????0?????????1110100???00002?011?100?000
11000???00??102?001?????0111001101?1111001????0000?0????00???????11?001??????11???
??0110???????110001?2??100110?101?01??0??1001011002001000?00000???12000??????????
??
??????????????????????????????

Clidastes
310??1?0001211100??????0001100010100??0100001010001011120100?0?000011001000000
0011000001000110210 01?01??01110011?101110001?0??000000???2001?01???110000?00???1
1?010?0110111??0011000132??1001101101??11000?100?01100200100010000 0???12000221?
100011011011020100 0?0??10?100???????2120320102211?10?0????????????????????????????
??

Platecarpus
310??1?0001211000??????0001100010101??0200001000001011010100?0?00011?001110000
0?1000000100010021001101??01110011?101110001000?00?000???2001101?00110001?00???
11?0111011011???0011000132??100110?101??01000?100101100201100010000 0???12000221
??00?110?10010201000?0??10?00?0??????32203100122111 10?0??????????????????????0????
??

Prognathodon_overtoni
310??1?0001211000??????0001100010100??0?00001010001?11010100?0?00012?011000200
0???0?000000??10?1001?01??0111000101011 10001000??????????????????????????1?00???11?
012?011011?????10000132??100111?101??11000?100?011002011010100000???120000?1???0
?110110??????????????????????????32203?0??????11?????????????????????????????????????
???????????????????????????????????

Prognathodon_solvayi
310??1?0001211000????????001100010100??0?00?0011000101101 0000?0?000001011000200
0011000000000110210 01?????0111000101?1110001000000000 0???2??1001?01110001?00???
11?0?2?0110111??0010000132??1001111101??11000?100101100201101010000 0???12000221
?110?110110??????000??????????????????32203?0??????11?????????????????????????0???????
???

Plioplatecarpus
310??1?0001211000??????00011000101O1??0?0??0?0100???11021100?0?0001110110102000
01000000100?11021001?010?0111001101?1110001???0000000????001001?01110001100???1
1?012?01101100100110001 12??1001100101??01000?100101120201100010000O???12000021?
110?11011??10201000????10?00????????322031???22?1????????????????????????????????????
??

Ectenosaurus
310??1?0001111000??????000110??101?00?0200?010000010110111 00???1000110?10000000
???0?00?000??10?1001?????????????0??????????????0000???200???1???1??001??????11??12
?011011???00110001?2??100111?101???1000?10??01120201100010000O???12000221?1?0?11
01????????000????10?10????0???3220310?1??1???
?????????????????????????????????

Tylosaurus
310??1?00012112000001 1??0001100010101??0100001000001011010000?0?000111001100000
0010000000100011021001101??011100110101111101000?000000???20011010001100001 00???
11?111?0110111??0011000132??1001100101??01000?1001011002001000100000???1210[01]2
21?1000110110010201000?0??0011000??0???12202?0002211110?0???0????0?1??00???00??0?
??

Hainosaurus
310??1?0001111200??????000110??10101????0??00010?01011000100?0?0001110011002?00
011000??100011021001?????0111???????111???????0?????0????00??????????00?000???11?11
0?01??111????110001?2??10?11101?1??0100??100?01100200100010000O???120002?1???0?1
?01????????000????00?10????????12202?000???
??????????????????????????????????

Lakumasaurus
310????00011?1200???????00011000101?1??0?0?0?????????11?102?0?0??0??110011?000000
10?00??10001102?001??11??10?0
1????001??110001?2???0???10101??0?0?????????100200101010000O???120002?1???0???????
0102??
???????????????????????????

Palaeoxantusia
??00?001111??????????????100?0?1100?00001000000?11000100210010??1??0?10?0?0?3?12?
?010100000?21021001????1????000?0100211000001?00??0?0???000??2????0?00?001???1???
???1?00?????01?10122010?001100101001000010000000040000000000?000????????????????
???
?????????????????????????????????

Xantusia
10000001111[12]000000000[01][23]00000000[01]1000000020000011[01][01]0001[01]02[01]0
0100?[01]1001100[01]2?20[01][01]1???10101000?210?00010000?10110001010021110[01][01
2]01000001000010000001001000000000?0011200001000??00101?10122110?0011011010?1000
110000000040000000000000000000120010?1?1100110?0000?00?00?00111000?10000???000000
0000201000010100020101000?0??00?0101102000200??????????01??21???1?????????????????
?

Cricosaura
1000000011111000000001120010000101000000020000011000001102100110?1100110012?230
01???10101000?210?00010000?10110001010021100000100000100001 1?000210?10000000 1?0
011200001000??0011??10122110?0011011010?100011000000004000000000000000000120010
?1?0100110?0000?00?00?00111000?10000???0000000000201000010100020101000?0??00?01
01102000200??????????01??21???1??????????????????

Lepidophyma
100000?00011000000000002001100000000000000200000101000010010000?0?1110110002?231
11??210101000?210?000100001101100010100210001 3?1000001000010000021 0?100000000?
0011200001000??00100?10122110?0011011010?10001100000000400000000000000000012001
0?1?0100110?0000?00?00?00111000?10000???000000000020100001010002010 1000?0??00?0
101102000200??????????01??21??????????????????????

Polyglyphanodon
1000000011110?00000010300000000000100000010 0?00100000110000000000?1000?000100121
00000001??10022001100?????100?10??10101200???20000??0?0?0???????2????0010?000???1
??00000001??00000010002020?0001010000000??0110001001703000000?00000????2000221?
0?0??1010000[02]???000?01010?01?020????00200??001210000???????????????????????????
??

Erdenetesaurus
100???0??111??000???????0??000000?0?0??010??00110?0011[01]?20????0??????1001?0?0?0
001000000100210011000????1000??0010?01??1111200???00??????????????????????00???11?
000??001????????10002021000010100 10000???????????0410000000000000????????????????
??
????????????????????????????????

Adamisaurus
1000000?01110?0000000030010000000000000101?000000000000100010?0???100011?012022?0
001000200100?10021001????0001?000101 01201?1110000?0???????????????0?10?00???????1?
?000?0001??0001?000102000?0000010010?00??011000?0001140000000000000?????????????
???0????????????????
???????????????????????????????????

Cherminsaurus
1000?0000111??00000?00???1?000000?0?0??01000001?0000110?200?????????1001?0?0?00?
?0000??10021001100?????1000100010101211111 2000???0??0???????0?????0??????????1??00
0?0001??1??1??1000002100001010010000??0100001000410000000001000?????????????????
??
??????????????????????????????????

Gobinatus
100??00001110?0000000030???01??0000?001010000011 0001?100200?????1??001?01?020000
0010000010021002 1000????100010001010110???110000000000???????010??10?00??00???11
?0000000???000??010000020?000101?010000??01100000000 10000000?01000???12?0???1???
0?01?1?????????????1010?01????????00?0???
???????????????????????????????????????

Darchansaurus
101??00111??0?000?000040???00000010?00001000001101001100000?????0??0?1001?0?0?00
01000???00021003100??0??1000101110101200?002000000?0000???????2???10000???????1??
?00?0001??00001010002020?0001010010000??011?0??0?0400100000000000????????????01?
???????????????????????????????????0??0?????????00????????????????????????????????
???????????????????????????????????

Gilmoreteius
1000?00111110?000000004000?00000010000?01000001010001100000000?0?1000?10010022?
0000000?0010021003100 1?0?01000101010101201?11200000000000001000002?01100100000?
??10?000?1001??1??1??10002020?0001010010000??0110001000400100000000000???120002
21?010??10100000000000?01010?01?0100???0000000001210000?1????????????????????????
??

Sineoamphisbaena
1010100011110?00000000200??00010110010?02?0?001000001100100?11??0110?10?00?23?0
1???100??100?1?001001?00??00010001010020????3?0000?1?????????00?0??1000000?????10
?000000?????????01010??20?0????1?0?0??0??0??000?000104000000?00000???120002?1???0
01??1????????00?????1?????0??????0020???
??????????????????????????????????????

Bipes
101010?000121200000011300000011000?0?0?03?001????????100000?110?1100??0?1?0?3002
????101???1???1??0110000?0001000101?0201?013?1?10112210?0??1??00?111?0101111?031
120001000??1??1??10121301201???10010?000010?0001010000000000110000111220102 1??
?012101110010021 1?00000011031??0?0200200?1002??1?01??0101?20101000?0?????0100401
01?000?????????0?00?10???1?0????????????????

Blanus
000010?00011120000001030000000001010?010?03?001????????1000000110?1100010?110?300
2????101???1??????1??0000?0001000101?0201?013?1?10112210?0??1??20??11?2?01111?031
120001000????????10121300?01???10000?0?00??0000101000000000001100001?12201021???
0121011100100211?00000011031??1?0?0??1?????2??1?01??0101?20101000?0?????11004010
1??00010?0????0???????????????????????????

AMPHISBAENIDAE
[01]0[01]010?0001212[01]00000113000000010[01]0?010?03?001????????1000000110?110001
0?11[01]?3002????101???1???1??[01]110000?0001000101?02111013?1?101[01]2210?0??1??2
0?11[01]?0101111?131120001000??0[01]01??101[12]1301001???10000?000000?00010100000
000000110001?12201021???01210111001002110002?0012031??1?0??????????2??1?2???0[01
]01?2010[01]010?0?????[12]10040101?000010?0????0?00?10???1?00???0???????????

TROGONOPHIDAE
101010?000111210000011200000011000?010?03?001????????1000000110?1100010?110?300
2????101?0?0???1??1??0000?0001000101?02110013?1?101022[01]0?0??1??[01]0?111?000110
1?131120001000??0201??10121300?01???10000?010010?0001010001000000?1000001?12201
021?020121011100120201?002?0012031??0?0??????????2????2???0[01]01?2010[01]0[01]0?0
?????110040101?000010?0????0?00?10???1?0????????????????

RHINEURIDAE
10101011111102[01]00000[01]13000010010000010103?0000110001010000000110?1100020?1
10?3002????101?0?0???1??0110000?000100010100201?013?1010112210?0??1?000?112?0101
111013112000[01]000??0[01]01?0101213210001001[01]000?000010?000101000[01]00000001
10000??12201021?0?0121011100120210?002????2??1??2?????????????2????2???0001?201010
00?0?????110040101?000010?0????0?00?10???1?00???0???????????

Cherminotus
1110?1?0001201000000000130?10110?1100?1?001000001100010100200?100??100010010010?0
00110000110001001100 1?0?01?0110110020?1100000010000??00?02001?01??1?01010100???
11?100?00001?000000000????0?0011000111000100??1?0101120200110020100 0?????0??????
??????????????????????????????1??0??????????????
??????????????????????????????????????

Zapsosaurus
1?0???0010????????????????????0??01??????10??00110001?110210????????0?1101?002?00??2
00001?0?1??01?00??????0001000001?121100000?0000000?0??0010????010?00?000??11?00
0?0000??00000010010?11000110110?0?000?00?0000200400?000000??000???12????????????
1??????01???
??????????????????????????????????

Anchaurosaurus
100??0?000110?0000000030?1?010??010000?010000011000 1?11021000?0?00000110100 02?0
000200001?0??????????????0??????????012????0?0000000?000??00??0?????0?000000???11?0
0000000??000??010011021?001101101 0000??00?00002004000000000000000???12?000?1?????
???10?000000???????00??0????????00?0?????0??000???????????????0?0????????????????????
????????????????????????????????????

Ctenomastax
100??00001110?0000000130?1101001010000?01?000011000111 10210?0???0000?1001?002?0
0001000011002100110010???00?10000?1?121???000000000?0????????00??010?000000???1
1?00000?????000???0001?02???001010010??0??00?000020200010000000000??????????????
???0??????????????????????
????????????????????????????????????

Temujinia
1000?00001110?000000??30?1101001010?00001000001100011110210???????0001001?002?0
00020000010021001 1001????0??0?000001?121100000?000000000?0001001??0?0?000000???
1??000?0000??010??010010021000110110?0?00??00?000?20040000000000??000?????????????
???
?????????????????????????????????????

Rhampholeon
000000201111[01]20000001130011000010100010 03??10000010001100 0000?0?000011001000
3?02??000100001???01000001??01001000001001111010000000002010001?0?0000?10?00??10
??041200001000??020???10101320?01???11000?000?0000001200401000000?000001101210?
?01?000101010010020000 0102?0010031??0?0?00100?10000010003??010?2?000000?0???0?0
00100001?00?????????000???????????????????????????

Brookesia
001?0020111?020000010030011000 0101?011003??10000010001100000 0?0?0000100?10023?0
100000000001???01000001??0100100000100111101000000000 2010001?0?0000?10?00??10??0
41200001000??02000?10101321201???12000?000?0000001200401000000?000001101210??01
?000101010010020000 0102?0010031??0?0?00100?10000010003??010?2?000000?0???0?0001
00001?001??????11000??????????0????????????????

Physignathus
000000?0001100000000010300110000001000000100100101000111020000?0?00000101100021
00001000000001???0200000000000001000001012111000000000000000000000000010000000000
0001?00000000??02000000101?21101???12010000??0000000200101100000?0?000??012?????
???100?1?000000?0?????000?????0
??????????01??????????????????????????????

Agama
100001????11020000000003001100001010000001001000110011110210000?0?00000100100022
0000100001011???010000000000001000001002111010000100000001010000000001001000000?
0011200000000??02001010101321001???10010?000000?00002001011000000?0?000000120000
01?000001010000020000100001010020000110000000?100110000011?100?1?000??????????0?
0????????????????????????????????0????????????????

Uromastyx
000001????12020000000103011100001010001103?0000[01]11000111021000?0?000001101000
2200000000100001???0200100000010010000010020211001001000000010000001000100100000
0?0011200000000??000000100013210001???110100000000?0000100101100000?0?0000001201
1001?100101010000020000100001100000100112000000?100110000011?100?1?100?????????
0?0?????????0?0????????????????????0????????????????

Phrynosomimus
00110021111100000000001030?1100001010?000010000011110111021000???00?0?1001??02?0
00000010??01???001000????0000100000100211001000?0000??0????1???????210?00?????????
??00?0000??000??010?00??0?000?010010??0??00?000?200101?00000???000????????????????
?????????????????01??????000??
???????????????????????????????????

Priscagama
1000?02111111??00000113011101001001?000001000000001101111?210?0???0000??001??02?0
001000101101???0110000???000010000?10111010100000000?0????00???0???10100???????1
??00??0000??000000?0000020?0001010010????00?00???00101??0000?00000??????????????
????????????????????????????????0???????????????????????????1??????????????????????????????
?????????????????????????????????????

Mimeosaurus
0000?02111110?000000003011101001010?000010000011110011102107????0??0?1001??02??
0000001??001???011000????000010000?1?120???10000?00??0?0?0?????1???10?00??00???1?
??0??0000??00000010?001????001010010??0??00??0??200101100000?00000???12?00???????
????1??1??????????????????????????????
????????????????????????????????????

AMNH_iguana
0000?00001110?0000000030?11010??010?00001000001100011110210 0????0??0?1101?002?0
0001000011002100 1?001????100????0??????1??0000???????00??000??0????????????????1??0
???0?00???????0???????????????1?010????????000020040000?000?00000???12000001??000??
0?0?0001??000??0111?00?0?00???00000?100010000010????????????????????????????????????
?????????????????????????????????????

CROTAPHYTIDAE
[01]0000100111100000000003001[01]00001010000101000000001011110210000?0?000001[01]
01200200000100001010??0010000000011000100000[12]011110000000000000000000001000000
10000000000011200000000??0100[01]010020321[01]0001011010000000?000020040010000
000100000012000001?000011010?000[12]0000000011100[01]1000001100000?100010000001
0[01]0001?100000?0?????0000100000?00?0?0??01?????????????[01]????????????????

IGUANIDAE
10[01]000001[01]1100000000003001000001010000[01]0100000110001[01]110[01]1000???00
0001001000[12]000???0?0000101?00?00000001?000100000101[12]1110000[01]?0000000010
1?00?[01]0011000000000?011200000000??000000100203[01]1000100110101?00000?0000200
40000000000000000012000101?100001010?000000000[01]000110011000001000000?1000100
00001000001?000000?0?????0000100000?00?0?0??00?????????????[01]0??00???????????

FMNH_polychrotidae
100??0????110000?000?030????????0???0???????001?010???1021?10??????0010?10?02??????
0?0??0?0??00??00???????001000????111???000?0??
???????????1???2??0????1??????0??0??000?200?00?00000?0000????12?00?01???0???010??00
3000011?11???00????0???00000??00000?000??????????????0?0????????????????????????????
????????????????????????????

Anisolepinae
1000000111110000000001030000000001010000000100000000[01]000110210[01]0?0?000001001
2002000???000000?0?000100000000?100100000101111101000000000000101??00000010000
000000011?0000[01]000??0[01][01]0101012002100011011010?000000?000020040000000000
000000012000001?1000010100003000000101111000[12]000010000000?100010000000?00011
?00000[01]?0??000000?10000???1?1????01??????????????????????????????

Leiosaurinae
1000000111110000000001030000000001010000[01][01]100000000000[01]11021000?0?0000010
012002000???000010?0?110100000000?[01]0010000010111110100000000000010[01]0100[02
]00010000000000011?00000000??011[01]0010120[01]2100011011010?000000?0000200[34]0
000000000000000012000001?100001010000[03]30000110111[01]00010000000000000[01]000
10000001?000[01]1?000000?0?????000?10000???1?1?????1??????????????????????????????

PHRYNOSOMATIDAE
10[01]000[02]00[01]1200000000103000000000101000000[13]00000[01]10001111021000?0?00
00010010002200[01]0000001000??10100000000100010000010121[01][01]0000000000000000
0010000001000000000000112000000000??0[02]0010100001210001001[01]010?000000?000020
0[04]0000000000100000012000101?100011010?00010000100[01]0100012000011100100?100
0[12]000000100[01]001?100000?0?????0000100000?00?0?0??0[01]?????????????[01]????????
????????

CORYTOPHANIDAE
100000001[01]1100000000010300100000[01]0100[01][01]001000000[01]1[01]00111021000?0?
0000010[01]1[02]10[02]0?00??00001[01]00?1[01]01000000000000100000201111101000000
0000010[01]?1000?001000000000001120000000??00100010020321[01]00[01]1011010?0000
00?0000200400000000000000000012000101?100001010?00[03][12]0000[01]001000000100000
0100000?100[01][01]0000000010001?0?0000?0?????0000100000?00?0?0??01??????????????
???????????????

Polychrus_marmoratus
100000[02]011111000000010?0?0?0000101000010100000011100011021010?0??000?1001?00
300000000001111???010000?0??1000100000201111101000000000000101?100200?10000000
000011?00000000??000??010120?2120010011010000??00?10012004000000000000000001200
0011?1000010100000?000001001110002000010000000?100010100001??10?1?1????0?0?????
000?1??????????????01?????????????????????????????

Polychrus_femoralis
100000[02]01111?0000000?0?000?0000101000010100000000100111021010?0?100001001000
200000100001111???010000000?1000100010201[12]1110100000000000010001001000100000
00000011200001000??00001?1002002100010011010?000000?0000200400000000000000000001
2000001?1000?1010000?30000110?11000020000?0000000?100000000001011001?000001?0??
0000001100000?01?1????01??????????????????????????????

Polychrus_gutturosus
100000[02]00101100000000103000?0100001000000100000010000011021010?0?000001001?00
200000100000111???010000?00?00001000002011011000000000000000?01???010?0100000000
00?11?000?0000??00101010020?20?00110110100000?00?1000200400000000000000?????????
???10?1?000?00?0?????0?0?1????
???

Anole_AMBER
100?00??????0?000000?030??????0?01??00??????000???0???1021010?0?0000???????????0???
?0?010??1??01?00?0???0011?000001?1211?0000?0??00?00??000???0????0?00???????1??000?
????????????10????????????11?????????0??00?200400000000?000000??12000??1??????10100
0003??000?00110?00?000????00000?????100000?1??00?1??00000?0???000?0??????????????
??????????????????????????????????

Anolis_heterodermus
?00000[01]11111000000000103000?0100101000000100100001101111021010?0?000001001000
200100000100010110010000?0??00111000101?121111????01000000101?0000000100000000
00?11?000?0000??0011?010120321201???1?010?00??00??0????0400000000000000?????????
??0?011?000000?0???0000?110000
??????????01??????????????????????????????

Isodontosaurus
000??0?00013100000000030?11000??010?00?0100000110000111021 0?????1??0011010002?0
0001000??000101010000??????????0???1002???0???00???0??00???????1????0??????????1??0
00?00????02000?1000010??0001011????????010000?100500000000000000????????????????
???
????????????????????????????????

Anolis_occultus
101000?0001300000000003000?0100101000000100000000000111021010?0?00000100100020
01000000000102100100000?00?00111000001011111000000000000000000010000001001000000
0011?000?0000??0311?010120120?00110110101000000?00002004000000000000000000012000
000?1000010100000300000100110000100000000000000?10000000001?0?011?000000?0???000
00?1?0?0??

Anolis_vermiculatus
101?000111130000000000300101100101000000100000001100111021010?0?1000010010100?
01000000010101100100000?00?00111000001011011100000000000000?01?1?000001000000000
0011?000?0000??00101010120321000100110101 00??10?0010200400000000000000000012000
000?100?010100000?00001??011100010000000000000?100010000001?0?011?000000?0???000
00?1?0?0??

Leiocephalus
000000001111010001000030000000001010000000100000110000111021000?0?00000100100020
00???000010?0?100100000001?100100000101[12]11100001000000000001?10010001000000 0
000011?00001000??03001?10120020?0010010010?000000?000020040000000000000000000120
000?????????????????1??010?1?00????????????0?0???
??0???0?0???????????????????????????????????????

Stenocercus
000000001111[01]0000000[01]010010000010[01]000010100000110001111021000?0?0000010
01000200000100001000100010000000000000100000101111101000010000000000001001001100
00000000011?00000000??00101010120020?00100110100000000?0000200400000000000000000
0012000001?0000110100000100001[01]00111?00100000 1100?00?100010000000?010?1?100?
00?0?????0000????0???0?1????00??????????????????????????????

TROPIDURIDAE
[01]0000000[01][01]1100000000[01]0[13]00100[01]0010100000000100000[01]1[01]0011110210
00?0?00000100100022000002 00001[01]001[01]00100000000110001000000101111100000 0[01]00
000000000100100[01]10000000[01]00011200000000??0[23]101010[01]20[01]20?0010010010
0000000?0000200[24]0000000000[01]00000012000001?10001101000001 0000?00[01]11[01]?0
110000[01]1100000??0001000000??1[01]0[01]1?100?00?0?????0000????0???0?1?0??00??????
????????0[01]10001010101001

Liolaemus
00000000111100000100101000?0000101000000100000110101111021000?0?00000100100022
000010001?1001100100000001110010000010111110100000000000000000100000?1000000000
0011?00001000??0011??1011002100010011010000000?000020040000000000000000012000
000?110?010100000100001??00110001000001100?00?100020000000?110?1?100??????????0?
00????0???0?0????00?????????????????????????????

Hoplocercus
0000002001110000000010111100000001000000400100110000111021010?0?00000100100020
0000100001010110010000?00010001000101011100010000000000000101?10010201000100010
0010?00000000??02001010000[01]20?000001101000000000?00012004000000000000000000120
00001?110?010100000100000100110000[01]000001100000?100120000000???0?1?000???????
???0?00?0000??

Morunasaurus
0000002001110000000000003010?00000010000001001001100000110210?0?0?1000010010002?
000100000101011000100000??1000100010101110001000000000000?01?110100?1000000000
0011?00001000??02000?1000002110010011010000??00?00002004000000000000000?????????
???0?1?000??????????0?00?0000
??

Enyalioides
000000201111000000000010[01]00000000101000000400100110000111021010?0?00000100100
0200000000000101011001000000000100010000010111100100000000000000101?10010001000000
0000011200000000??00000010000020?00100110100000000?00002004000000000000000???12
000001?100001010000010000110011000[01]1000001200000?1001200000001000001?000000?0
?????000?100000?00?0?0??01??????????????????????????????

Oplurus_qh
001?000111110000000000003001100001010000000100000110001011021010?0?00000101100020
000000000100????01?00000?110001000102011111000000100000000100000001000100000001?
0011?000?0000??00101010120000?00100110100000??00?000020040000000000000000012000?
?1?00??01???????1??????????0???10???0????????????????????00011?100??????????0???????
??????0?????1?????????????????????????????

Oplurus_cyclurus
001?000111110000000000103001100001010000000100000010101?11021010?0?00000101100020
0001100001001???010????0?11000100010201111101001010000000100000010001000000010
0011?000?0000??03001010100020?00110110100000??00?0000200400100000000000???120000
?1?00??01???????1??????????0???10???0????????????????????00011?100??????????0?0?????
????0?0????11??????????????????????????????

Oplurus_ql
001?0001111110000000103001100001010001001000000100000110200?0?0?000001001?0010
00000000??0001100100000???1000100010201111100001000000000?000?0010?0100000000?0
011?000?0000??0300101012012 0?0010011010000??00?0000200400000000000000??????????
??????????????1??????????0???10???0????????????????????0001??100??????????0?0????????
???0?????1??????????????????????????????

Chalarodon
??????0?11???????0??????????????010?0?0?1?000001000?1?1021010?0?????0110100020000 0
1000????????????????????00?1????????????????????0?????0?0??????0??????0?0?????????00?0?
00??0010101002012 0?0010011010000??00???????04?0???0000??000????????????1??????????
??1??????????0???20???0?????????????????????00011?000??????????0???????????0?0????11?
?????????????????????????????

Polrussia
000????0001???000?0??????00010??110?0???10??00110?01111021000????000?1101?00???0?
?10??000?1???00?000????0????0?0?01?01??000000?00?0?0???????????????0?00??01?????????
?0000??001??01002??20?0010011010?00??00??0??2?0000??0000???000???????????????????
???
??????????????????????????????

Igua
0?1????000??????????????????????????0?????????????????10?1???????????1101?002000????0
0???????????????????000100000????????????0??????????????0????????????????????????????
???00????????????????????????????????
???
??????????????????

Myrmecodaptria
0010?010?001??000?00?0???10000??010?00?03?0000110000111?010?11?????0?1001??20?01
1101?10010000?1??001????1?0?1?1??01?021???11000???0?0?0?2????0?????0?0????????11?0
00?0000??000??00010?120?000101??00?10???10000?001000000000?0?000?????????????????
???20?????1????10???????????????????
??????????????????????????????????

Eidolosaurus
??0???????????????????????????0??
???000
???0????????00????12?0??21????3?1110?110?0?000?0
???????????????0??00????2??0110???
??????????????????

SCELOTINAE
0000000000[01]10200000001300000001100000010100000000001110020000?0?1100010?10?2
00100021010010002002100110001001110012100211100000000000000200000200?10000000
010011200001000??0011??10012011200100010000?100000?10111100000000000100000010120
01021?1100?1011000????1000011?????3010000200200?1??0201000?10000?2011110111?000
0000?1020002?0??????00??????????????????????????????

ACONTINAE
001000000[01]0102[01]0000000[23]000010111000010001000001100010100000100?1110010
0100[12][03]0101[01]01[01]00010001[01][01]010[01]1100010011100121012111O1[01]100000
000000200000100010[01]0001001001121000[01]000010101?010122200?0010010010?000000
?[01]011010000000000000000??012001021???0??1011?00020000?0????????????????4???????
?[01]0?1??2??0000?2011110111?0000000?1020102?0??????????????????????000????????????
?

SCINCINAE
1000000101[01]10[02]00000001300100000[01]10000[01]01020000000[01][01]00[01]11021001
00?1100110?12?20[01]1000210100100020[01]010011000110111001[12]10021110[01]0000000
0000002000001001100O[01]00001001120000100[01]0100001?10112010?0000000000000100000
?101111000000000000100000?1012001021?11001101000000000000100111000110100100000200?1
00[01]201000[02]10000?20110101111000000001020002?0??????00?????????????01?100010[0
1]1[01]00[01][01]0

FEYLININAE
1010000000110000000100[13]0000100121000[01]01020001????????1000100100?1100010?10
?220101021010110001?1??001100011011101121?12111000010000000000200000210?100000
0001001120000100001 1?????10122?10?021?000010?100000?1011110000000000100000??012
001021???00?1010000?????000010???2?31??0?0????????????????1??0000?2011110111?00000
00?1020002?0?????????????????????0001110101111?00?

Meyasaurus
?000?00?11110?0000001?300????0??000?0??0????00000000001102100100?11?0010012020000
??00000?1000100010001???1????0??????0?1???1??????????0?????????????????????????11?000
??00011??????10002?10000?001?0?0??00?0100000010300000000000000???12001101?120001
010?00000?000?01110?0000100?0100200000000001000?1?????1??????0?0?0?0??????????????
???????????????????????????????????????

Slavoia
2010?00001[01]102000000002001?10011100010001000001000000010000000?0?110011001 1?2
000000110100100010001001????00001100121001111001 1100??1?000?200??02????0000?????
??11?000?1000??00001?00002?10?0011011000?100?00?10011110000000000001000???121010

11???0?0101000002??00???1110?00?010????00000??0?1???000?0??????????????????????????
???

Tchingisaurus
100???000111??000?00?????10000??010?00??10??00010001?100210?????????11?012020?000
01??20?1?02100?10001??11?000?0
001??0000101000002100000010010?10??0110000000410000000000000???????????????????
???
??????????????????????????????

Dibamus
101010?00012020000001130000101121000?0?03?001????????1000000100?1110030?11013?1
1100?101???1???1??1??000010011100121?12110002010000022[01]000??100001210?0101110
0031020001000??0201??10122?00?021?010010?000010?0011010000000000[01]00000111122
00021???0?21010100100011?002??????31??1?0????1?????[01]0?1?02??2000?2?111100?0????
?1?1000101??0??????????0000??0???1??1??0?01?????????

Anelytropsis
001010?0001200000000113000010012100000?03?001????????1000000100?1110010?10013?1
1????10001?0?1?1??1??0000?0011100121?1211100211010002200?0??100001210?010111000
3102??01000??0001??10122?00?021?010000?000010?0001010000000000[01]00000???122000
21???0?2?????00100011??????????????????????????????????????[01]00?2?111100?0?????1?1??
??

Eoxanta
1010?0?000020?000000?03001000101000000101000001?00?0?1002000100?110011??12?20?0
0??010100100020021001????1000110010100211101100000?010?0??000??10???0000??00???1
??????1000?????1??00002010?000?01?0?0010??01000000000000?00000???00????????????????
???
???????????????????????????????????

Hoyalacerta
100??0?0000?0??0?0???????0?000??000?????10??00?1???????021000????0000??????????????
0?0?????????0??00??????????????00??02????????????????0??????????????????????????1?000?????
????????????????????????????????????0000?000???0??00?00??0???12000021???0?00010??000?
???1?0??10?00????????00?00????????000???
????????????????????????

Globaura
100??0?00001000000000003011001001000000?01000001100001110210010??110011?012?20?0
100110100100010021001????100011001010021110000000?11000??00???00???0?00??01???1
1?000?0000??0001?010002110?0000011010010??0100000110300?00000?00000?????????????

??
??

Palaeosaniwa
??????1?11??????????????????????????0?????????????0????10100?1???????????1??13??0??1?0
101??0???0??00?????1???????????10?????????????0?0?0????0?1??1?0????0??????1?1????0??1
???????00?01??0????1?????1?0???00???????12?20011001??00????121121?1???????0?0?00020
1????????????????????00?00????1??1000?1?????1???00?1????20???????????????????????????
?????????????????????????

Heloderma_horridum
101?00111111010000000130010000001000101010000000101?1000000110?0100010010?130
00110?010100001?1??0110000101110111011121000020100100000021?1001001100001000
00102000000001100000000001121?0011000111[01]001000?10010112020011012010001001 2
101121?000011010000020100000010010010?10?0200000000011010000100000?1?1000010101
2000002311101010001?00111?000?10101110????????????????

Heloderma_suspectum
101?0011111101000000[01]0030010000001000101010000000101?1000000110?0100010010?1
3000110?010100001?1??01100001011101110111[12]10000201001000000021?1001001100000
1000001020000000011000000000001121?00110001110001000?100101120200110120100010010
2101121?0000110100000201000000100100010?10?020000000000110100001000000?1?100001010
12000002311101010001?00111?000?101011100??0????????????

Heloderma_texana
1?1??01111110????00??0???1?00001100?10?0100000010101?100000011???10001??10?13?01
????01??0?0?1?1??011?????0111011101?121000?200001?000??021??001????0000??00???1??
??12020011?12????0?????????????????????
???1???0??1???120???????????????????????
?????????????????????????????

Gonatodes
101000?000110200000000301100000000000103?00002????001102100110?1110010010?032
10100?011?10001?1??0110001110110000010021100010000001100121000001100100000011
00310000000000??0311?010122320?0010011010?000010?0111000000000000000000000010100?
001?120?01000000001000010011111?001010010?00000?1000200000311?00?1?1?0000?0???000
10?1???0???1??????00?0?????????????????????????????

Gekko
101000?00011000000000003001100000000000103?00002????001102100110?1100010000?032
10101?011?10001?1??0110001110110000010021100010000001100102000001100100010000 1
0031100001000??1??1??100223212001001010?000010?01110000000000000000000000010100?0

01???010100000000100001?11111?001010000100000?1000100000?11?00?1?100001000?00001
0?1010001?3??????00?1100000???0?0????????????????

Pachydactylus
101000?000120200000000301100000000000?03?00002????001102000110?10000100?0?0300
0100?011?10001?1??0110001110110000010021100010000001100122000001000100010001100
031000001000??1??1??10022321200100110100?000010?1111002000000000000000000010100?00
1?1102010000000100001011110100101001010000?1000200000311?00?1?100001000?00101
0?1??00???3??????00?1?00000??????????????????????

Teratoscincus
000000?00011000000000004011100000000000003?00002????001102100110?0100010010?032
01000?011?10001?1??0110001110110000001?02110001000000110012100000000001000100011
0031000000000??0011?010022321200100100010?000000?111100000000000000000000000101000
001???0101000000001000010111110?00?0??????00000?100?101000011?00?1?110100?0???0001
0?1??00???3??????00?11???????????????????????????

Aeluroscalabotes
100000?00011000000000003010?00001000100103?00002????001102100110?1100010012?0320
0100?010010001?1??0110000100110001010021100010000001100102000001100100010001 1?
?31000000000??1??1?010022321000100100010?00??00?01110023100000000000000000011200000
1?110?01000000001000 0?01??11?00????????00000?1000201000311?00?1?100?00?0???00000?
1???0???0??????0??1?????????????????????????????

Hemitheconyx
100000?000110000000000030110000010001001 03?00002????001102100110?1000010012?032
01100?011?10001?1??01100001001111 0101?121000010000001100122010000[01]0010001000
1100310000000000??0311?010022321200100110100?000?00?0110002300000000000000000001010
0?011?????0100000000100000?011111100????????00000?1001201000311?00?1??00000?0???00
000?10100?1?0??????0??1100000???0??????????????????

Coleonyx
100000?00011000000000003010?00001000100103?00002????001102100110?1100010012?0320
1100?011?10001?1??0110000100110001 01?1211000100000011001220000000[01]00100000001
1?031000000000??0011?010022320?00100100010?000?00?0111012000000000000000000000010100?
001?110001000000001000010111111001010000100000?1000101000311?00?1?100?00?0???000
00?1?100?1?0??????0??1?????????????????????????????

Paramacellodus
?01???01??11??000???????01000???0???
???????????????????????????????00??1000??
000???10000010?000?01?010?10??00?10??0101000000010000000???120011?1???0???010??00

0?????????????????????????????????????001?????????0?1?10??0000?????????????????????????
?????????????????????????

Becklesius
??????01??????????0001??010000??0???
??0000??00
001?10000010?0000011010010??00???????00000000000??000???12???????????????0????????
??
?????????????????????

Varanus_olivaceus
21000100011100001001013001111010100000?00111100010?12?100100011 0?01001101111100
00010000011000110210011 00?1011101100101210000201010000000200??0000?1010101000
0011?00000000??0100000000 01120?000101101100 01000?10110105120011002 01000???120?20
21????2?10100?0021100?00011??0020000??200000?100???0000??0000?1?1000??0?00?0?000
?????0???

Varanus_eremius
110001?000120000100001300111001010000??0111100110?12?1002000110?01001101110100
0000000201100011021001100?1011100100101210000 1010?000000002?0??0?00?1010101000
0011?10000000110100000000 01120?001101101100 01000?1001011202001100201000???120?2
021????2?10100?0021100?00011??0020000??200000?100???0000??0000?1?1000??0?00?0?00
0?????0???

Varanus_tristis
21000100011110001000113001111010100000??0111100?10?12?1002000110?01001100110100
0000000001100011011001100?1011100100101210000 1010?000000002?0??0?00?1010101000
0011?10000000110100000000 01120?000101101100 01000?1001011202001100201000???120?2
021????2?10100?0021100?00011??0020000??200000?100???0000??0000?1?1000??0?00?0?00
0?????0???

Varanus_prasinus
21000100011100001000113001111010100000??0111100110?12?1002000110?01001100110100
0000100001100011021001100?1011100100101210000 10100000000000021?1001000101010 1000
0011?000000001101000000000 1120?00??011011?00???????????????????????00????1201202?
?001???????????????????0111000????????00000?10011000???0???????10000??????????0??????
??

Varanus_salvator
3110010001120000100001300111101110000?0011110011 0012?10021[01]0110?010001001101
00000[01]000001100011011001100?10111001001012100001010000000000021?100000010101
010000011210000000110000000000 1120?001101101100 01000?1011011202001100201000110

120220???001??1??0????????????????????????????00?0?????????000???????1?1000??0?00?0?0
00?????0???

Varanus_prisca
?11???00?1?????01?????3001?11?1?????1?????????????????002120110??10001001111000??
?0?0011?0??????????????????????0????????????????????0?????0???0???????1?????????????0?
?1???????00?01????????????????????????????12020011002??000???120220?1???1???010?000
21100???????????000????00?0?????1??0000???????????????0???3??????????????????????????
???????????????????????????

Varanus_komodoensis
3110010001120000100101300111101110000?0011110011001[12]?1002000110?010011001101
00000100000110001102100110 0?1011100100101210000101000000000020011000001010101 0
00001121000000011000000000001120?0011011011000100 0?1001011202001100201000110120
22021?011221010000021100100??11?0020000???00000?1001100000?[01]?????1?1000??0?00
30?000?????0??

Varanus_varius
3110010001120000100101300111101010000?00111100110?12?1002100110?01001100110100
000000000110001102100110 0?10111111001012100001010000000000021?11000001010101000
0011210000000110100000000001120?0011011011000100 0?100101120200110020100 0?????????
???00000?100????000???????1?10000??????????0?????
???

Varanus_rusingensis
?????????????????????????1?11??01??0110010000???0?
0??00?0???
10??000001?20?0???????????01000?1001011502001100???000???120120?1???1???010000021
?00?????11?00????????0000?????1??0000?1??
?????????????????????????

Varanus_griseus
2100010001120000100101300101001010000??0111100110011?10020001100010011001101 00
00010000001100011021001100?10111011001012100001010100000000 02001?010001010101000
0011200000000110100000000001120?0001010011?001000?10010112020011002010001101 20?2
021?01122101000002110010001110002 0000?0200000?10011000000010000?1?1000010100?00
000?4111010101???21101?001121?11112????????????????

Varanus_niloticus
2100010001120000100111300101101010000?0011110011001 1?1002000110?01001101110100
0001000000110001102100110 0?1011101100101210000101010000000000 21?11010001010101000
0011210000000110100000000001120?0001011011000100 0?1001011512001100201000110 12012

021?01122101000002110010001110012000000200000?10011000000100000?1?1000??0?00?0?0
00?????0???

Varanus_exanthematicus
1100010001120000000111300101101010000?0011110011001 1?1002000110?01001101110100
0000[01]000011000110110011 00?1011101100101210000101010000000002001101000101 0101
000001120000000011010000000001120?0001011011000 1000?10010115020011002010001101 2
022021?02122101000002110010001110002 0000?0200000?10011000000100000?1?1000??0?00?
0?000?????0???

Saniwa_ensidens
1100?10001110?000001?130?1?110?100000??01111001100?1?1002000????0??0010010010?0
0010000011000110?100110??101110010020111000010 10?????000020????10???0?0??1?????1
???00?0?0011?????000001????00???1?011??01000?10110112020011002??000???12012021?01
12110?0?000211001000111?002???0?0200000?1001??000000???0????0?0?0?0?0?0?????????
???

Typhlops
001010?000110201?000001000?3??0??000???03???1????????10001003?0?01100100100?3?11
?00?101???1???1??1??001010011?21101?021000010?00?012211????11000?112?2?01010?031
120001100?10?11???0001320?20?1?10100?0000?0?000100000200?000?0201011012211221?0
20?210?110002?2110002????2??1??2??????????????22?1?2????000?2?110100?0????0111?????1
???0??

Liotyphlops
001010?00010?201?001010000?3??0??0000?003???00110?1??10010003?0?01100300000?3?02
??0?101???000????1??001010011?2110100213000 10?00?012211????11000?112?2?01010?031
?20001100?11??1???0001320?20?1?10110?0000?0?000100000200?000?0200011012211221?0
20?210?110002?2110002????2??1??2?????????????2????2????000?2?110100?0????0111?????1
???0??

Leptotyphlops
001010?000110201?001004000?0100??000???03???1????????10000003?0?01100200100?3?02
??0?101???1???1??1??00001001101 0101?021100011?00?012211????11000?112?2?01010?031
?20001100?10?01???0001320?20?1210100?000000?000100000 2000000?0210011012211221?0
20?210?110002?2010002????2??1??2?????????????2????2????000?2?111100?0????0111?????1
???0??

Pygopus
001000?00011010000001130[01]0?00000100000003?00002??????1100100110?1100010?00?03
?010001011?10001?1??0110000100110111110021100010000001100102000002100100000001
10031100000000??030??010122020?0010011010?000000?11010100000000000000000??12001

021???0001001000010010101010100?0031??0?0?4????????1??1001??1100?2?110100?0?????010
?10?000000???????0021000100000?0???1???????????

Aprasia
111000?000110220000000120000?00000100010003?00002??????1100000110?0110000?00013?0
1100?121?00001?1??1??000111011000111002100001000000011001021?1?0200010?2?0001100
31?10001000??000???10120020?01???11010?000010?100100020000000000000000??120010?1
??0???2?110100?0?????010??0??0??
?0????????02?000100000?????????????????

Delma
100000?0001102000000113010?00000100010?03?001????????1100100110?1110000000?13?0
10001011?00001?1??011000110011000101002110001010000110010200000110010?2?0001??
031?10000000??0011??10120020?001001101000000010?1101010000000000000000??????????
??0???2?110100?0?????010?10??1?
??0???

Pletholax
111000?000111200000001110?0?0100?100001103?00002??????1101000110?0110000?00013?0
10001021?10001?1??01100001??11??????0021??0?1??000011001021?1?0200010?000001100З
1?10000000??0301?010120020?00100100100000010?1111010200000000000000?????????????
???0???2?110100?0?????010??0??0???
0????????02?000100000?????????????????

Pedrerasaurus
1?0?000?1102??????00?????????0??00?00???????00??0?0??1002100000????0????1?0????10??
00000?????????00??????00??0???????????1???00??0??
????????100??????00??????????????0????????03000000?01??000????2????1????0???0?000?0??
?000??001??00?0?0????0??0?????[01]00??00?1?????????????0?0????????????????????????????
?????????????????????????????

Jucaraseps
000000?0001???00??000??0??0000???00000??????00??0?0??1000101100?0??0????1?0?3??1?0
1???00100010????????????00?????????????????????????0????????????????????????????????00?0?
?????????????0????????0???0??00?????0?000110??000000????00000???1200??2?0??0???0?0000
00?0??0??0?10?00????????00?00????1200?00?1?????????????0?0???00??????????????????????
??????????????????????????????

Tianyusaurus
2000?02011110?000?000030???000000100000011?00010000010100000000?00000?100100?0?0
000000000001001003000 1?000100010000010?201011100000?01000?100???20?010000?000???
10?011?0?01?????00?100?001???0000010?00?00?0100001000410100000001000???120?????0

??????01????????000??101??00?010??????????????????????????????????000000?????????????
???

Jiangxi_2
20???02011100?0000000?0011100000100000101?0000000?010100210000??0000?1001?0?000
0001000??1?02100?01010???00001010001?0203011??1000?0?00????????20??000200000???1
0?????????????????00000???????????????????????????0410100000300000??????????????????
???00?????????????????????
?????????????????????????????????

Tuberocephalosaurus
20000000010110?0000010000?01000??10100001100?000000000100000000000000001001?0?2?
000010000?110210030001?????000100?000?1201011???00????00??10????2???10???????????1
0?000?01????00????000???1000????010?0?????0??0?????04101000000000000????????????????
??0000?????????????????
???????????????????????????????????

Aprisaurus
10?0000011?10?0??0000000???00???0100001100?00000000110000000001??001011?0?2?0
000?000??1002100?0001????????????????????????????????0??010????????????????????10?00
0?0111??00???0000000100000100111001 0??0001????0041010000000000??????????????????
??0000?????????????????????
?????????????????????????????????

;

ccode

+[/1 0 -[/1 1 -[/1 2 -[/1 3 -[/1 4

-[/1 5 -[/1 6 -[/1 7 -[/1 8 -[/1 9

-[/1 10 -[/1 11 -[/1 12 -[/1 13 -[/1 14

-[/1 15 -[/1 16 -[/1 17 -[/1 18 -[/1 19

-[/1 20 -[/1 21 -[/1 22 -[/1 23 -[/1 24

-[/1 25 -[/1 26 -[/1 27 -[/1 28 -[/1 29

-[/1 30 +[/1 31 -[/1 32 -[/1 33 -[/1 34

-[/1 35 -[/1 36 -[/1 37 -[/1 38 -[/1 39

-[/1 40 -[/1 41 -[/1 42 -[/1 43 -[/1 44

-[/1 45 -[/1 46 -[/1 47 -[/1 48 -[/1 49

-[/1 50 -[/1 51 -[/1 52 -[/1 53 -[/1 54

-[/1 55 -[/1 56 -[/1 57 +[/1 58 -[/1 59

-[/1 60 -[/1 61 -[/1 62 -[/1 63 -[/1 64

-[/1 65 -[/1 66 -[/1 67 -[/1 68 -[/1 69

-[/1 70 -[/1 71 -[/1 72 -[/1 73 -[/1 74

-[/1 75 -[/1 76 -[/1 77 -[/1 78 -[/1 79

-[/1 80 -[/1 81 +[/1 82 -[/1 83 -[/1 84

-[/1 85 -[/1 86 -[/1 87 -[/1 88 -[/1 89

-[/1 90 -[/1 91 +[/1 92 -[/1 93 -[/1 94

+[/1 95 -[/1 96 -[/1 97 -[/1 98 -[/1 99

-[/1 100 -[/1 101 -[/1 102 -[/1 103 -[/1 104

-[/1 105 -[/1 106 -[/1 107 -[/1 108 -[/1 109

+[/1 110 -[/1 111 -[/1 112 +[/1 113 -[/1 114

-[/1 115 -[/1 116 -[/1 117 -[/1 118 -[/1 119

-[/1 120 -[/1 121 -[/1 122 +[/1 123 -[/1 124

-[/1 125 -[/1 126 -[/1 127 -[/1 128 -[/1 129

-[/1 130 -[/1 131 -[/1 132 -[/1 133 -[/1 134

+[/1 135 +[/1 136 -[/1 137 -[/1 138 -[/1 139

-[/1 140 -[/1 141 -[/1 142 -[/1 143 -[/1 144

-[/1 145 -[/1 146 -[/1 147 -[/1 148 -[/1 149

-[/1 150 -[/1 151 -[/1 152 -[/1 153 -[/1 154

-[/1 155 -[/1 156 -[/1 157 -[/1 158 -[/1 159

-[/1 160 +[/1 161 -[/1 162 +[/1 163 -[/1 164

-[/1 165 -[/1 166 -[/1 167 -[/1 168 -[/1 169

-[/1 170 -[/1 171 -[/1 172 -[/1 173 -[/1 174

-[/1 175 -[/1 176 -[/1 177 -[/1 178 -[/1 179

+[/1 180 -[/1 181 -[/1 182 -[/1 183 -[/1 184

-[/1 185 -[/1 186 -[/1 187 -[/1 188 -[/1 189

-[/1 190 -[/1 191 -[/1 192 -[/1 193 -[/1 194

-[/1 195 -[/1 196 -[/1 197 -[/1 198 -[/1 199

-[/1 200 -[/1 201 -[/1 202 -[/1 203 -[/1 204

-[/1 205 -[/1 206 -[/1 207 -[/1 208 -[/1 209

-[/1 210 -[/1 211 -[/1 212 -[/1 213 -[/1 214

-[/1 215 -[/1 216 -[/1 217 -[/1 218 -[/1 219

-[/1 220 -[/1 221 -[/1 222 -[/1 223 -[/1 224

-[/1 225 -[/1 226 -[/1 227 -[/1 228 -[/1 229

-[/1 230 -[/1 231 -[/1 232 -[/1 233 -[/1 234

-[/1 235 -[/1 236 -[/1 237 -[/1 238 -[/1 239

-[/1 240 -[/1 241 -[/1 242 -[/1 243 -[/1 244

-[/1 245 -[/1 246 -[/1 247 -[/1 248 -[/1 249

-[/1 250 -[/1 251 -[/1 252 -[/1 253 -[/1 254

-[/1 255 -[/1 256 -[/1 257 -[/1 258 -[/1 259

-[/1 260 -[/1 261 -[/1 262 -[/1 263 -[/1 264

-[/1 265 -[/1 266 -[/1 267 -[/1 268 -[/1 269

-[/1 270 -[/1 271 -[/1 272 -[/1 273 -[/1 274

-[/1 275 -[/1 276 -[/1 277 -[/1 278 +[/1 279

-[/1 280 -[/1 281 -[/1 282 -[/1 283 +[/1 284

-[/1 285 -[/1 286 -[/1 287 -[/1 288 -[/1 289

-[/1 290 -[/1 291 -[/1 292 -[/1 293 -[/1 294

-[/1 295 -[/1 296 -[/1 297 -[/1 298 -[/1 299

-[/1 300 -[/1 301 -[/1 302 -[/1 303 -[/1 304

-[/1 305 -[/1 306 -[/1 307 -[/1 308 -[/1 309

-[/1 310 -[/1 311 +[/1 312 -[/1 313 -[/1 314

-[/1 315 +[/1 316 -[/1 317 -[/1 318 -[/1 319

-[/1 320 -[/1 321 +[/1 322 -[/1 323 -[/1 324

-[/1 325 -[/1 326 -[/1 327 -[/1 328 +[/1 329

-[/1 330 -[/1 331 -[/1 332 -[/1 333 -[/1 334

-[/1 335 -[/1 336 -[/1 337 -[/1 338 -[/1 339

-[/1 340 -[/1 341 -[/1 342 -[/1 343 -[/1 344

-[/1 345 -[/1 346 -[/1 347 -[/1 348 -[/1 349

-[/1 350 -[/1 351 -[/1 352 -[/1 353 -[/1 354

-[/1 355 -[/1 356 -[/1 357 -[/1 358 -[/1 359

-[/1 360 -[/1 361 -[/1 362 ;

Ancstates

-0 -1 -2 -3 -4 -5 -6 -7 -8 -9

-10 -11 -12 -13 -14 -15 -16 -17 -18 -19

-20 -21 -22 -23 -24 -25 -26 -27 -28 -29

-30 -31 -32 -33 -34 -35 -36 -37 -38 -39

-40 -41 -42 -43 -44 -45 -46 -47 -48 -49

-50 -51 -52 -53 -54 -55 -56 -57 -58 -59

-60 -61 -62 -63 -64 -65 -66 -67 -68 -69

-70 -71 -72 -73 -74 -75 -76 -77 -78 -79

-80 -81 -82 -83 -84 -85 -86 -87 -88 -89

-90 -91 -92 -93 -94 -95 -96 -97 -98 -99

-100 -101 -102 -103 -104 -105 -106 -107 -108 -109

-110 -111 -112 -113 -114 -115 -116 -117 -118 -119

-120 -121 -122 -123 -124 -125 -126 -127 -128 -129

-130 -131 -132 -133 -134 -135 -136 -137 -138 -139

-140 -141 -142 -143 -144 -145 -146 -147 -148 -149

-150 -151 -152 -153 -154 -155 -156 -157 -158 -159

-160	-161	-162	-163	-164	-165	-166	-167	-168	-169
-170	-171	-172	-173	-174	-175	-176	-177	-178	-179
-180	-181	-182	-183	-184	-185	-186	-187	-188	-189
-190	-191	-192	-193	-194	-195	-196	-197	-198	-199
-200	-201	-202	-203	-204	-205	-206	-207	-208	-209
-210	-211	-212	-213	-214	-215	-216	-217	-218	-219
-220	-221	-222	-223	-224	-225	-226	-227	-228	-229
-230	-231	-232	-233	-234	-235	-236	-237	-238	-239
-240	-241	-242	-243	-244	-245	-246	-247	-248	-249
-250	-251	-252	-253	-254	-255	-256	-257	-258	-259
-260	-261	-262	-263	-264	-265	-266	-267	-268	-269
-270	-271	-272	-273	-274	-275	-276	-277	-278	-279
-280	-281	-282	-283	-284	-285	-286	-287	-288	-289
-290	-291	-292	-293	-294	-295	-296	-297	-298	-299
-300	-301	-302	-303	-304	-305	-306	-307	-308	-309
-310	-311	-312	-313	-314	-315	-316	-317	-318	-319
-320	-321	-322	-323	-324	-325	-326	-327	-328	-329
-330	-331	-332	-333	-334	-335	-336	-337	-338	-339
-340	-341	-342	-343	-344	-345	-346	-347	-348	-349
-350	-351	-352	-353	-354	-355	-356	-357	-358	-359
-360	-361	-362	;						

xgroup

;

agroup

;

proc/;
